# Synthesis and Reactivity of 3*H*-1,2-dithiole-3-thiones

**DOI:** 10.3390/molecules26123595

**Published:** 2021-06-11

**Authors:** Oleg A. Rakitin

**Affiliations:** 1 N. D. Zelinsky Institute of Organic Chemistry, Russian Academy of Sciences, Leninsky Prospect, 119991 Moscow, Russia; orakitin@ioc.ac.ru; Tel.: +7-499-1355327; 2 Nanotechnology Education and Research Center, South Ural State University, 454080 Chelyabinsk, Russia

**Keywords:** 3*H*-1,2-dithiole-3-thiones, synthesis, pharmacological activity, sulfurization, elemental sulfur, disulfur dichloride, 1,3-dipolar cycloaddition, ring transformations

## Abstract

3*H*-1,2-Dithiole-3-thiones are among the best studied classes of polysulfur-containing heterocycles due to the almost explosive recent interest in these compounds as sources of hydrogen sulfide as an endogenously produced gaseous signaling molecule. This review covers the recent developments in the synthesis of these heterocycles, including both well-known procedures and important novel transformations for building the 1,2-dithiole-3-thione ring. Diverse ring transformations of 3*H*-1,2-dithiole-3-thiones into various heterocyclic systems through 1,3-dipolar cycloaddition, replacement of one or two sulfur atoms to form carbon- and carbon-nitrogen containing moieties, and other unexpected reactions are considered.

## 1. Introduction

**Figure 1:**
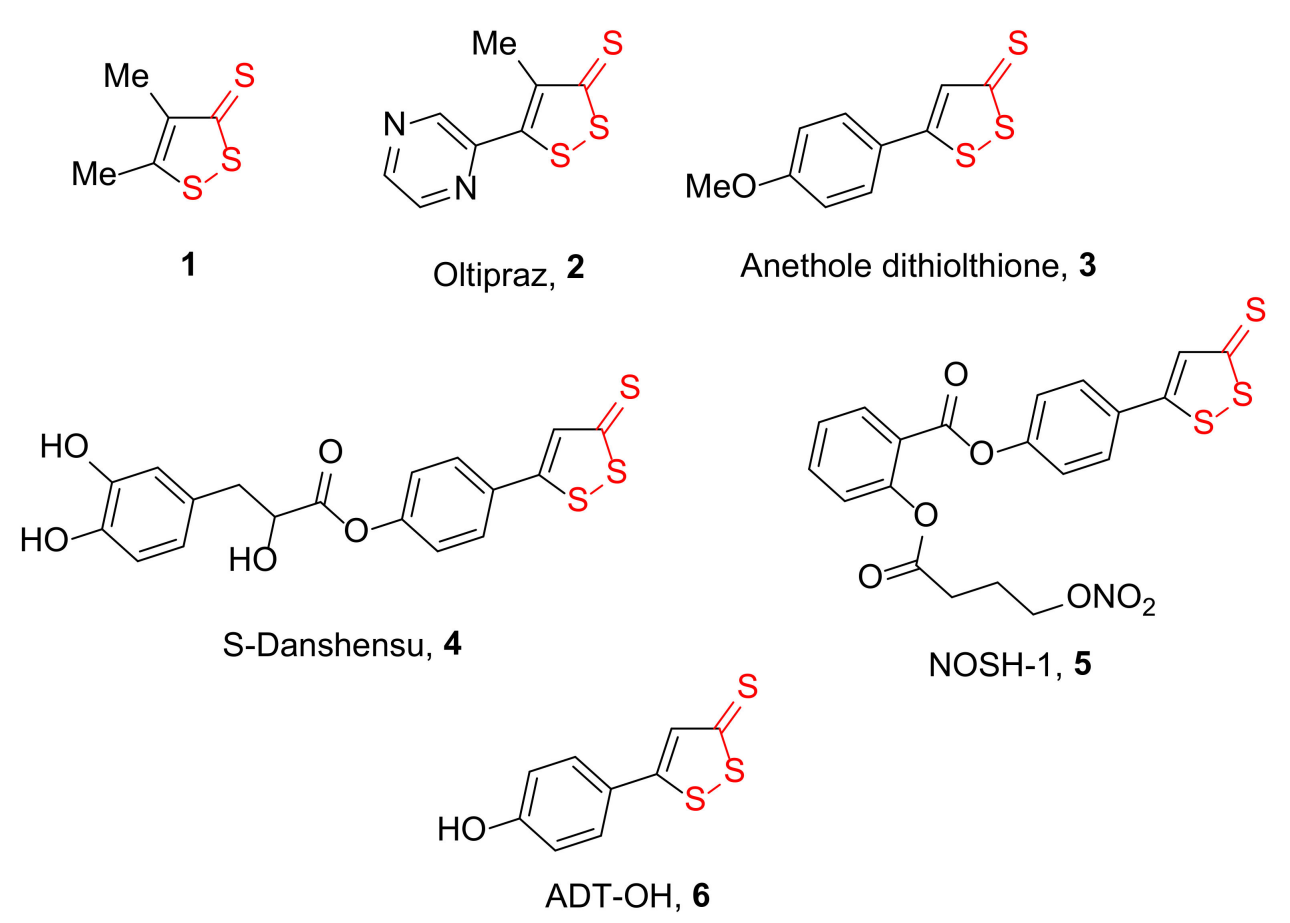
4,5-Dimethyl-1,2-dithiole-3-thione (**1**) and 1,2-dithiole-3-thione-containing drugs (**2**–**6**).

1,2-Dithioles have been an important class of sulfur heterocycles since 1884, when the first representative of this class, 4,5-dimethyl-1,2-dithiole-3-thione (**1**) was synthesized [1]. 1,2-Dithiole derivatives show many types of significant pharmacological activity, including antitumor, antioxidant, chemotherapeutic, antithrombotic and radioprotective properties [2,3,4,5,6,7,8,9]. In addition to the above applications, 1,2-dithioles show anti-HIV activity [10,11]. The 3*H*-1,2-dithiole-3-thione moiety in 1,2-dithioles occurs most commonly in commercial drugs such as Oltipraz (**2**) [12], anethole dithiolethione (ADT, **3**) [13], S-Danshensu (**4**) [14], and NOSH-1 (**5**) [15] (Figure 1).

Hydrogen sulfide (H_2_S) is considered to be the third endogenously produced gaseous signaling molecule, or gasotransmitter, along with carbon monoxide (CO) and nitrogen monoxide (NO) [16]. Cellular H_2_S biosynthesized by enzymatic and non-enzymatic pathways regulates important functions of the cardiovascular, immune, nervous, respiratory and gastrointestinal systems and is involved in a number of diseases, including Down syndrome, Alzheimer’s, and Parkinson’s diseases [17]. Although there are a great variety of H_2_S donors, one of the most extensively studied H_2_S donors are 5-(4-hydroxyphenyl)-3*H*-1,2-dithiole-3-thione (ADT-OH, **6**) and its derivatives that contain the H_2_S-releasing dithiolethione moiety.

The second reason for the interest in 1,2-dithioles is the rich chemistry of these compounds which until recently was associated mainly with 1,2-dithiole-3-thiones. For example, many 1,2-dithiole-3-thiones have been employed as precursors for the synthesis of tetrathiafulvalene vinylogues that enhance the nonlinear optical properties for the creation of organic electronic conductors [18], photoconductive materials [19,20], or semiconducting polymers [21].

Therefore, special attention was paid to the development of efficient and reliable methods for the synthesis of 3*H*-1,2-dithiole-3-thiones and to their reactivity. A number of reviews deal with the general aspects of the chemistry of 1,2-dithioles, such as the corresponding chapters in *Comprehensive Heterocyclic Chemistry II* and *III* [22,23] and other reviews that should be ranked among the most important ones [24,25,26]. No special review dedicated to the synthesis and reactivity of 3*H*-1,2-dithiole-3-thiones was previously available in the literature.

## 2. Synthesis of 3*H*-1,2-dithiole-3-thiones

There are several general methods for the synthesis of 3*H*-1,2-dithiole-3-thiones by sulfuration of 3-oxoesters, *iso*-propenyl derivatives, α-enolic dithioesters and related compounds, alkynes, tertiary isopropylamines, and other less advanced protocols. However, these methods still have some limitations, including drastic reaction conditions, poor yields and selectivity, low atom economy, or the use of hard-to-reach and moisture-sensitive sulfur containing reagents.

### 2.1. Synthesis of 3H-1,2-dithiole-3-thiones from 3-oxoesters

**Scheme 1:**
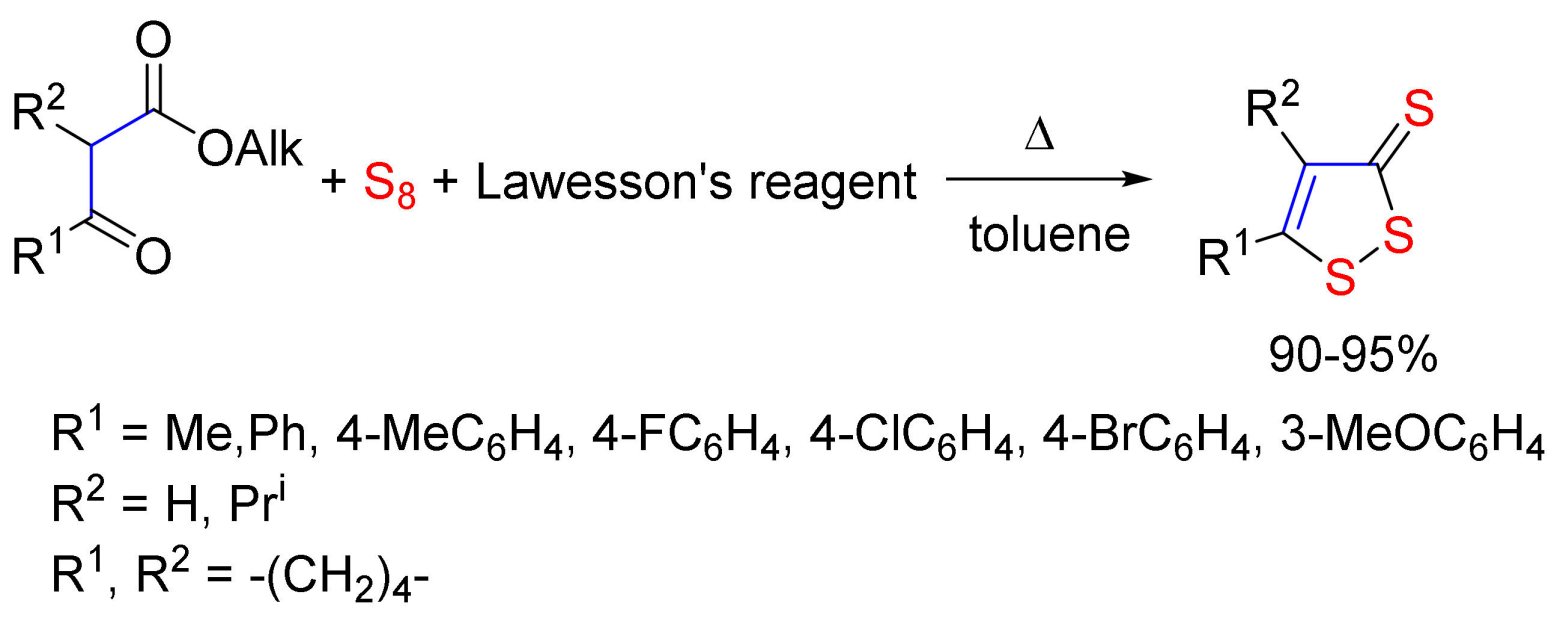
Sulfuration of 3-oxoesters by elemental sulfur and Lawesson’s reagent.

Sulfuration of 3-oxoesters is the most commonly used method for the synthesis of 3*H*-1,2-dithiole-3-thiones. Various reagents and conditions have been described for this reaction. This procedure was discovered by Pedersen and Lawesson in 1979 when unsubstituted and 2-monosubstituted 3-oxoesters were brought into reaction with a mixture of Lawesson’s reagent (2,4-bis(4-methoxyphenyl)-1,3,2,4-dithiadiphosphetane-2,4-disulfide) and elemental sulfur by refluxing in toluene to give the corresponding 3*H*-1,2-dithiole-3-thiones in nearly quantitate yields (Scheme 1) [27].

**Figure 2:**
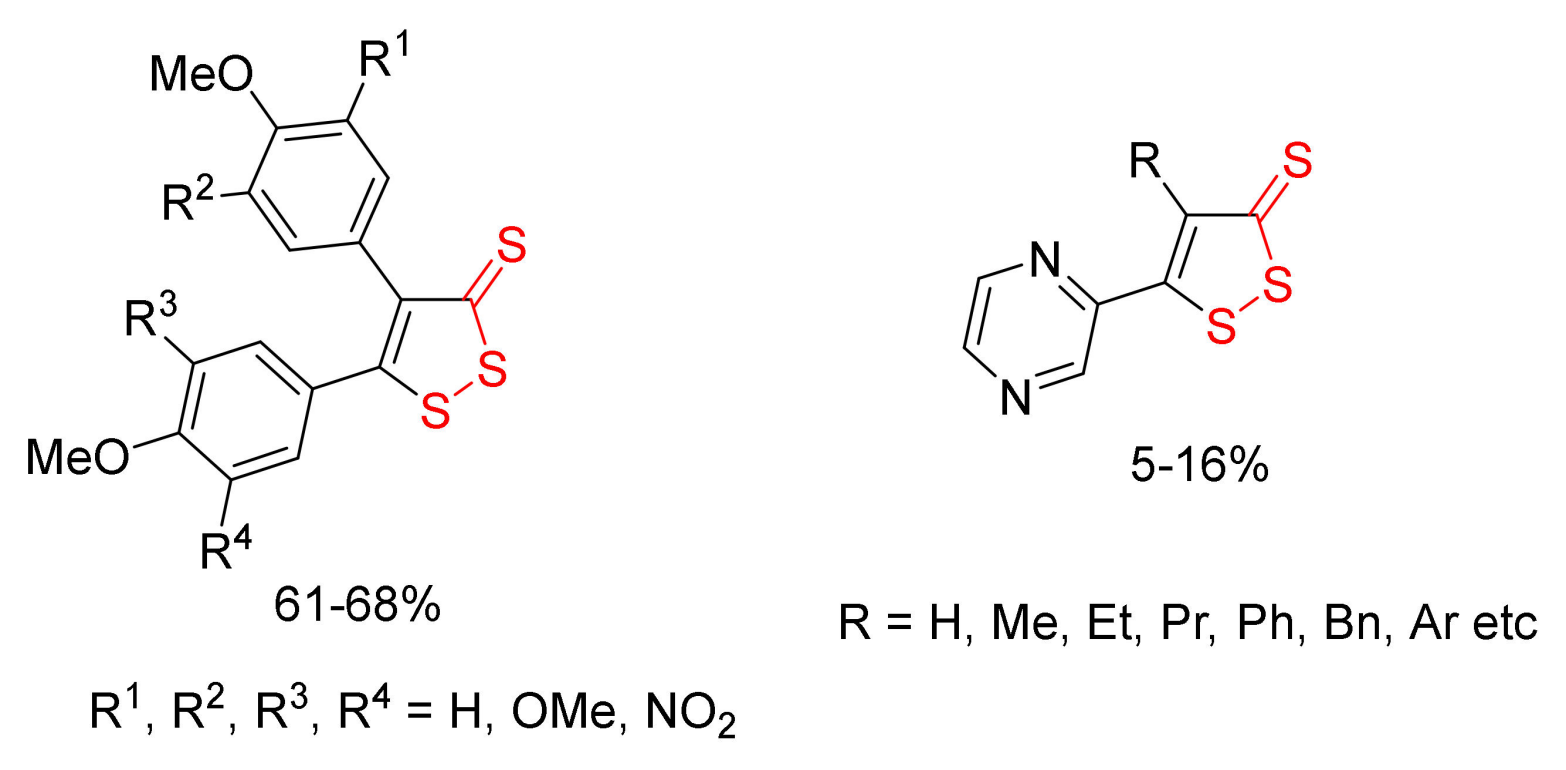
3*H*-1,2-Dithiole-3-thiones obtained by the reaction of 3-oxoesters and P_4_S_10_.

More recently it was shown that in some cases, for example, for 3-oxoesters containing a pyrazinyl group at C-3, this procedure can result in lower yields of 3*H*-1,2-dithiole-3-thiones, up to 39% [28]. Therefore, a number of attempts were made to modify this method, i.e., to replace Lawesson’s reagent with cheaper P_4_S_10_ and to avoid the use of elemental sulfur that complicated the purification of the final heterocycles. It was found that 2-(aryl)-3-oxo-3-(aryl)propanoates reacted with P_4_S_10_ in toluene under reflux conditions to give the corresponding 3*H*-1,2-dithiole-3-thiones in acceptable yields (Figure 2) [29]. Unfortunately, the use of this protocol for 3-oxo-3-(pyrazin-2-yl)propanoates still gave pyrazinyldithiole-3-thiones in low yields (5–16%) [30].

**Scheme 2:**
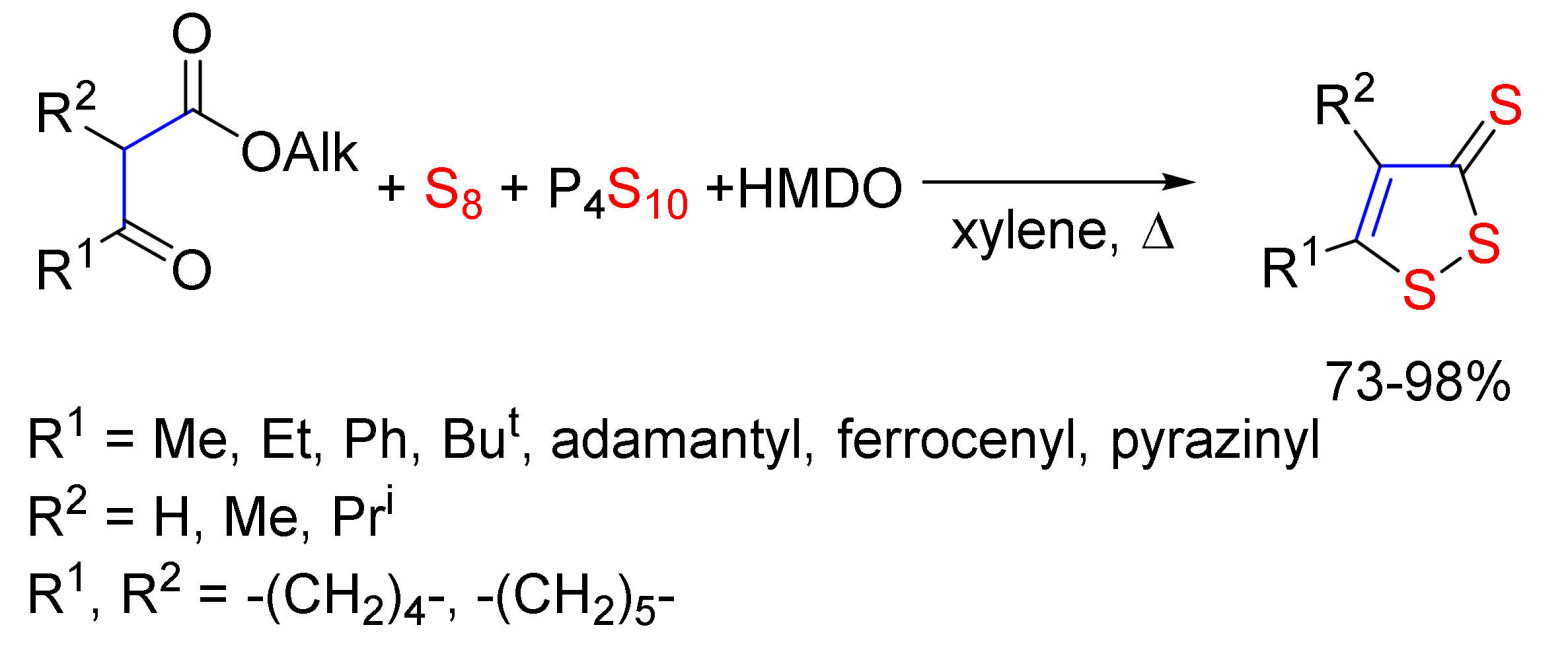
Synthesis of 3*H*-1,2-dithiole-3-thiones by the reaction of 3-oxoesters with sulfur, P_4_S_10_ and HMDO.

The most efficient procedure was developed by Curphey [31]. A combination of P_4_S_10_ and sulfur in the presence of hexamethyldisiloxane (HMDO) efficiently converted 3-oxoesters to dithiolethiones. In general, the yields of dithiolethiones obtained with P_4_S_10_/S_8_/HMDO mixtures were higher than those obtained with Lawesson’s reagent (Scheme 2). Addition of hexamethyldisiloxane (HMDO) to a P_4_S_10_-sulfur mixture both significantly increased the yield of dithiolethione **1** and greatly simplified the workup of the reaction mixture. Determination of the amount of HMDO remaining by the end of the reaction showed that about four equivalents of the disiloxane were consumed per one equivalent of P_4_S_10_. The role of HMDO can be explained as follows: in the presence of HMDO, highly electrophilic phosphorus species were converted to harmless silylated phosphates, thereby increasing the yield of the thionation product. On the other hand, removal of elemental sulfur from the reaction mixture reduced the yield of dithiolethiones, in agreement with the beneficial effects of sulfur observed in the conversion of 3-oxoesters to dithiolethiones by Lawesson’s reagent. The details of how sulfur acts to increase the yields of these dithiolethiones are not clear. Many other 3*H*-1,2-dithiole-3-thiones have been successfully prepared using this protocol [32].

### 2.2. Synthesis of 3H-1,2-dithiole-3-thiones from α-Enolic Dithioesters and Related Compounds

**Scheme 3:**
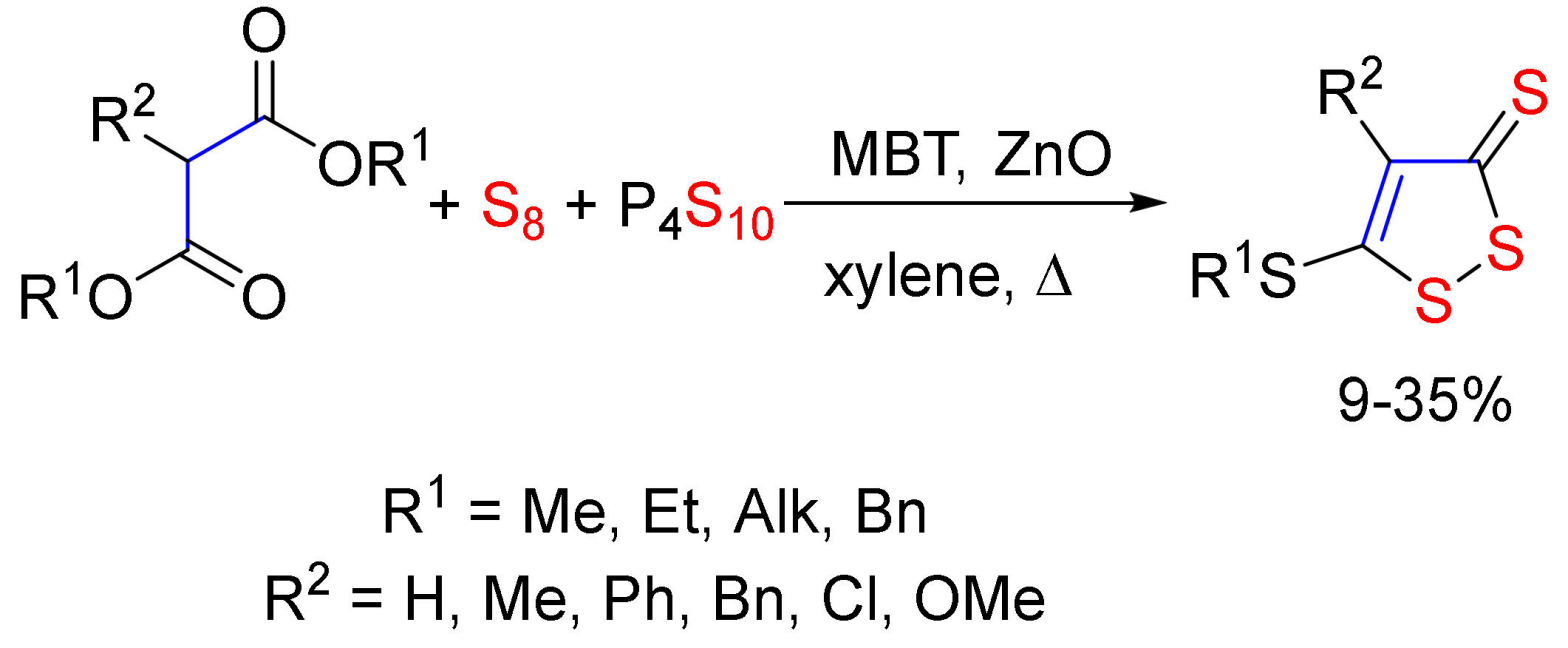
Synthesis of 4-substituted 5-alkylthio-3*H*-1,2-dithiole-3-thiones from dialkyl malonates.

Dialkyl malonates, α-enolic dithioesters or α-enolic dithioic acids can be successfully employed for the synthesis of various 3*H*-1,2-dithiole-3-thiones. Treatment of dialkyl malonates with a mixture of elemental sulfur and P_2_S_5_ in refluxing xylene resulted in 4-substituted 5-alkylthio-3*H*-1,2-dithiole-3-thiones as the major products [33,34]. The presence of a 2-mercaptobenzothiazole/ZnO mixture as the catalyst is essential for the reaction to occur successfully (Scheme 3). The result strongly depended on the structure of malonate esters. Malonate esters of primary alcohols gave moderate yields of dithiolethiones, while malonate esters of secondary alcohols did not. While dialkyl malonates containing Me, Ph, Bn, OMe and Cl substituents at position 2 successfully withstood the reaction conditions, 2-bromo- and 2-nitro-derivatives did not give the desired products.

**Scheme 4:**
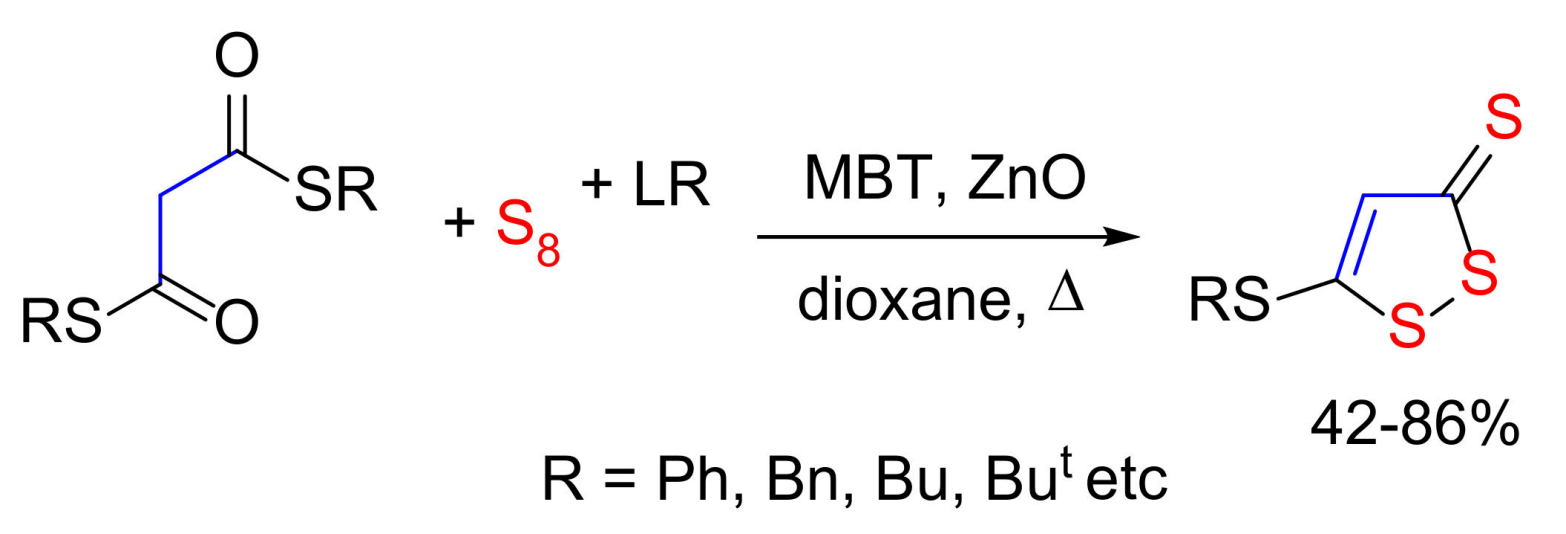
Synthesis of 3*H*-1,2-dithiole-3-thiones from dithiolmalonic esters.

If dithiolmalonic esters obtained from malonyl dichloride and the corresponding thiols were involved in the reaction with P_4_S_10_, 5-alkylthio-3*H*-1,2-dithiole-3-thiones were isolated from the reaction mixtures [35]. It was found that the use of Lawesson’s reagent as the sulfurating agent resulted in better yields of dithiolethiones (Scheme 4). A 2-mercaptobenzothiazole/ZnO mixture was successfully employed as the catalyst in these reactions.

**Scheme 5:**
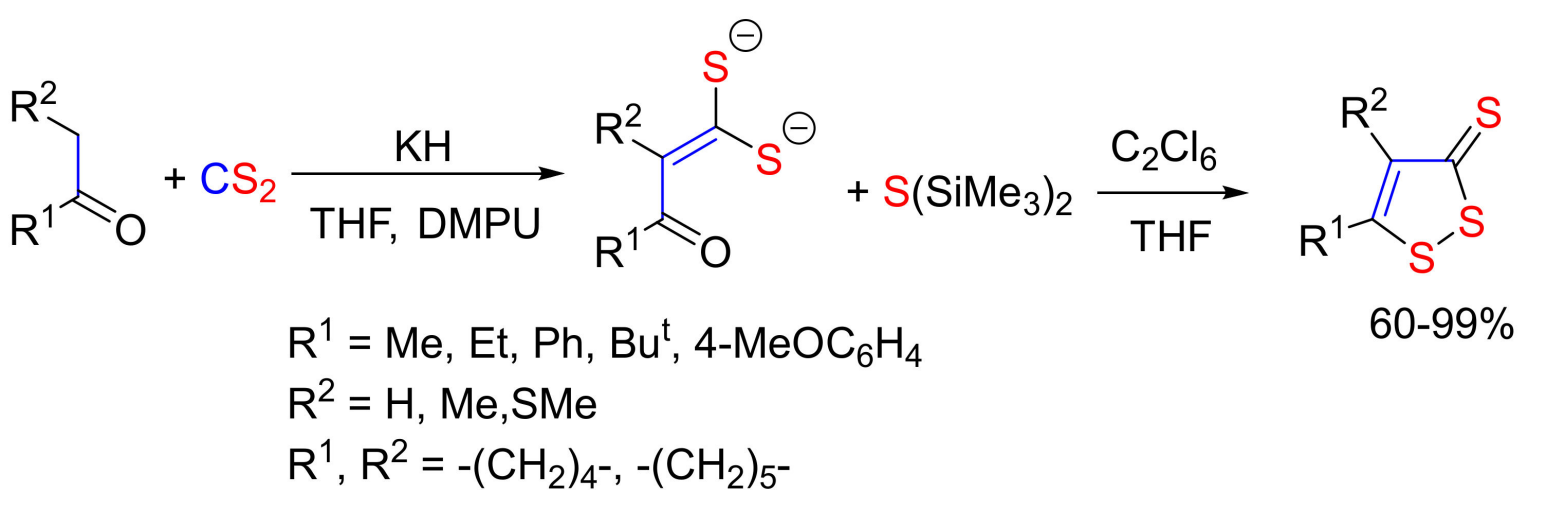
Synthesis of 3*H*-1,2-dithiole-3-thiones from dianions of 3-oxodithioic acids.

Yet another attractive approach to 1,2-dithiole-3-thiones based on various ketones via dianions of 3-oxodithioic acids was suggested by Curpey [36]. It was shown that the reaction of ketones with CS_2_ and two equivalents of KH in THF–*N*,*N*’-dimethylpropyleneurea (DMPU) solutions resulted in dianions of 3-oxodithioic acids. Sequential treatment of these dianions with hexamethyldisilathiane and hexachloroethane as the oxidizing agent gave 4,5-disubstituted 1,2-dithiole-3-thiones in good to excellent yields (Scheme 5). The use of a strong base such as KH and a dipolar aprotic cosolvent, either HMPA or DMPU, is necessary to convert the monoanion formed initially into a dianion. Other oxidizing agents such as bromine or iodine gave similar or slightly lower yields of 1,2-dithiole-3-thiones.

**Scheme 6:**
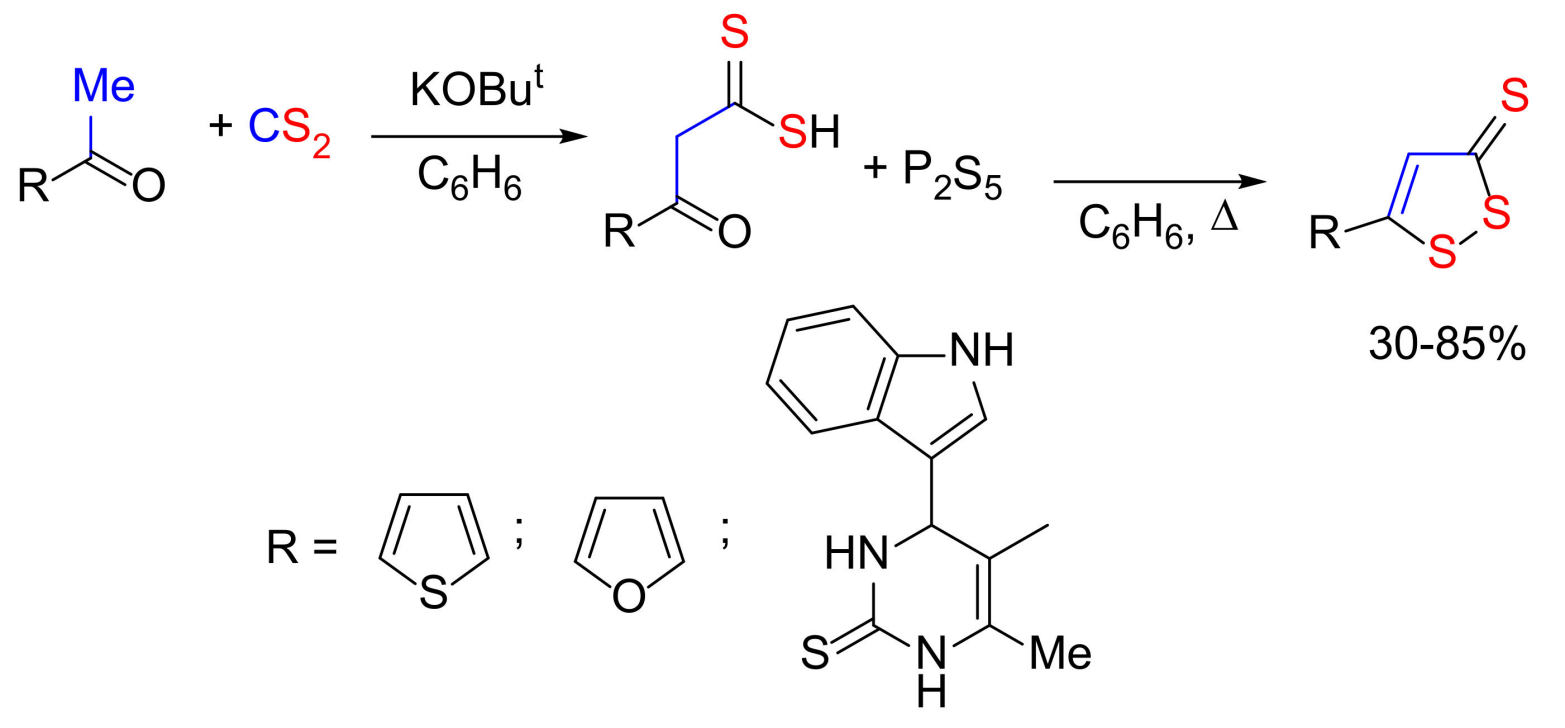
Synthesis of 5-substituted 3*H*-1,2-dithiole-3-thiones.

Although this is a general procedure, the use of expensive reagents such as KH and hexamethyldisilathiane greatly diminishes its usefulness. Later on, it was shown that for some heterocyclic acetyl derivatives KH can be replaced by potassium *tert*-butoxide and hexamethyldisilathiane by P_2_S_5_ (Scheme 6). In these cases, the yields may vary from good to low [37,38]. Unfortunately, it is still unclear whether this method is applicable to other ketones.

**Scheme 7:**
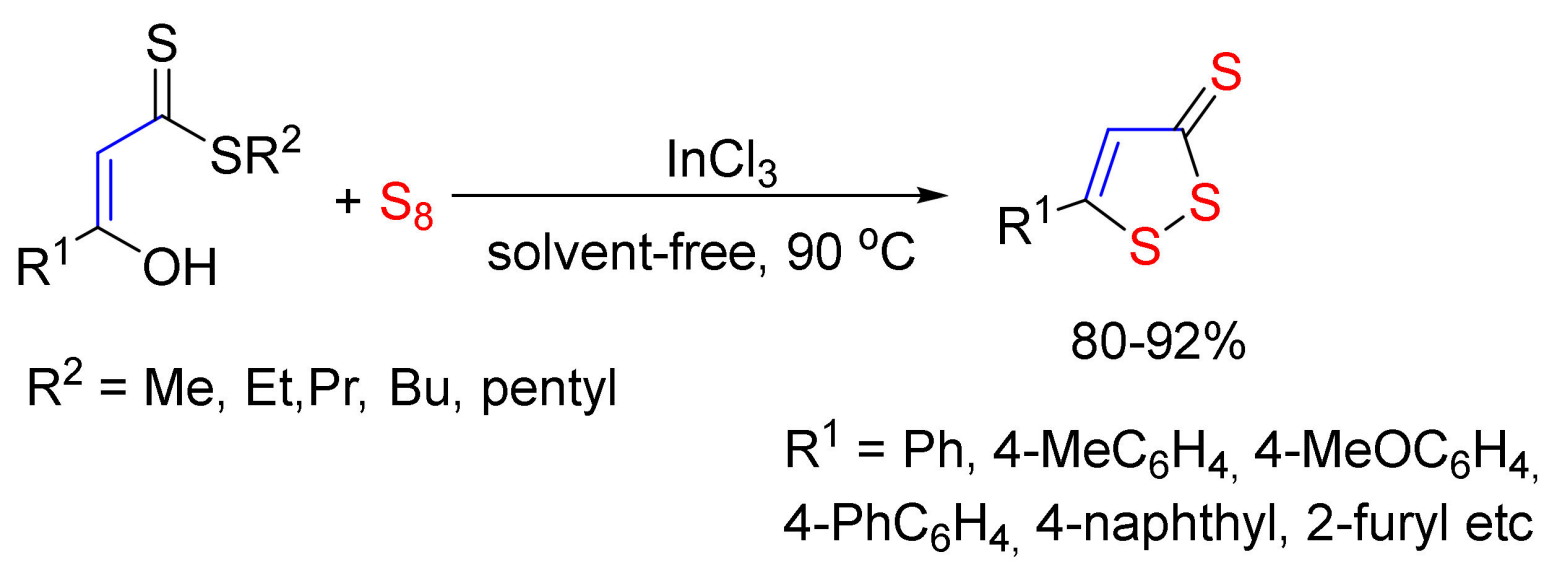
Synthesis of 5-substituted 3*H*-1,2-dithiole-3-thiones from α-enolic dithioesters.

5-Substituted 1,2-dithiole-3-thiones could also be obtained from α-enolic dithioesters [39]. Treatment of α-enolic dithioesters with easy-to-use reagents, namely, elemental sulfur and InCl_3_, at 90 °C under solvent-free conditions in air gave 3*H*-1,2-dithiole-3-thiones in good to excellent yields and showed good functional group tolerance to both electron-donating and electron-withdrawing groups (Scheme 7). It was found that various substituents such as OMe, Me, Cl, Br, and CF_3_ groups at *ortho*-, *meta*-, and *para*-positions of the phenyl ring were tolerated. This one-pot procedure involves the formation of new S−S and C−S bonds with in situ open-chain intermediates followed by intramolecular heterocyclization.

**Scheme 8:**
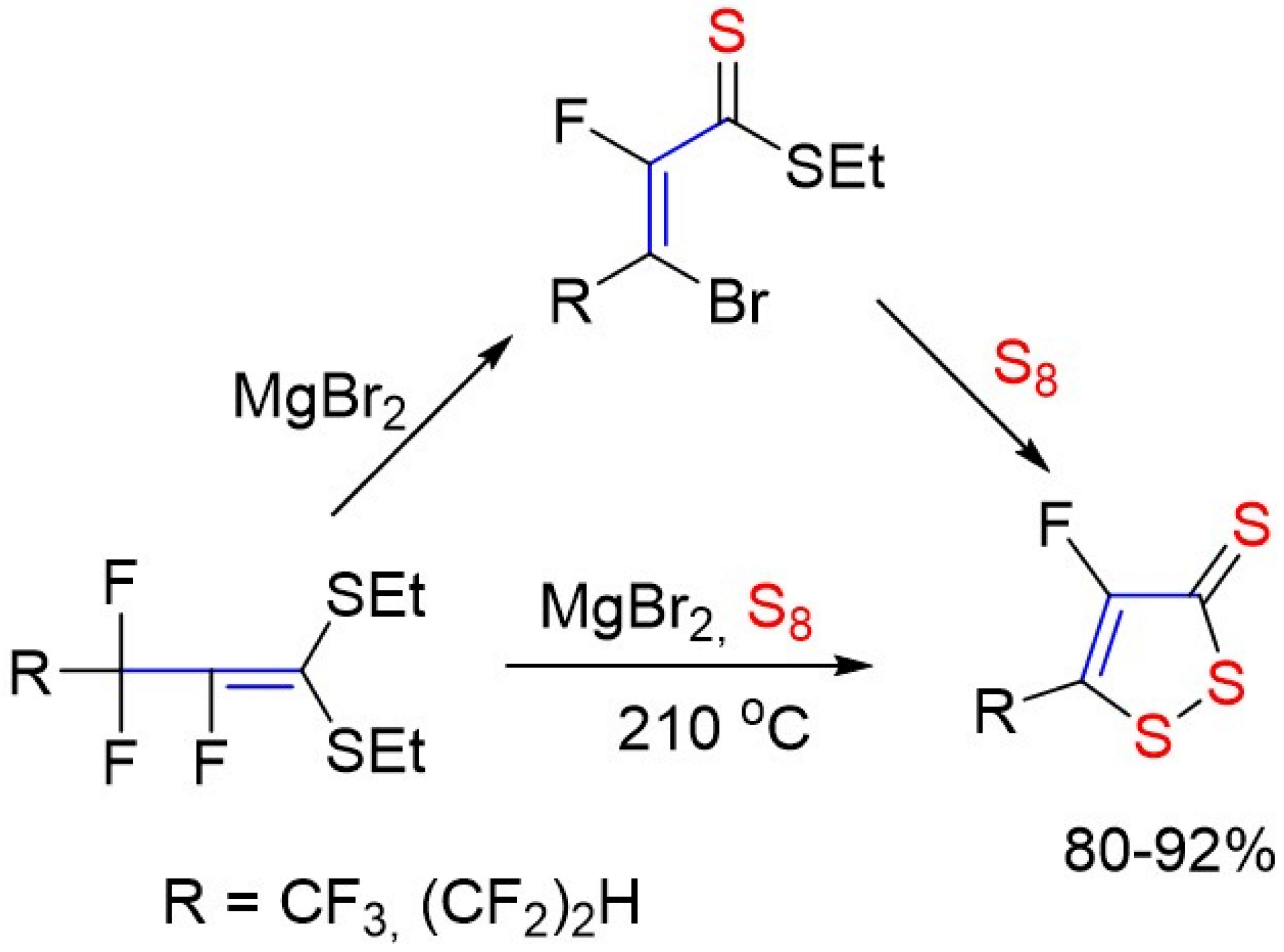
Synthesis of 4-fluoro-5-perfluoroalkyl-3*H*-1,2-dithiole-3-thiones.

4-Fluoro-5-perfluoroalkyl-3*H*-1,2-dithiole-3-thiones were synthesized in a one-pot procedure by heating the corresponding ketene dithioacetals with magnesium bromide and elemental sulfur at 210 °C (Scheme 8) [40]. An intermediate product in this reaction is β-bromo-β-trifluoromethyl dithiocrotonic ester that was isolated in the reaction of ketene dithioacetals with MgBr_2_.

### 2.3. Synthesis of 3H-1,2-dithiole-3-thiones from Iso-Propenyl Derivatives

Dehydrogenation and sulfuration of an *iso*-propenyl or *iso*-propyl group with phosphorus pentasulfide or elemental sulfur is the most awaited method that has been used for a long time [41]. However, it has the disadvantage of drastic reaction conditions (heating up to 200 °C) and has been rarely used lately.

**Scheme 9:**
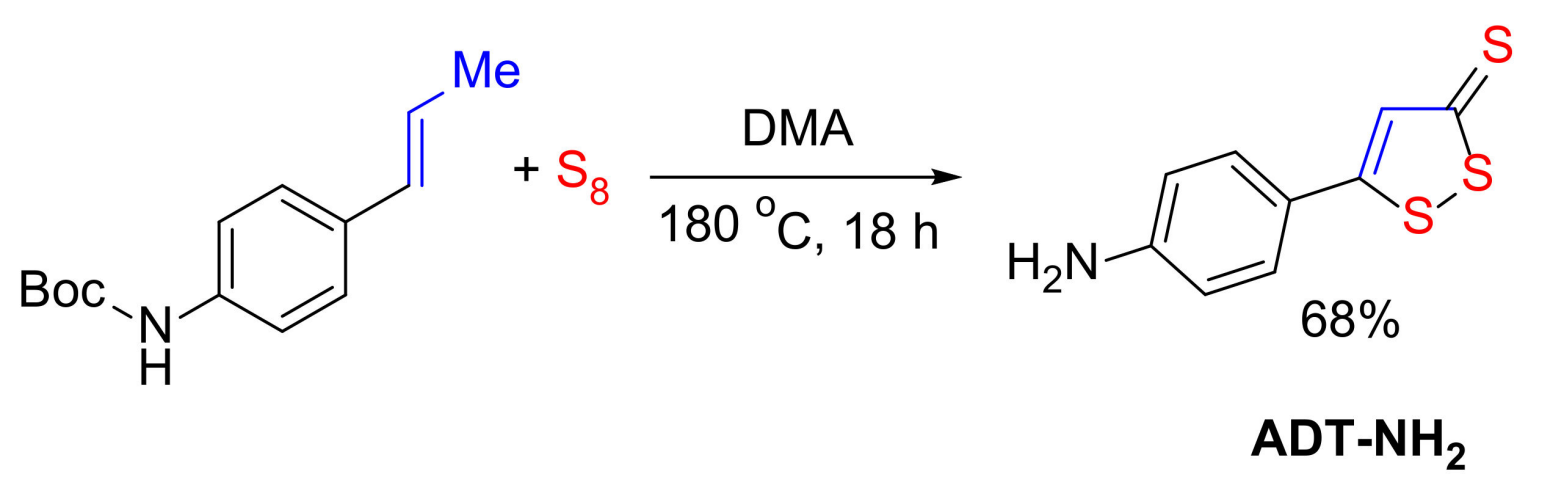
Synthesis of 5-(4-aminophenyl)-3*H*-1,2-dithiole-3-thione (**ADT-NH_2_**).

5-(4-Aminophenyl)-3*H*-1,2-dithiol-3-thione (amino-ADT, **ADT-NH_2_**) can be prepared by treatment of *tert*-butyl (*E*)-(4-(prop-1-en-1-yl)phenyl)carbamate with elemental sulfur at 180 °C with simultaneous formation of dithiolethione and deprotection of aniline to give **ADT-NH_2_** (Scheme 9) [42].

**Scheme 10:**
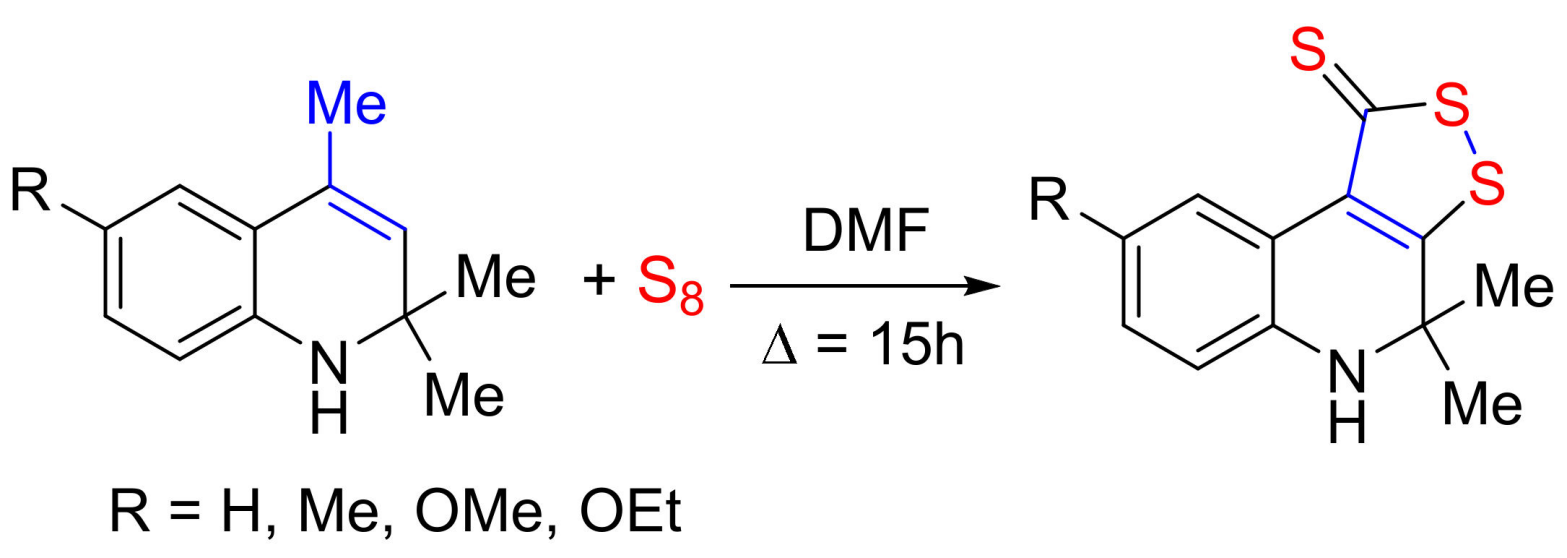
Synthesis of 4,4-dimethyl-4,5-dihydro-1*H*-[1,2]dithiolo[3,4-*c*]quinoline-1-thiones.

4,4-Dimethyl-4,5-dihydro-1*H*-[1,2]dithiolo[3,4-*c*]quinoline-1-thiones were synthesized by refluxing dihydroquinolines containing a hidden *iso*-propenyl group in the ring in dimethylformamide with a 5-fold excess of elemental sulfur (Scheme 10) [43].

**Scheme 11:**
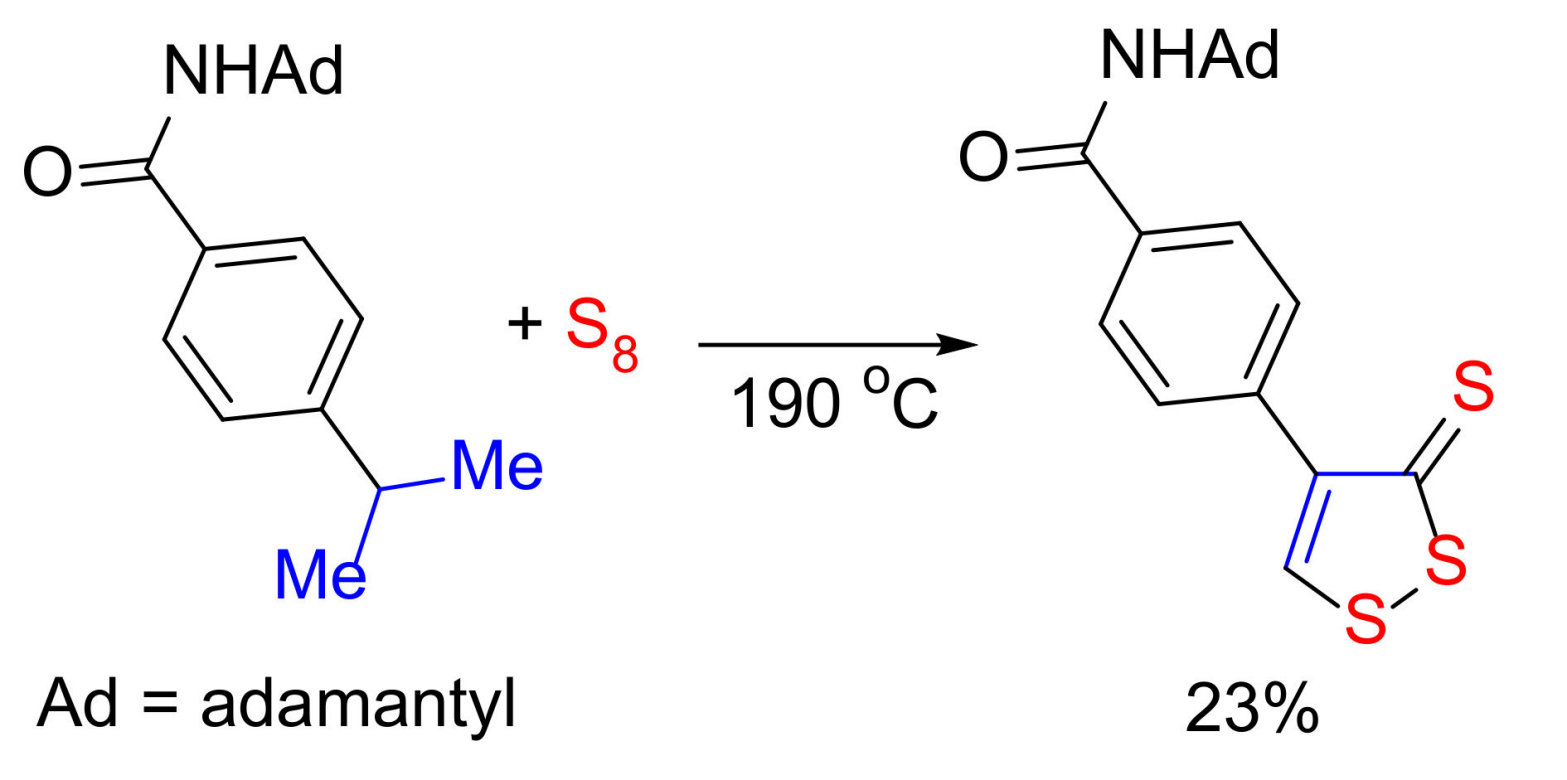
Synthesis of *N*-((3r)-adamantan-1-yl)-4-(3-thioxo-3*H*-1,2-dithiol-4-yl)benzamide.

Heating *N*-((3r)-adamantan-1-yl)-4-isopropylbenzamide with elemental sulfur at 190 °C overnight gave *N*-((3r)-adamantan-1-yl)-4-(3-thioxo-3*H*-1,2-dithiol-4-yl)benzamide in a low yield (Scheme 11) [44].

### 2.4. Synthesis of 3H-1,2-dithiole-3-thiones from Alkynes

**Scheme 12:**
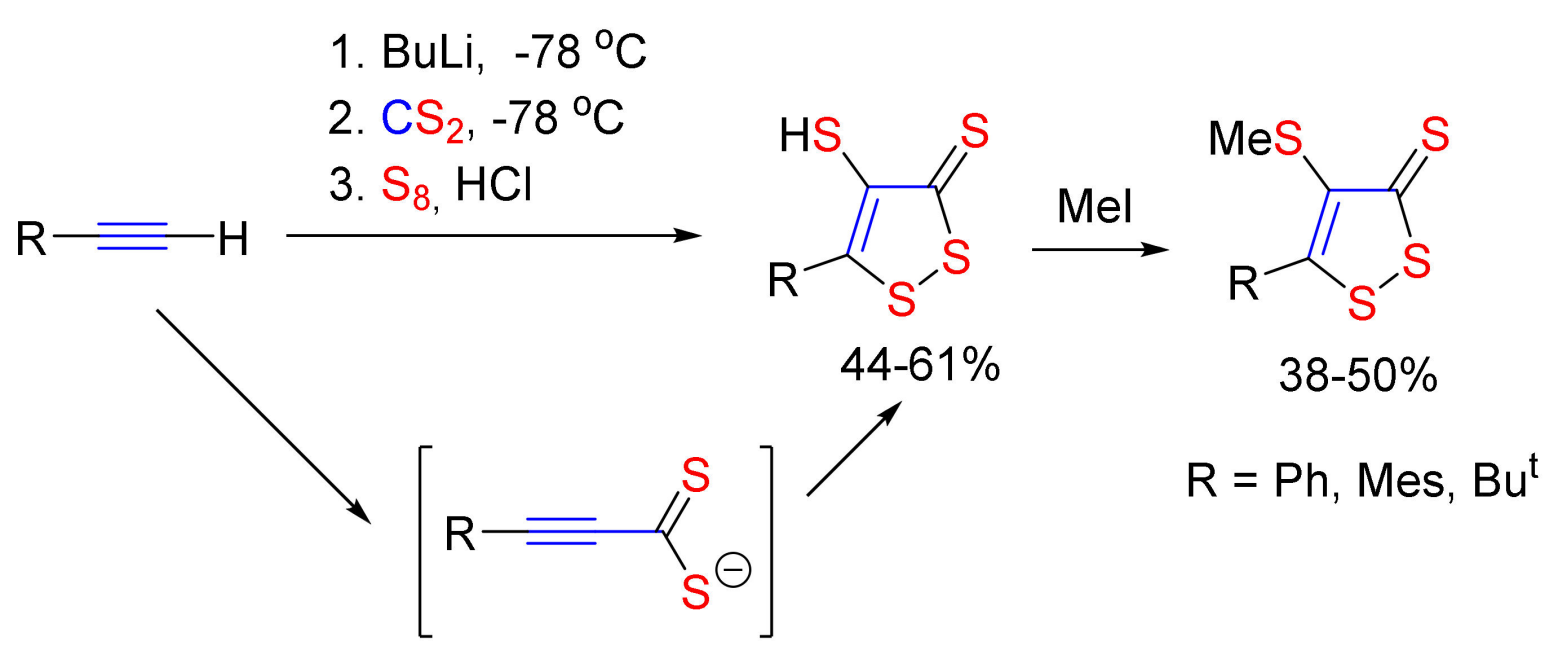
Synthesis of 4-mercapto-5-substituted 3*H*-1,2-dithiole-3-thiones from alkynes.

4-Mercapto-5-substituted 3*H*-1,2-dithiole-3-thiones can be easily prepared by a one-pot procedure from terminal alkynes [45]. Deprotonation of terminal alkynes with BuLi followed by treatment with carbon disulfide resulted in alkynyldithiocarboxylates, which were then treated with elemental sulfur to give 4-mercapto derivatives after acidic workup (Scheme 12). If the reaction mixtures were quenched with methyl iodide rather than HCl, stable 4-methylthio derivatives were isolated in similar yields.

**Scheme 13:**
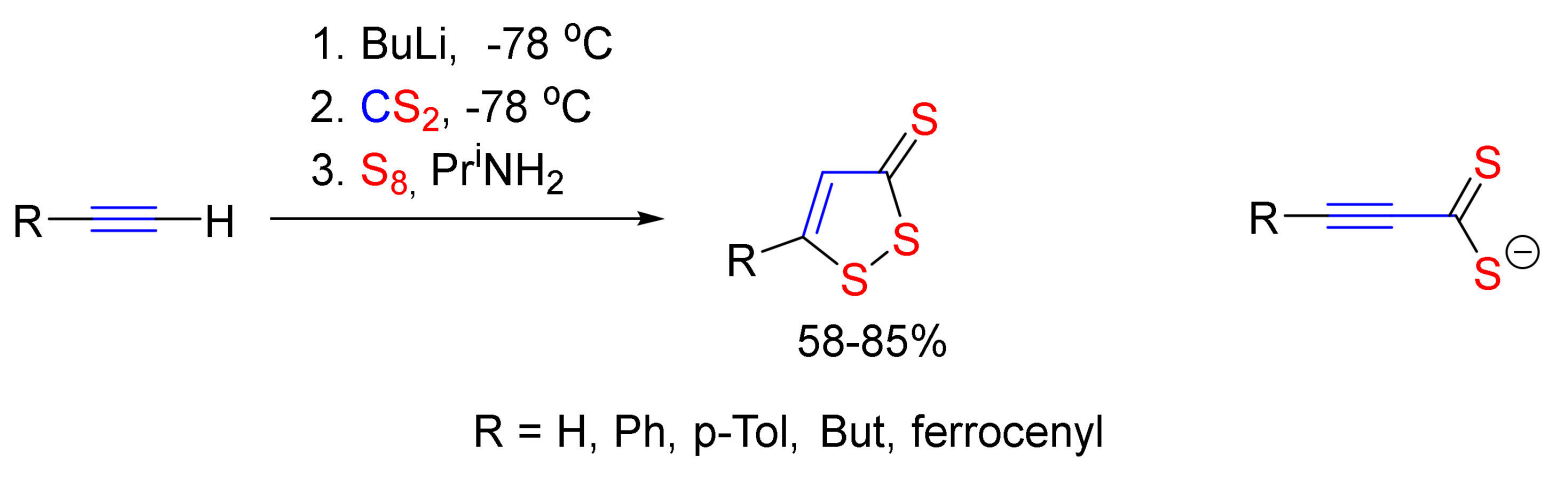
Synthesis of 5-substituted 3*H*-1,2-dithiole-3-thiones from alkynes.

Surprisingly, if isopropylamine is added to the reaction mixture instead of HCl, the reaction can be stopped with the formation of 5-substituted 1,2-dithiole-3-thiones [46]. The method is very convenient and a number of 5-substituted 1,2-dithiole-3-thiones were successfully synthesized (Scheme 13). Along with the formation of a heterocyclic ring, trimethylsilylacetylene underwent desilylation in the reaction to give the parent heterocycle in a moderate yield.

**Scheme 14:**
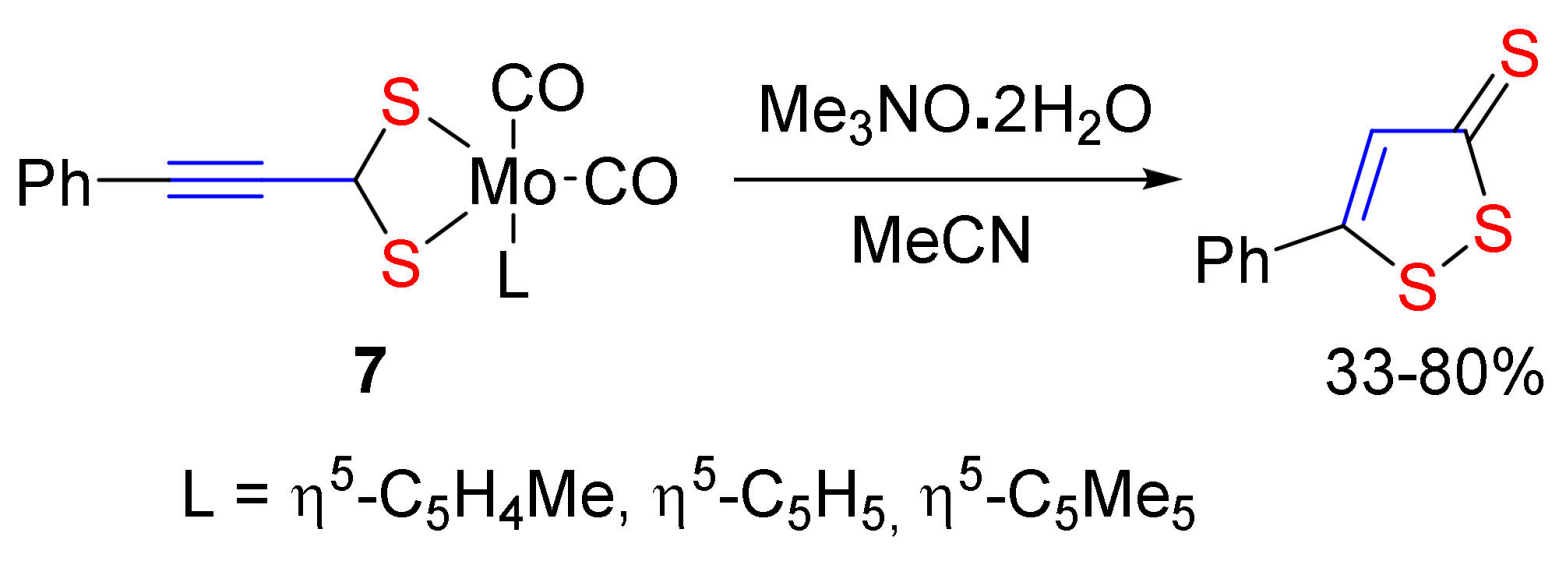
Synthesis of 5-phenyl-3*H*-1,2-dithiole-3-thione from molybdenum dithiopropiolato complexes.

5-Phenyl-3*H*-1,2-dithiole-3-thione was prepared from molybdenum dithiopropiolato complexes **7** [47]. Treatment of these complexes with trimethylamine-*N*-oxide in MeCN resulted in 1,2-dithiole-3-thione (Scheme 14). The authors assumed the formation of intermediate molybdenum oxo-complexes, which were isolated after the first stage and then subjected to hydration with water from Me_3_NO∙H_2_O (the use of anhydrous TMNO did not give dithiolethione). The authors did not explain where the third sulfur atom of dithiolethione comes from, and even more surprisingly, addition of elemental sulfur did not improve the yields of 5-phenyl-3*H*-1,2-dithiole-3-thione.

**Scheme 15:**
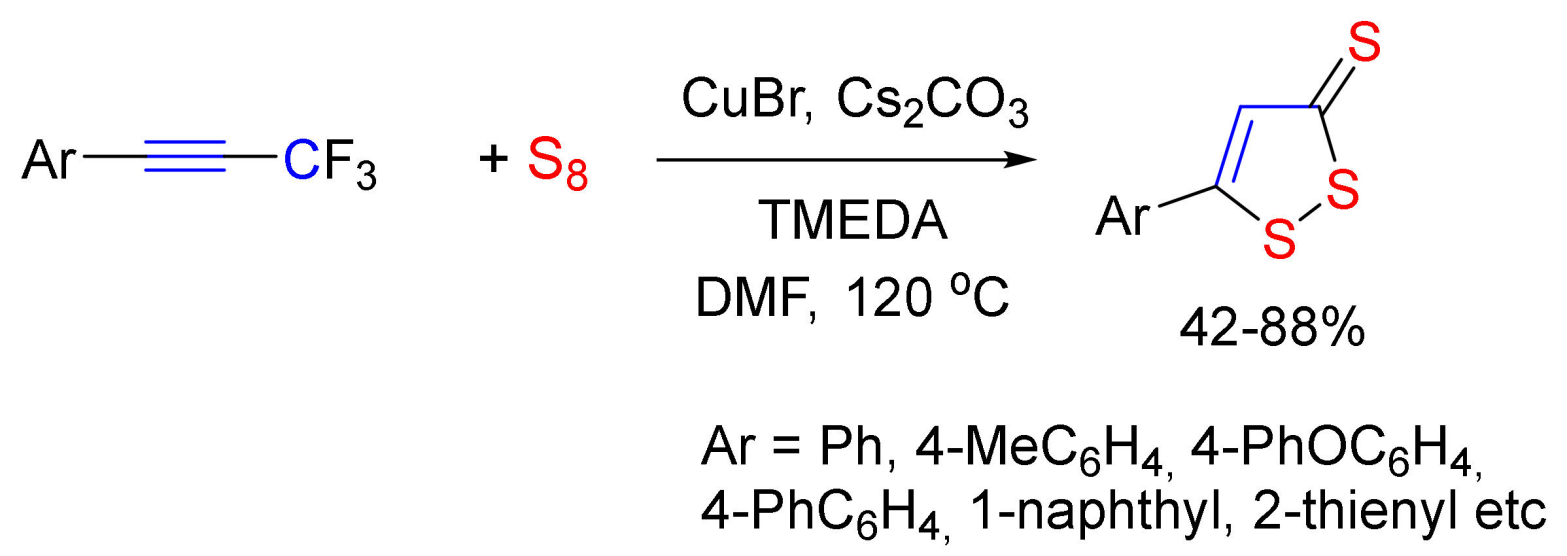
Synthesis of 5-aryl-3*H*-1,2-dithiole-3-thiones from trifluoropropynes.

Zhang’s team recently published two efficient syntheses of monosubstituted 1,2-dithiole-3-thiones from internal alkynes. Copper catalyzed defluorinating thioannulation of aryl trifluoropropynes using elemental sulfur as the only sulfur source proved to be a simple and practical strategy for the preparation of 5-aryl-3*H*-1,2-dithiole-3-thiones (Scheme 15) [48]. Specific features of this reaction include the efficient formation of multiple C-S bonds due to cleavage of C-F bonds in the CF_3_ group, a wide scope of substrates, and a high tolerance to functional groups; 1,2-dithiole-3-thiones are formed in moderate to high yields on treatment of trifluoropropynes with S_8_ and Cs_2_CO_3_ in the presence of CuBr and TMEDA in DMF at 120 °C.

**Scheme 16:**
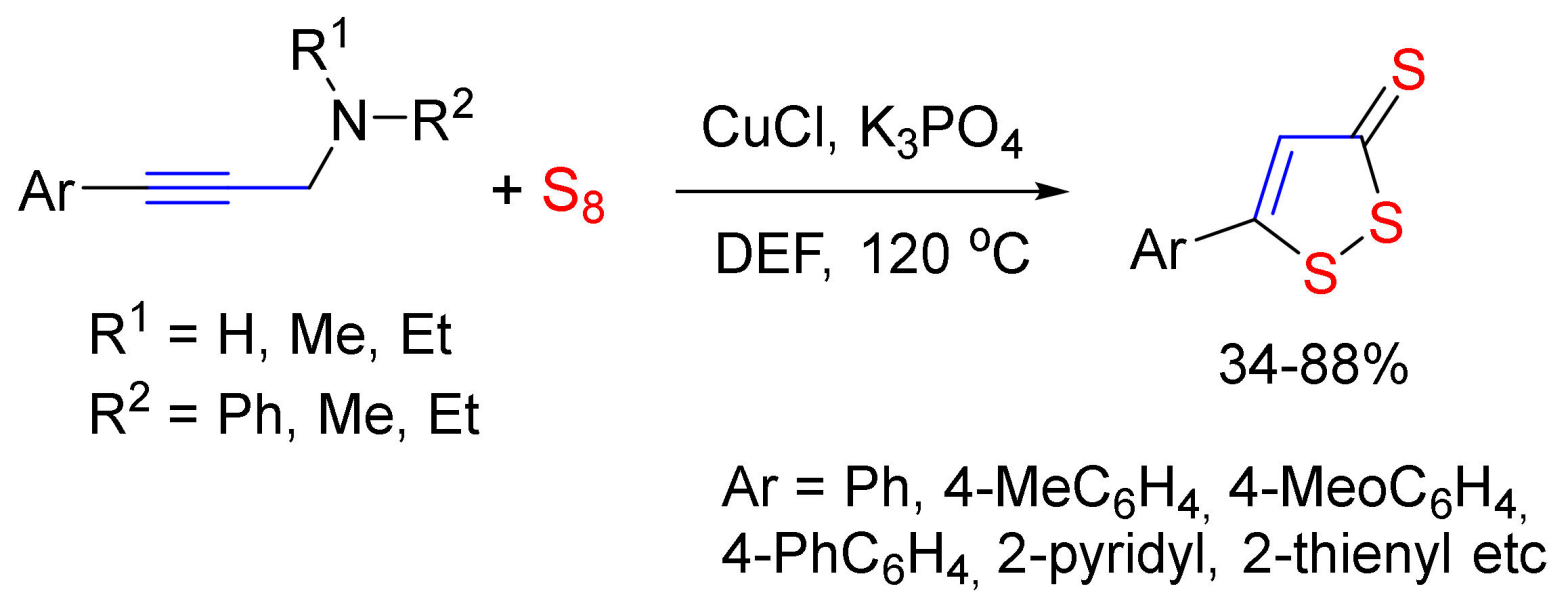
Synthesis of 5-aryl-3*H*-1,2-dithiole-3-thiones from propargylamines.

Yet another approach involves the copper-catalyzed aerobic oxidative sulfuration and annulation of propargylamines with elemental sulfur in diethylformamide (DEF) [49]. The tandem reaction includes the cleavage of the C−N bond and formation of multiple C−S bonds to give 5-aryl-3*H*-1,2-dithiole-3-thiones in good to excellent yields with perfect electron-rich and electron-poor aryl (hetaryl) group tolerance (Scheme 16). Various secondary and tertiary *N*-phenylpropargyl amines can be involved in this reaction. A possible mechanism includes the copper-catalyzed oxidative dehydrogenation of propargylamines in air followed by disproportionation of elemental sulfur in the presence of K_3_PO_4_ with release of the S_2_ dianion and the SH anion. Simultaneous nucleophilic additions of the S_2_ dianion to the carbon-carbon triple bond followed by copper-catalyzed dehydrogenative oxidation and hydrolysis resulted in 1,2-dithiol-3-one. Finally, nucleophilic addition of the SH anion to 1,2-dithiol-3-one followed by elimination gave the target 1,2-dithiole-3-thione.

### 2.5. Synthesis of 3H-1,2-dithiole-3-thiones from Tertiary Isopropylamines and Disulfur Dichloride

The general strategy for the synthesis of 1,2-dithioles from isopropyl or isopropenyl derivatives is to add two sulfur atoms from elemental sulfur. Recently, however, disulfur dichloride has successfully replaced this reagent for tertiary isopropylamines. The main feature of this reagent is that it has diverse reactivity, which determines both its beneficial and adverse properties. Disulfur dichloride exhibits the properties of a sulfurating, chlorinating, oxidizing, and even dehydrating agent [50]. The most important property of disulfur dichloride is its ability to cause cyclization of various organic molecules into sulfur-containing heterocycles, in particular 1,2-dithiole-3-thiones [51,52,53].

**Scheme 17:**
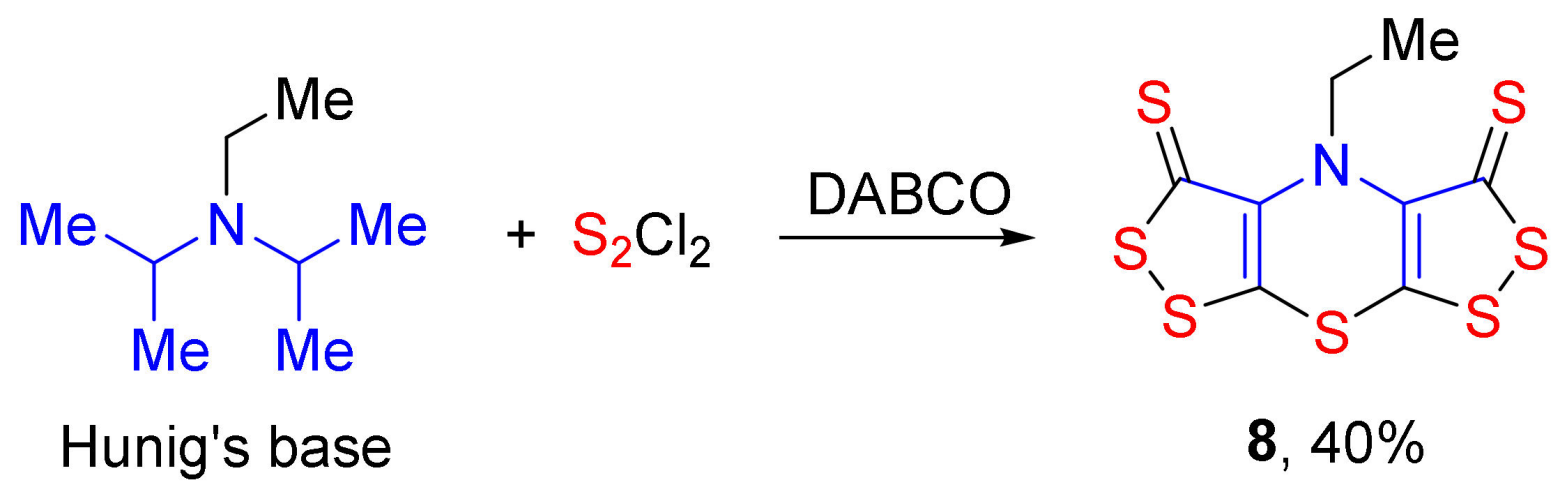
Reaction of Hünig’s base with disulfur dichloride.

The concept of synthesizing 1,2-dithioles from tertiary isopropylamines and disulfur dichloride was discovered and developed by the author of this review in collaboration with Professor Rees (Imperial College London, UK) in the late 90s of the past century and at the beginning of this century. It was found that *N*-ethyldiisopropylamine (Hünig’s base), which was previously used in reactions with disulfur dichloride as an “inert” base, can react with S_2_Cl_2_ and 1,4-diazabicyclooctane (DABCO) to give a completely unexpected new polysulfur-containing heterocyclic system, namely, bis[1,2]dithiolo[1,4]thiazine **8** [54]. In this one-pot reaction, the 14 isopropyl C-H bonds of the Hünig base were replaced by 10 C-S and two C= C bonds, while the ethyl group was left intact (Scheme 17).

**Scheme 18:**
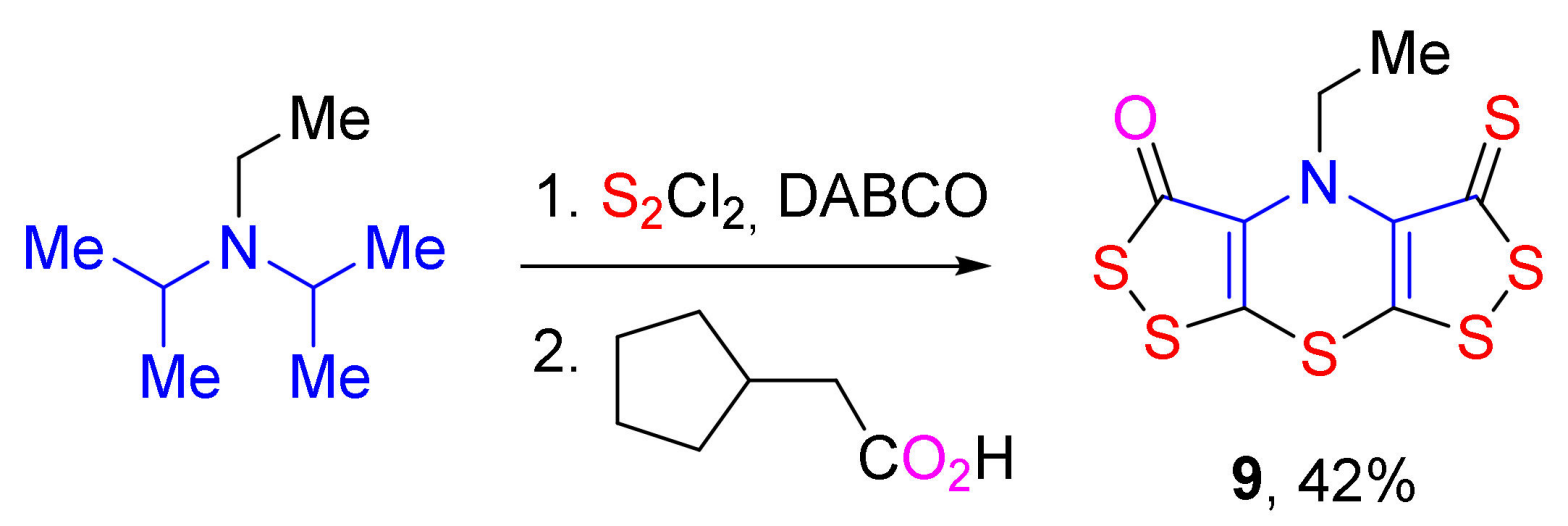
Synthesis of 4-ethyl-5-thioxo-3*H*,4*H*,5*H*-bis([1,2]dithiolo)[3,4-*b*:4’,3’-*e*][1,4]thiazin-3-one.

4-Ethyl-5-thioxo-3*H*,4*H*,5*H*-bis([1,2]dithiolo)[3,4-*b*:4’,3’-*e*][1,4]thiazin-3-one **9** was obtained by the reaction of Hünig’s base with disulfur dichloride with addition of an oxygen donor, namely cyclopentylacetic acid, at the last stage of the reaction (Scheme 18) [55]. By extending the conditions found to other substituted diisopropylamines, a number of bis(dithiolo)thiazines were obtained, including nitrogen-unsubstituted representatives of these heterocycles [56,57,58].

**Scheme 19:**
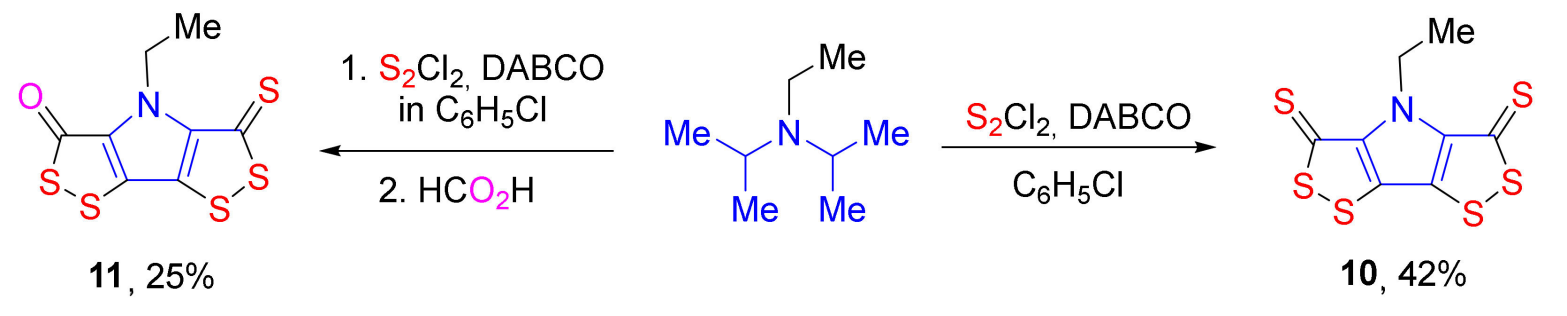
Synthesis of bis[1,2]dithiolopyrroles.

If the reaction was carried out in a high boiling solvent such as chlorobenzene, bis[1,2]dithiolopyrroles **10** and **11** were formed by elimination of a sulfur atom from the intermediate bis(dithiolo)thiazines **8** and **9** (Scheme 19) [59].

**Scheme 20:**
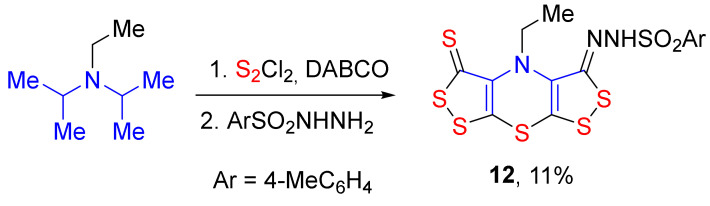
Synthesis of monohydrazide **12**.

The reaction of Hünig’s base, disulfur dichloride, and *p*-toluenesulfonic acid hydrazide under similar conditions occurred in a more complex way to give monohydrazide **12** in a low yield (Scheme 20) [60].

**Scheme 21:**
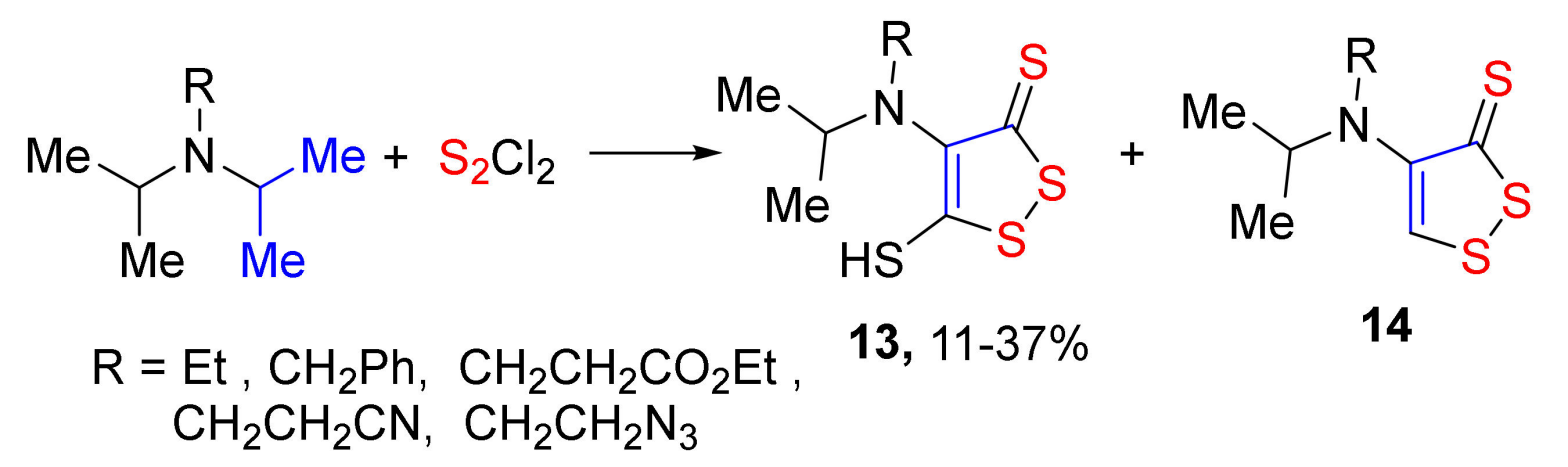
Conversion of *N*-alkyldiisopropylamines into monocyclic 1,2-dithioles.

In all the reactions described above in this section, both isopropyl groups in the *N*-alkyldiisopropylamines were converted to 1,2-dithiole rings. It was shown later that the reaction could be stopped at the stage of the formation of monocyclic 1,2-dithioles, and the main condition for the successful synthesis of monodithioles is that the conversion should be performed at low temperature [61]. Treatment of *N*-alkyldiisopropylamines and S_2_Cl_2_ in chloroform at 0 °C in the absence of another base resulted in monocyclic 1,2-dithiole-3-thiones **13** and **14**; in all cases, the 5-mercapto derivatives **13** were the main products (Scheme 21).

**Scheme 22:**
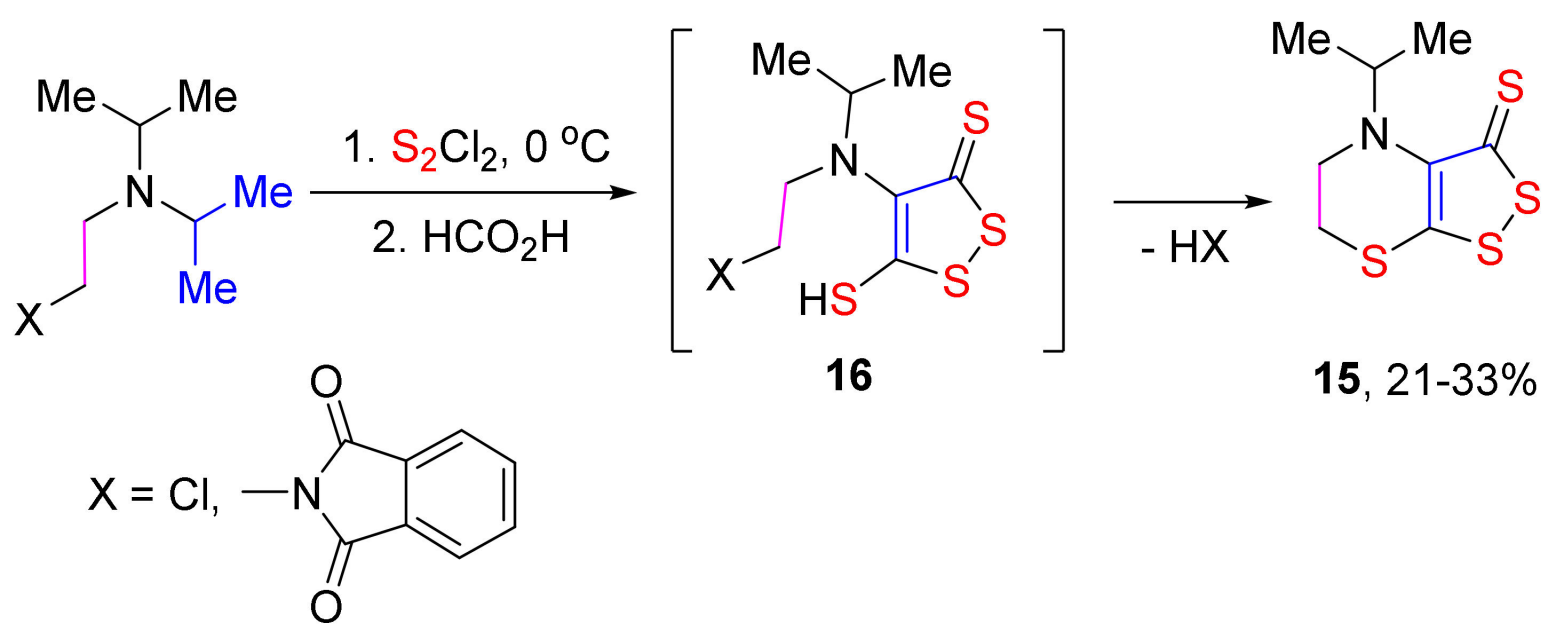
Synthesis of dithiolothiazine **15**.

Unexpected results were obtained in the reaction of two other substituted diisopropylamines with disulfur dichloride [62]. Dithiolothiazine **15** was isolated instead of the expected mercaptodithiolethiones **16** (Scheme 22). The authors believe that in the course of the reaction, thiones **16** were also formed as intermediates and then converted into bicyclic structure **15** after elimination of HCl or phthalimide, respectively.

**Scheme 23:**
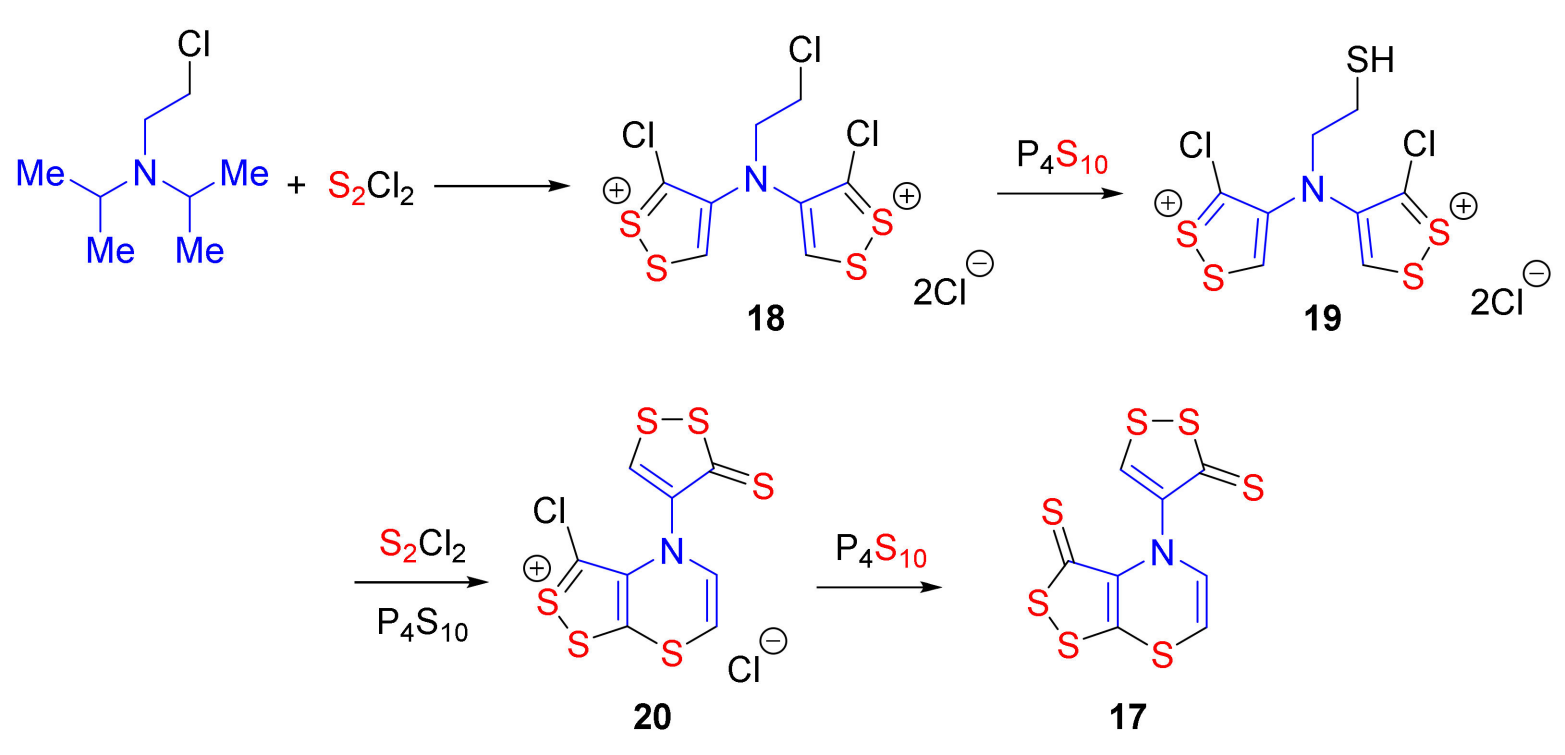
Reaction of *N*-(2-chloroethyl)diisopropylamine with disulfur dichloride and phosphorus pentasulfide.

Treatment of *N*-(2-chloroethyl) diisopropylamine with disulfur dichloride followed by addition of phosphorus pentasulfide resulted in dithiolothiazine **17** [63]. The formation of this product can be explained by the fact that, in the presence of P_4_S_10_, salt **18** gives another salt **19**, which undergoes cyclization to thiazine ring **20**. The conversion of salt **20** into thiazine **17** apparently occurs due to the action of the same P_4_S_10_ as a sulfurating agent (Scheme 23).

**Scheme 24:**
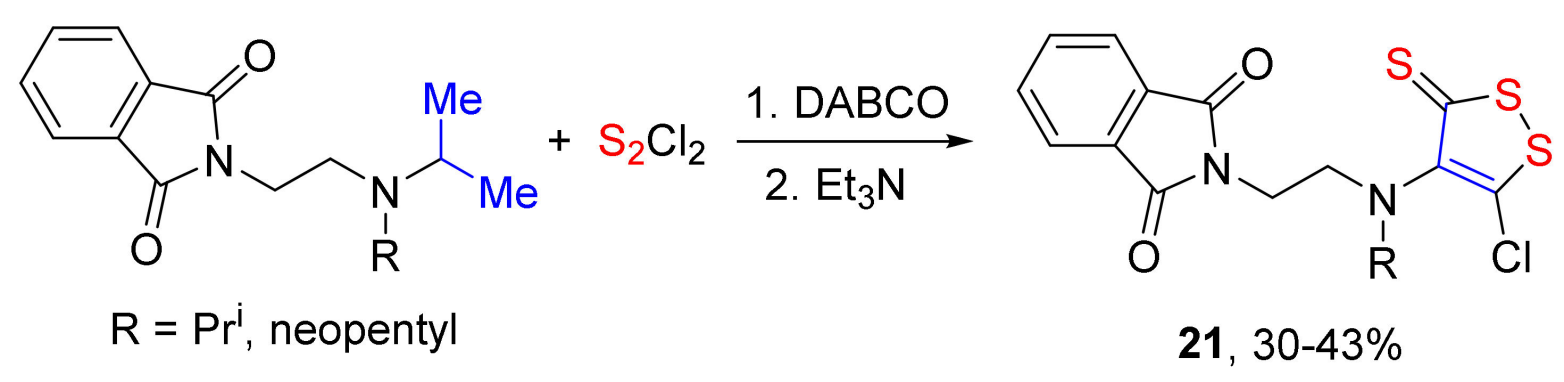
Reaction of *N*-(2-phthalimidoethyl)-*N*-alkylisopropylamines with disulfur dichloride.

5-Chloro-1,2-dithiole-3-thiones **21** were obtained by the reaction of *N*-(2-phthalimidoethyl)-*N*-alkylisopropylamines with a mixture of disulfur dichloride and DABCO followed by the action of triethylamine [64]. The unexpected stability of thiones **21** was explained by the dipole-dipole interaction between an electron-donor 1,2-dithiole-3-thione ring and an electron-withdrawing phthalimide group (Scheme 24).

**Scheme 25:**
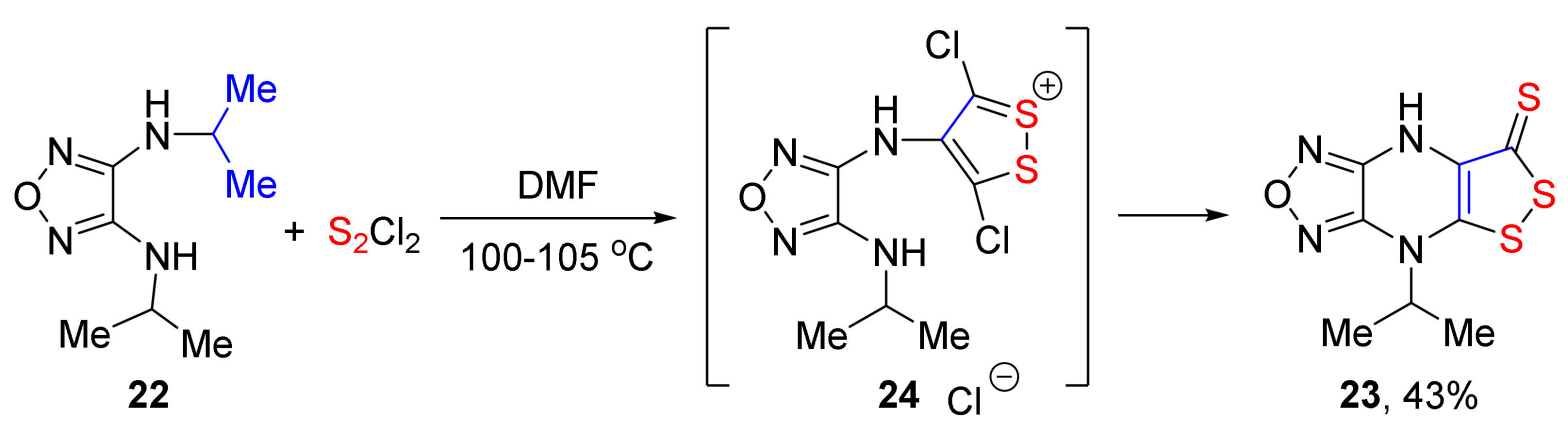
Reaction of 3,4-bis(*iso*-propylamino)-1,2,5-oxadiazole with disulfur dichloride.

Treatment of 3,4-bis(*iso*-propylamino)-1,2,5-oxadiazole **22** with disulfur dichloride in DMF at 100–105 °C gave a product containing one isopropyl group, dithioloxadiazolopyrazinethione **23**, whose structure was confirmed by X-ray diffraction analysis [65]. The formation of this product was explained by the conversion of the *N*-isopropyl group to the 3-chlorodithiolium salt **24** in accordance with the mechanism described earlier [55]. The latter compound evolved hydrogen chloride to form the pyrazine ring (Scheme 25). Obviously, the formation of the dithiolethione and pyrazine rings in compound **23** deactivates the second isopropyl group, which does not undergo further conversion.

**Scheme 26:**
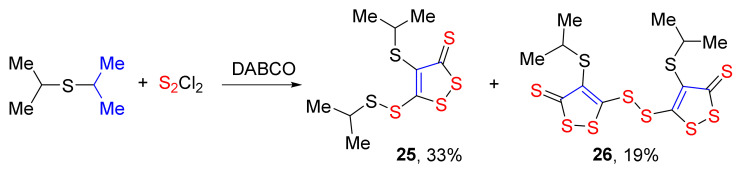
Reaction of diisopropyl sulfide with disulfur dichloride.

The reaction of diisopropyl sulfide with disulfur dichloride and DABCO resulted in 1,2-dithiole-3-thiones **25** and **26** [66]. Apparently, the formation of the dithiole ring is similar to its production from diisopropylamines. However, in the case of diisopropyl sulfide, only one isopropyl group reacted, whereas the second one was apparently deactivated by incorporation of a dithiolethione moiety at the sulfur atom (Scheme 26).

It was shown above that *N*-isopropyl groups could be converted to *N*-(1,2-dithiole-3-thione) moieties. Nitrogen-containing heterocycles with methyl and C-H groups at the *ortho*-position, for example, readily available 2- and 3-methylindoles, are structurally similar to the *N*-isopropyl group and can be considered as starting materials for the synthesis of dithioloindoles.

**Scheme 27:**
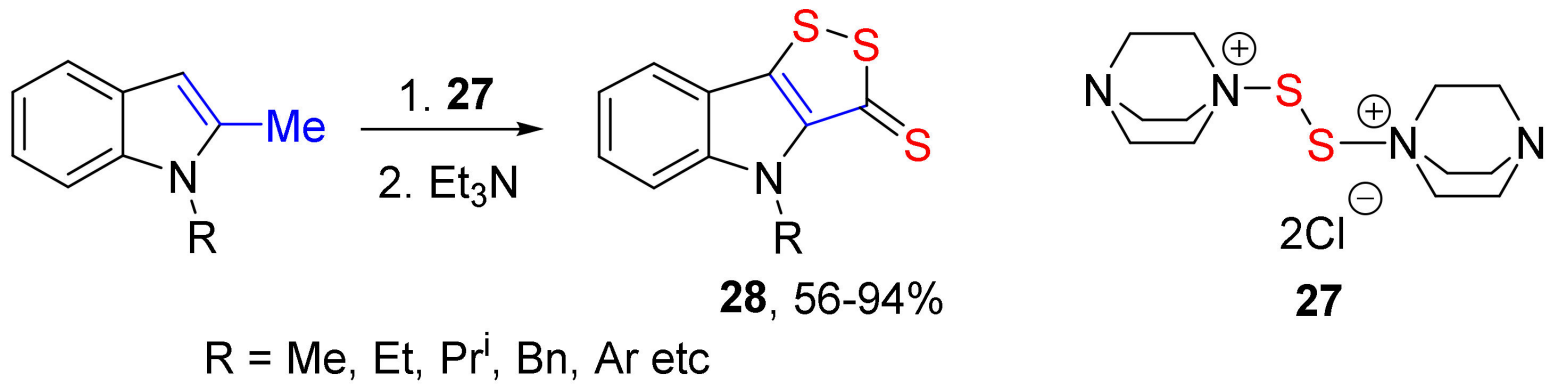
Synthesis of dithioloindoles **28**.

Treatment of *N*-methyl-2-methylindole with a fivefold excess of disalt **27** obtained from disulfur dichloride (one equivalent) and DABCO (two equivalents) in chloroform at room temperature [67] followed by addition of triethylamine gave fused dithioloindole **28** (R = Me) in high to moderate yields [68]. Later, this reaction was extended to other *N*-substituted 2-methylindoles [69]. Fused dithioloindoles **28** were obtained in good yields (Scheme 27). *N*-Acetyl- and *N*-benzoyl-2-methylindoles did not react with S_2_Cl_2_ even under more drastic conditions; the starting indoles were isolated from the reaction mixtures in high yields. It is obvious that electron-withdrawing substituents at the nitrogen atom of the indole ring (acetyl and benzoyl groups) suppress the reaction with electrophilic disulfur dichloride. It was found that the 3-methyl group in 1,3-dimethylindole does not react with S_2_Cl_2_ and its mixtures, in contrast to the 2-methyl group in 1,2-dimethylindole. The high reactivity of the 2-methyl group can be explained by the low acidity of the 2-methyl hydrogens. The most plausible mechanism for the formation of dithioloindoles is apparently similar to the mechanism of the formation of a 1,2-dithiole-3-thione moiety from tertiary *N*-isopropylamines and involves the addition of a S_2_Cl_2_ molecule followed by the formation of a 1,2-dithiole ring and then oxidation and chlorination to a 3-chlorodithiolium salt. Sulfur nucleophiles formed from sulfur and triethylamine are likely to produce a thione group.

**Scheme 28:**
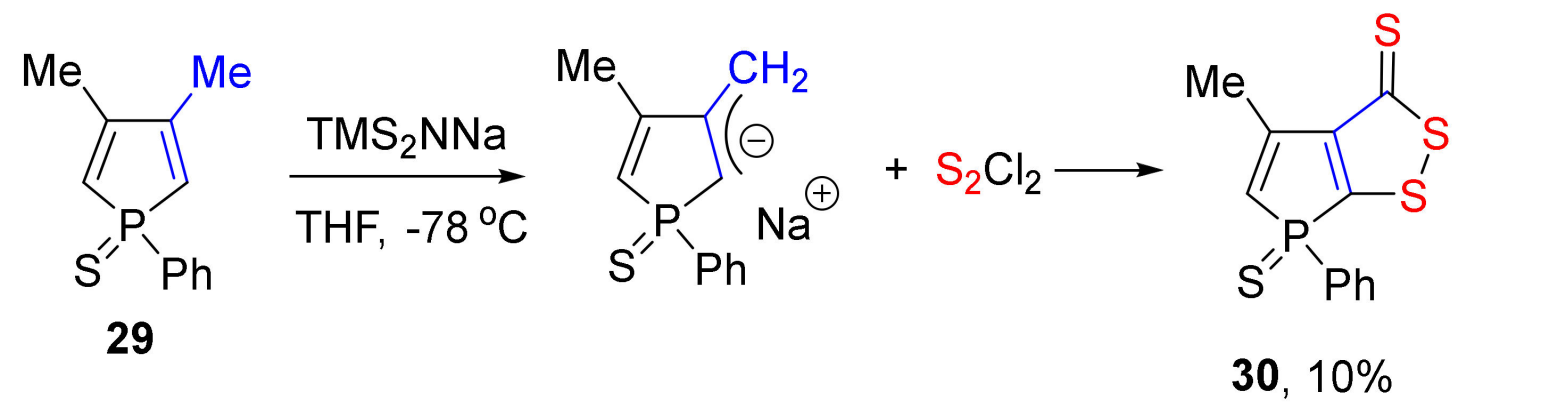
Synthesis of 1,2-dithiole-3-thione annulated with phosphole sulfide.

Interestingly, almost simultaneously with this work, a paper appeared describing the synthesis of 1,2-dithiole-3-thione annulated with a phosphole sulfide ring **30** [70] from the corresponding heterocycle **29**, in which the methyl and C-H groups are at *ortho*-positions to each other (Scheme 28). A successful reaction with S_2_Cl_2_ requires activation with a base (in this case, sodium hexamethyldisilazide, (Me_3_Si)_2_NNa); the yield of fused dithiolethione **30** was as small as 10%.

**Scheme 29:**
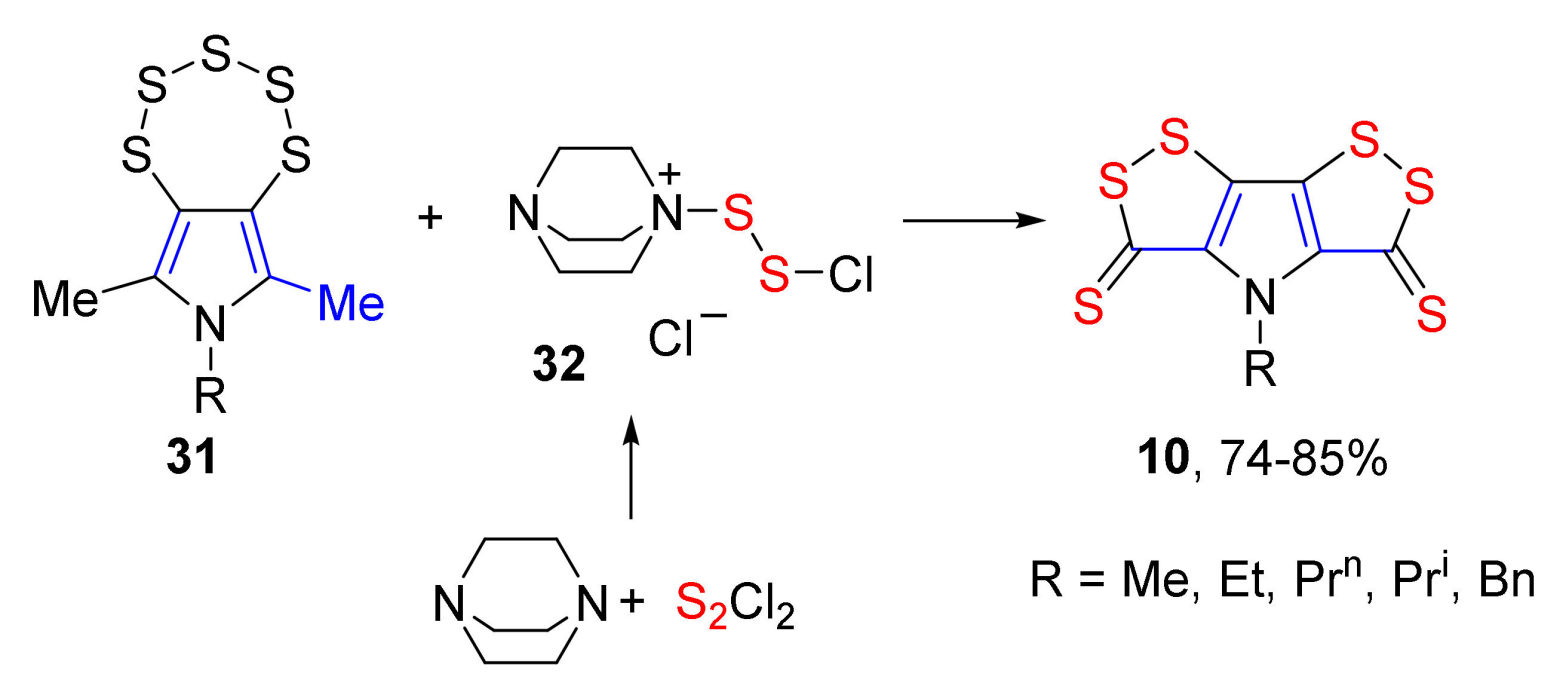
Reaction of pentathiepinopyrroles **31** with salt **32**.

Pentathiepinopyrroles **31** reacted with salt **32** obtained from disulfur dichloride and DABCO to give bis(1,2-dithiolo)pyrroles **10** in high yields [71]. Pentathiepins usually do not react with the S_2_Cl_2_ - DABCO system at room temperature; therefore, it was assumed that salt **32** reacted with methyl groups as an electrophilic reagent to afford bis(1,2-dithiolo)pyrroles **10** in a complex cascade transformation (Scheme 29).

### 2.6. Miscellaneous Syntheses of 3H-1,2-dithiole-3-thiones

**Scheme 30:**
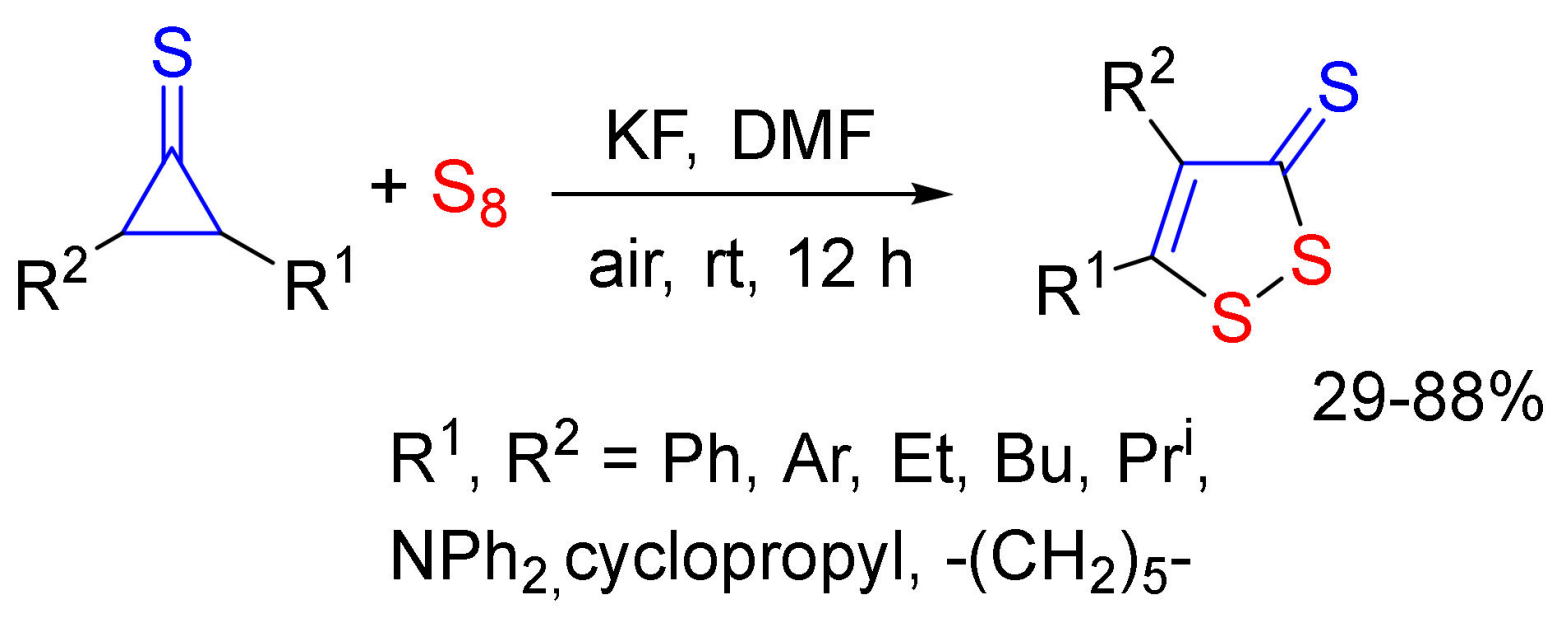
Synthesis of 1,2-dithiole-3-thiones from cyclopropenthione derivatives.

A new procedure was suggested to synthesize 1,2-dithiole-3-thiones from cyclopropenthione derivatives and elemental sulfur in the presence of potassium fluoride in DMF in an air or oxygen atmosphere [72]. The features of the reaction include a high efficiency and good regioselectivity with respect to a wide range of cyclopropenthione derivatives (Scheme 30). The suggested mechanism for this [3+2] cycloaddition includes attack of elemental sulfur on the positively charged carbon atom of the cyclopropenthione derivative followed by release of S_6_ with a further ring-opening/cyclization sequence to give dithiolethiones.

**Scheme 31:**
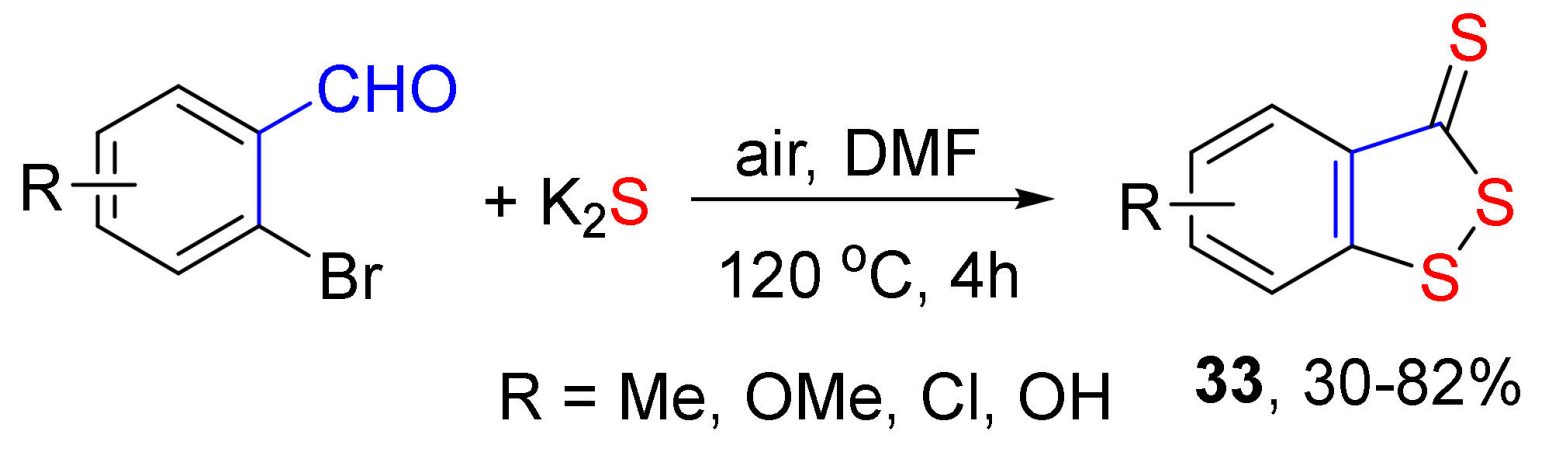
Synthesis of substituted 3*H*-benzo[1,2]dithiole-3-thiones.

Substituted 3*H*-benzo[1,2]dithiole-3-thiones **33** can be prepared by treatment of 2-bromobenzaldehydes with potassium sulfide in DMF [73]. This method is superior to those previously described for benzodithiolethiones in terms of the number of steps and efficiency (Scheme 31). A possible mechanism of this reaction involves aromatic substitution of 2-bromobenzaldehyde with potassium sulfide followed by the reaction with elemental sulfur formed by oxidation of potassium sulfide with atmospheric oxygen and the subsequent Willgerodt–Kindlertype reaction, which affords the final benzodithiolethione.

**Scheme 32:**
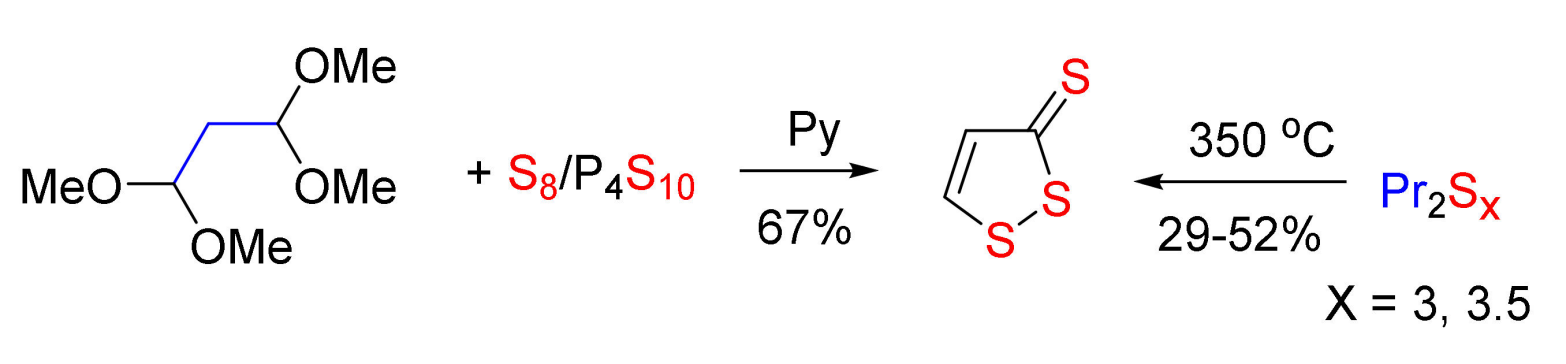
Synthesis of the parent 3*H*-1,2-dithiole-3-thione.

Two procedures have been suggested for the synthesis of the parent 3*H*-1,2-dithiole-3-thione (1,2-Trithione). Treatment of commercially available malonodialdehyde dimethylacetal by heating at 130 °C with a mixture of elemental sulfur and P_4_S_10_ in pyridine gave 3*H*-1,2-dithiole-3-thione in a good yield [74]. Thermolysis of dipropyl polysulfides (*n*-Pr)_2_S_x_ (x = 3, 3.5) at 350 °C gave 1,2-dithiole-3-thione in moderate yield [75]; the process was accompanied by the evolution of gaseous products such as hydrogen sulfide, propylene and hydrogen (Scheme 32).

## 3. Reactions of 3*H*-1,2-dithiole-3-thiones

There are several typical reactions of 1,2-dithiole-3-thiones that have been studied for decades: 1,3-dipolar cycloaddition to alkynes, isonitriles, nitrilimines, various transformation of thione group, as well as some new transformations that have been discovered recently: recyclization to other heterocycles, insertion of several fragments into an S-S bond, and some others.

### 3.1. Reactions with Alkynes

**Scheme 33:**
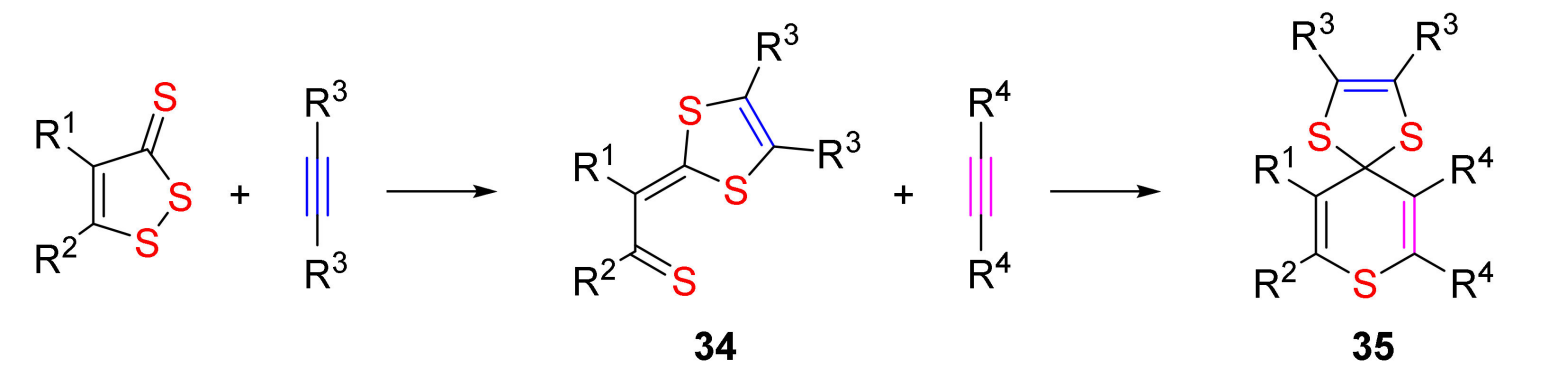
1,3-Cycloaddition of alkynes to 1,2-dithiole-3-thiones.

Dithiolethiones can sequentially add one or two alkyne molecules to initially give 1,3-dithioles **34** and then spiro-1,3-dithiolothiopyrans **35** (Scheme 33).

**Scheme 34:**
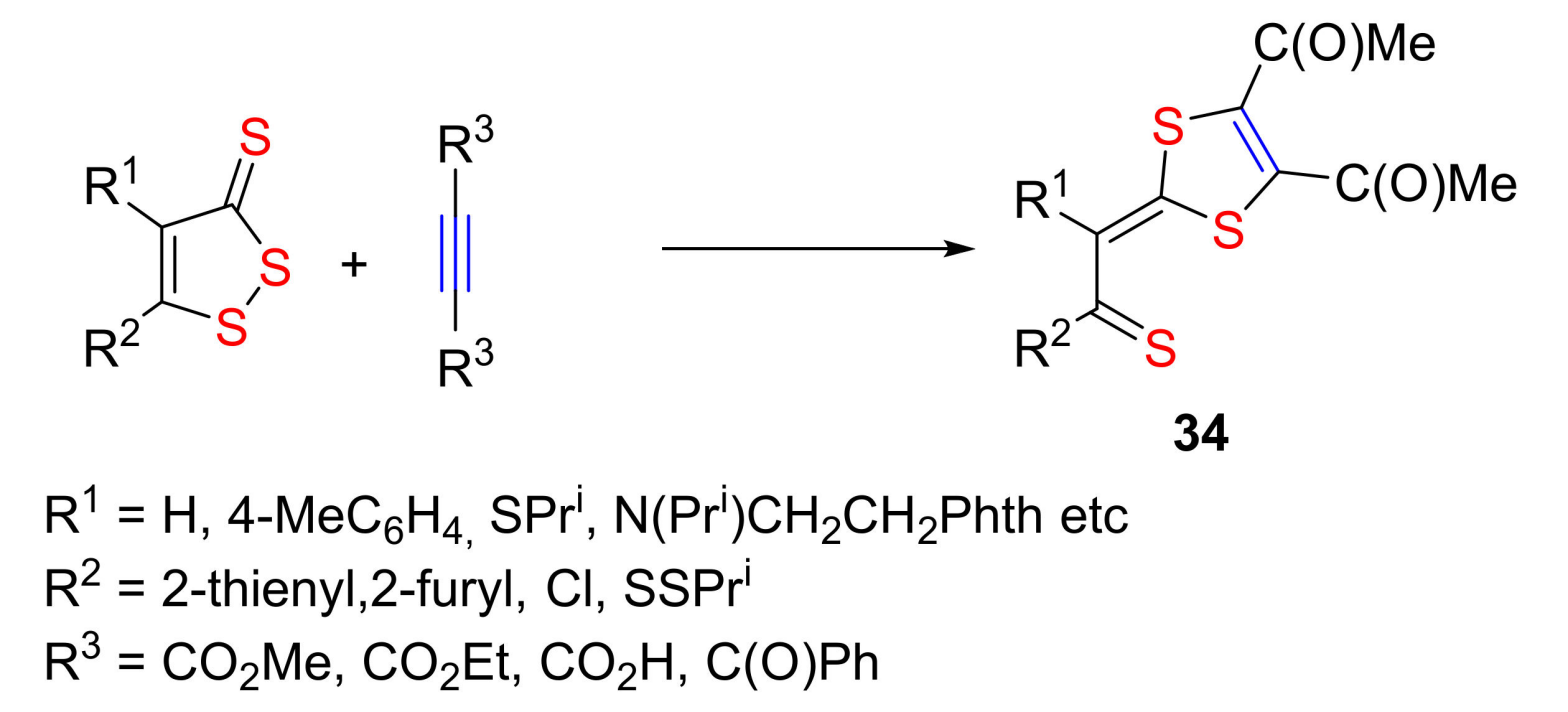
Synthesis of 1,3-dithioles.

As a rule, the reaction of non-fused dithiolethiones is stopped at the stage of addition of one mole of an alkyne (Scheme 34). Usually, one equivalent of an alkyne should be added to the reaction mixture to obtain 1,3-dithioles **34**, and the conversion is carried out at room temperature or even below it [37,76,77], but in some cases refluxing with an excess of an alkyne in xylene or benzene was employed [64,66,78,79]. The yields of 1,3-dithioles can vary widely from 20% to the quantitative yield.

**Scheme 35:**
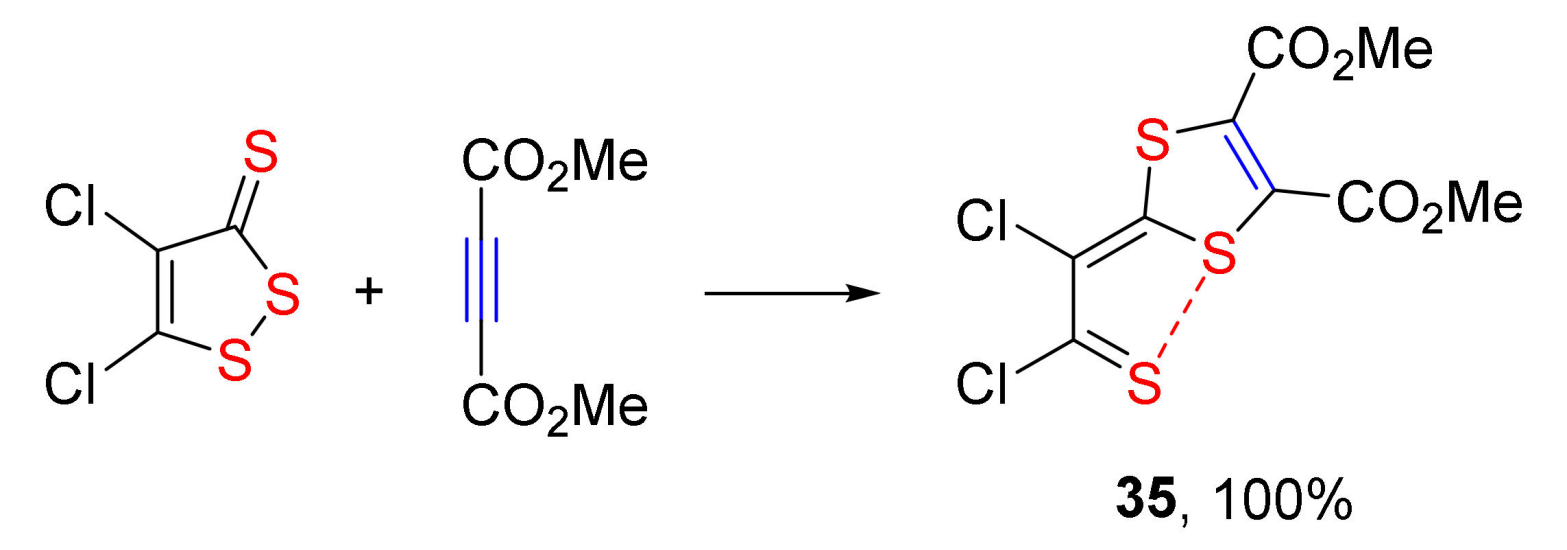
Synthesis of 1,3-dithiole **35**.

It is noteworthy that in the case of 5-chlorosubstituted dithiolethiones, rare aliphatic compounds with a usually unstable thioacyl chloride group are formed [80]. The stability of compound **35** is due to the intramolecular interaction between the thiocarbonyl group and the heterocyclic sulfur atom that can reduce the electrophilicity of the thiocarbonyl group (Scheme 35). In the case of compound **35**, this was confirmed using X-ray diffraction analysis, which showed the planarity of the quasibicyclic part of the molecule with an S...S distance of 2.91 Å. It is in the range between the length of the usual S-S bond (2.05 Å) and the sum of van der Waals radii for these atoms (3.68 Å).

**Scheme 36:**
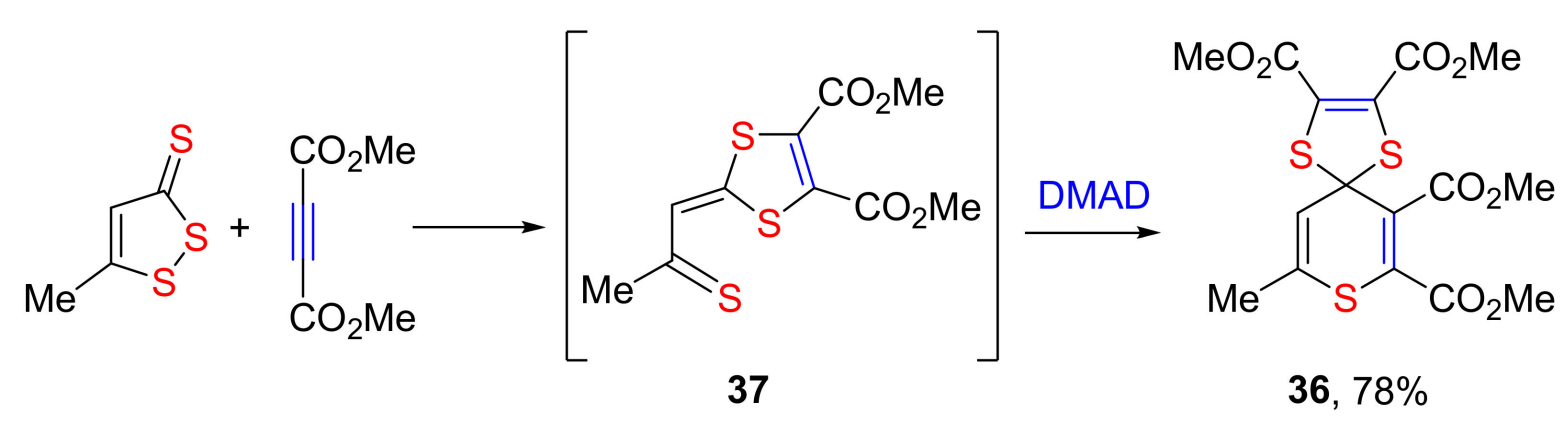
Synthesis of thiopyrane **36**.

In light of the above, rather an unexpected result was obtained in the reaction of 5-methyl-3*H*-1,2-dithiole-3-thione with DMAD. When these reagents were refluxed in chloroform for 5 h, only the addition product of two DMAD molecules, thiopyrane **36**, was isolated in 78% yield [81]. However, it should be noted that the authors did not attempt to obtain the intermediate 1,3-dithiole **37** (Scheme 36).

**Scheme 37:**
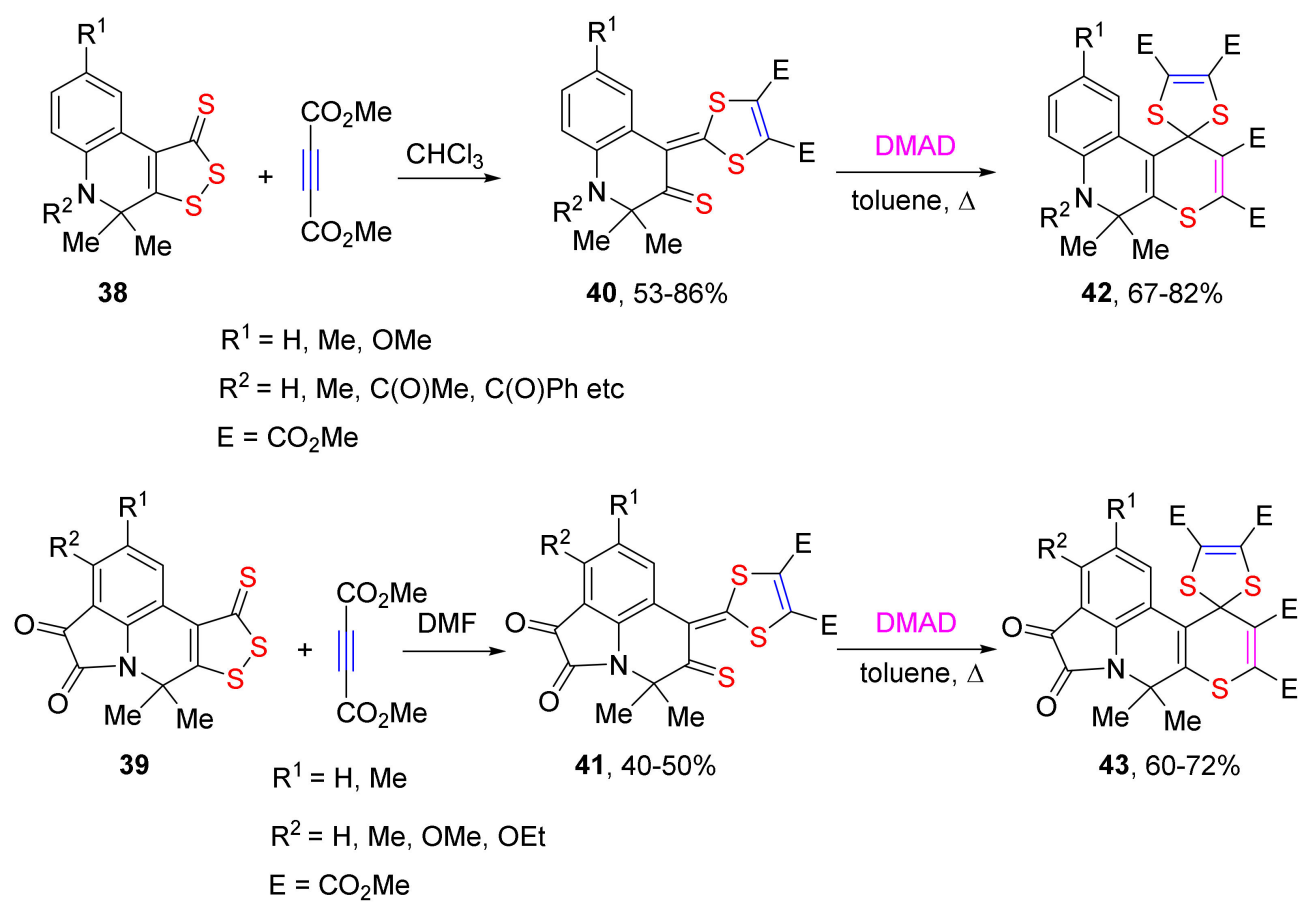
1,3-Cycloaddition of [l,2]dithiolo[3,4-*c*]quinoline thiones **38** and **39**.

As a rule, 1,2-dithiole-3-thiones fused with non-aromatic and heteroaromatic rings sequentially add first one and then the second mole of an activated alkyne. Moreover, it was often difficult to stop the reaction at the first stage, and as a result, 1,3-dithioles were isolated in low yields. In fact, the reactions of [l,2]dithiolo[3,4-*c*]quinoline thiones **38** and **39** gave 1,3-dithioles **40** and **41** at room temperature in chloroform [82,83] or dimethylformamide [84], while the addition of the second mole of DMAD was performed by refluxing in toluene (Scheme 37).

**Scheme 38:**
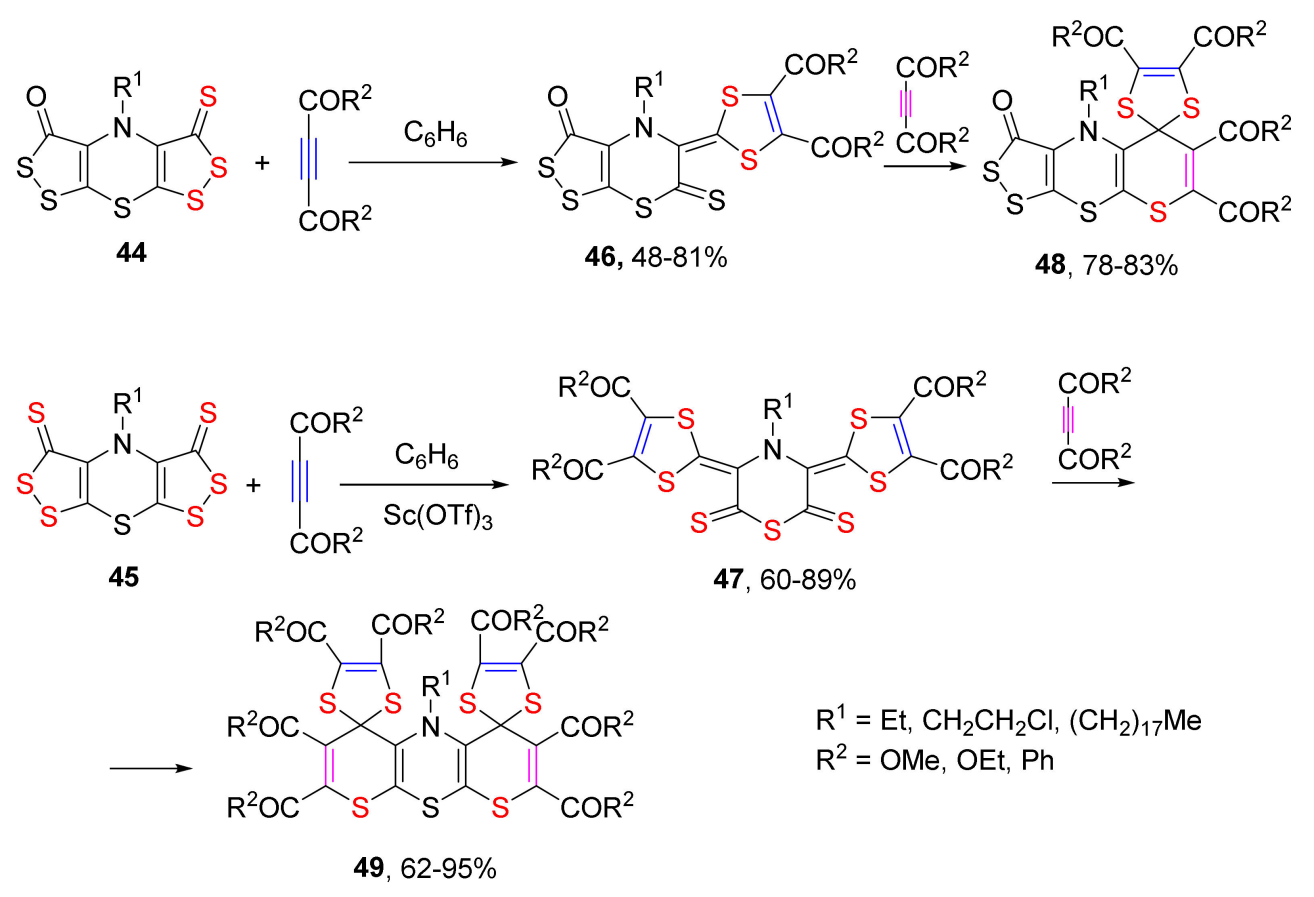
Reaction of bis-dithiolothiazines **44** and **45** with DMAD.

Similar results were obtained in the reaction of bis-dithiolothiazines with DMAD and dibenzoylacetylene [85,86]. Short-term refluxing (10–45 min) of monothiones **44** or bis-thiones **45** with one equivalent of an alkyne in benzene gave mono- **46** and bis(1,3-dithioles) **47** in moderate to high yields (Scheme 38). The use of scandium triflate as a catalyst increased the yields of 1,3-dithioles significantly, up to 60–80%. If excess alkyne was added to monothiones **44** or bis-thiones **45** in refluxing toluene or benzene, dithiolopyranes **48** and **49** were formed. It is noteworthy that the yields of 1,3-dithioles and thiopyranes are nearly the same, which indicates that the second alkyne molecule is added quantitatively.

**Scheme 39:**
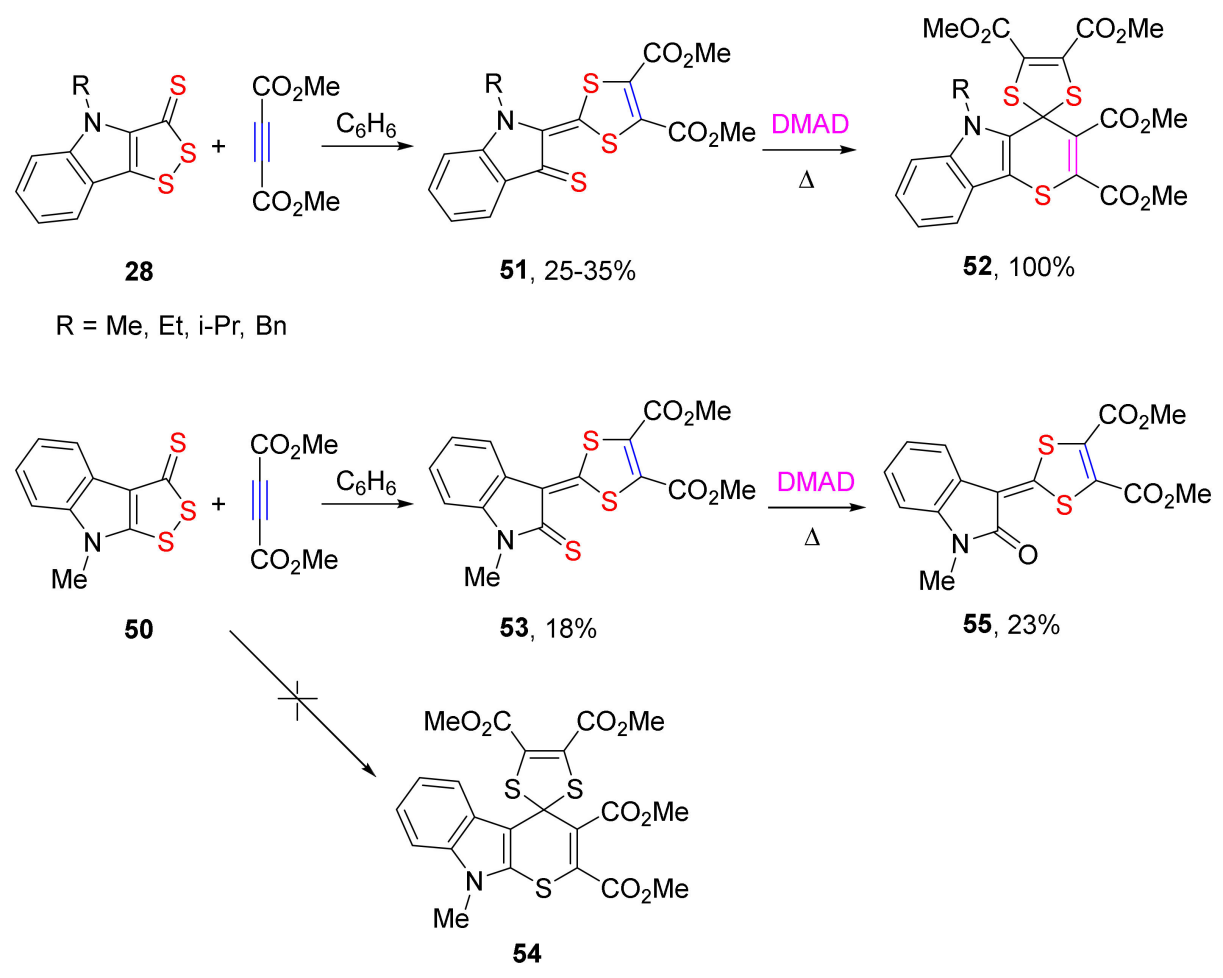
Reaction of dithioloindoles with DMAD.

Interesting results were obtained in a study of the reaction of dithioloindoles **28** and **50** with DMAD [68]. In the reaction of dithiole **28** with DMAD, the first molecule was added somewhat more slowly than the second one, and in all cases, mixtures of mono- and bis-products formed (Scheme 39). The highest yields of 1,3-dithioles **51** (25–35%) were obtained in the reaction with one equivalent of DMAD in benzene at room temperature for three days. Monoadducts **51** reacted with one mole of DMAD in benzene under reflux to form bis-adducts **52** in a quantitative yield in a few minutes. The reaction of thione **50** with excess DMAD in benzene under reflux conditions gave only monoproduct **53** in a low yield (18%). The expected bis-adduct **54** was not detected in the reaction medium; instead of this product, ketone **55** was isolated in 23% yield. The formation of this product was unexpected. In an attempt to explain this result, it was found that it was formed in 65% yield from thione **53** on treatment with DMAD. Thus, thione **53**, which is a regioisomer of thione **51**, in contrast to the latter, does not react with DMAD as with a dienophile. Obviously, the reason for this lies in the different reactivity of the thiono groups in these compounds. The thiono group in **53** is essentially a thioamide group in nature and is therefore not involved in 1,3-dipolar cycloaddition reactions.

**Scheme 40:**
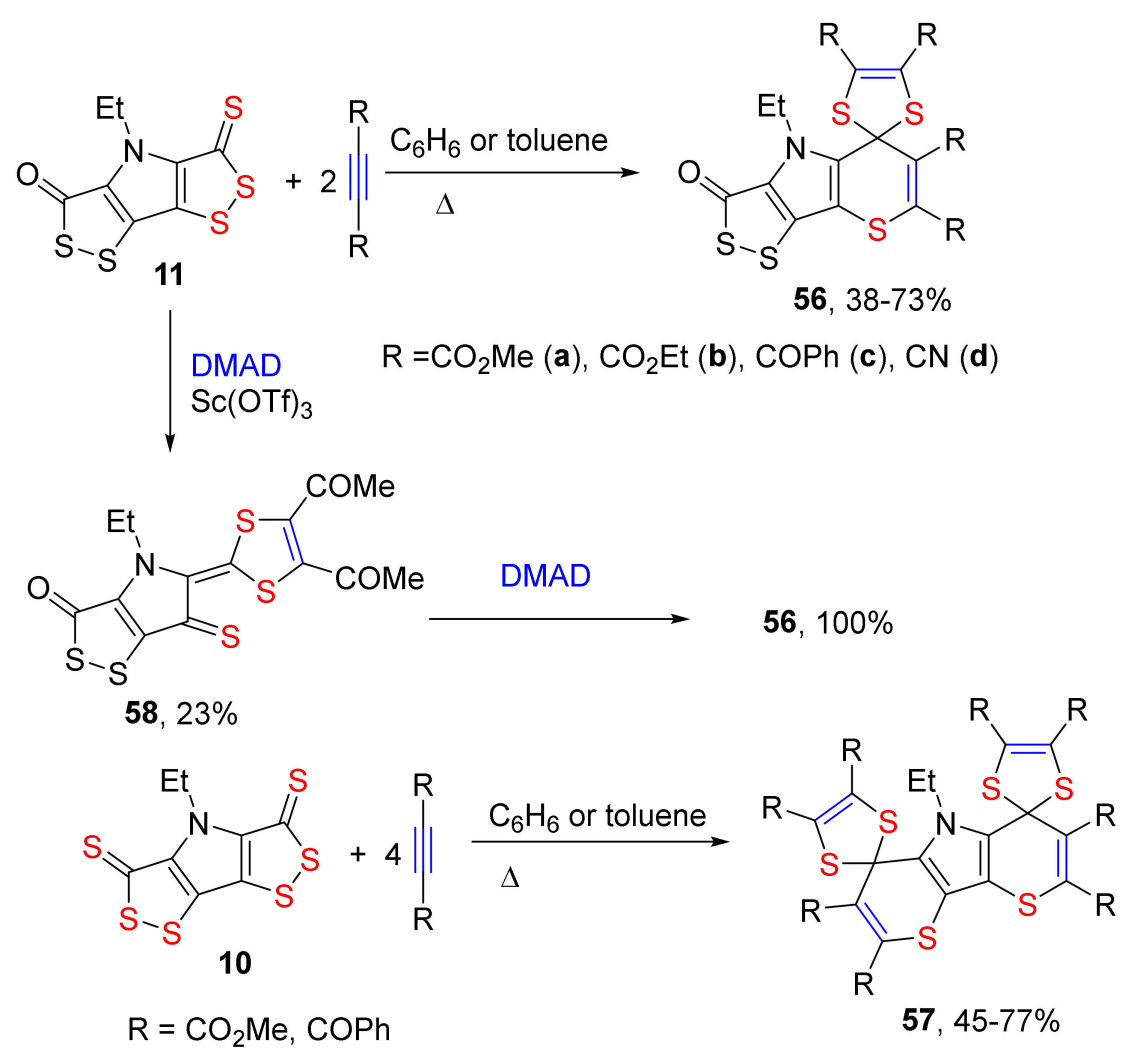
Reaction of bis[1,2]dithiolopyrroles **10** and **11** with activated alkynes.

In the case of monothioxo **11** and bis-thioxo **10** bis[1,2]dithiolopyrroles, the reaction occurs with two moles of an activated alkyne per thiono group and ends with the formation of spiro-1,3-dithiolopyrans **56** and **57** [85,86]. Only in one case, in the reaction of thione **11** with one equivalent of DMAD in the presence of scandium triflate (Sc(OTf)_3_), the addition product of one alkyne molecule **58** could be isolated in a low yield. The latter was found to be unstable and decomposed back to thione **11** on storage (Scheme 40). The reaction of **58** with a second equivalent of DMAD readily gave spiro-1,3-dithiolopyrane **56** in quantitative yield. The authors explain the instability of product **58** by the loss of aromaticity of the pyrrole ring, which can be restored upon reaction with the second dienophile molecule or upon its conversion back to the initial bis(dithiolo)pyrrole **11** [85].

**Scheme 41:**
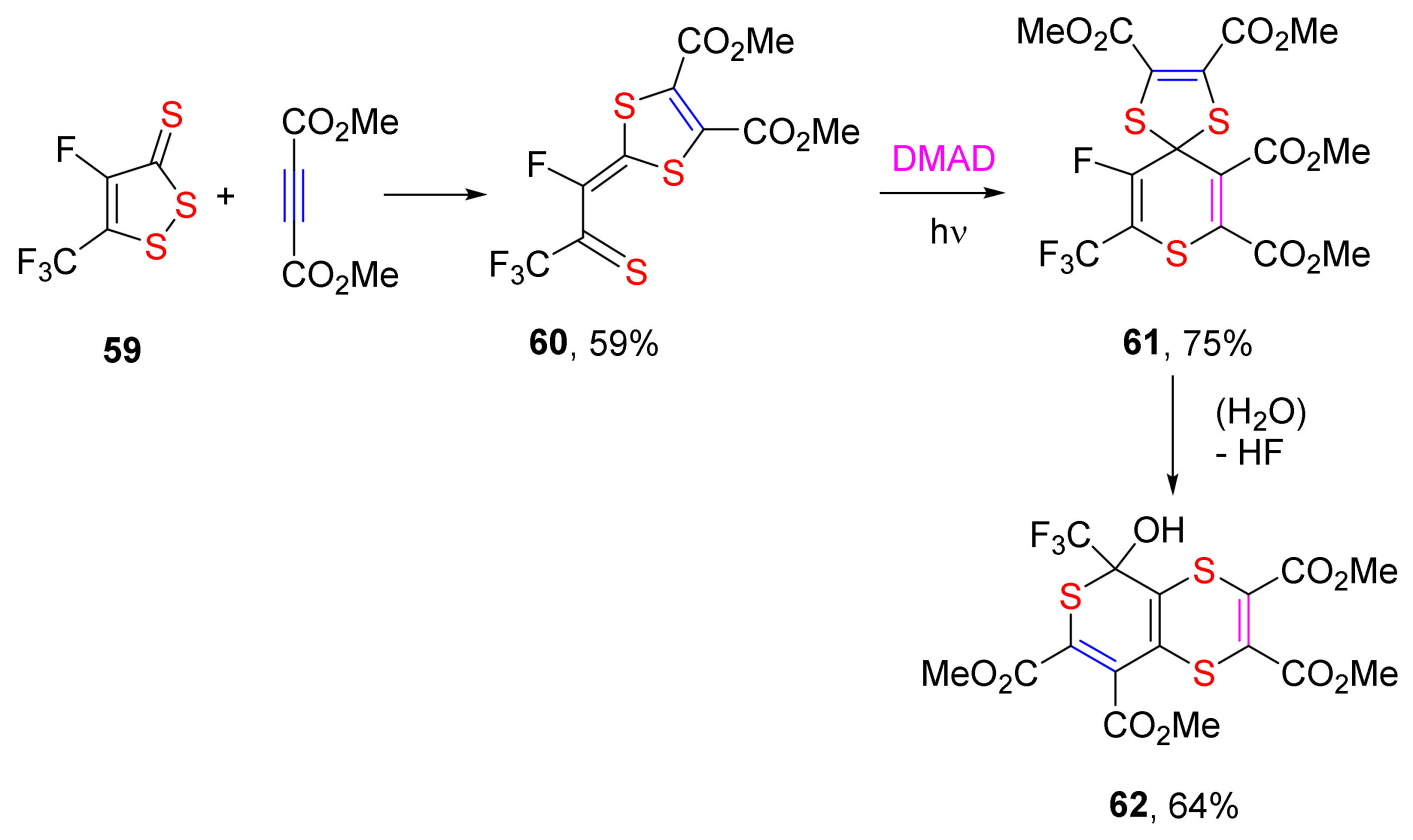
Reaction of 4-fluoro-5-trifluoromethyl-1,2-dithiole-3-thione with DMAD.

The formation of a the thiopyrane structure from 4-fluoro-5-trifluoromethyl-1,2-dithiole-3-thione **59** can occur not only on heating but also under irradiation (Scheme 41). 

As expected, the reaction of thione **59** with DMAD gave 1,3-dithiole **60** at room temperature [40]. Irradiation of a mixture of 1,3-dithiole **60** and DMAD with a 500-volt tungsten lamp for 0.5 h resulted in thiopyrane **61** in 75% yield. However, this substance turned out to be unstable to moisture contained in silica gel and, after filtration through its layer, it is converted to hydroxy adduct **62** in a yield of 64%. It is noteworthy that all the reactions can be carried out in one flask from thione **59** without isolating the intermediate products.

The 1,3-dipolar cycloaddition to unsymmetrical alkynes has been studied less thoroughly. There are several reasons for this fact. The incorporation of hydrogen atoms or a phenyl group into an alkyne molecule instead of strong electron-deficient substituents, such as ester (CO_2_Alk), benzoyl (C(O)Ph), or nitrile (CN) groups, significantly reduces the reactivity of alkynes in these processes. In addition, reactions with unsymmetrical alkynes result in mixtures of regioisomers that are difficult or almost impossible to separate, which reduces the synthetic value of this method.

**Scheme 42:**
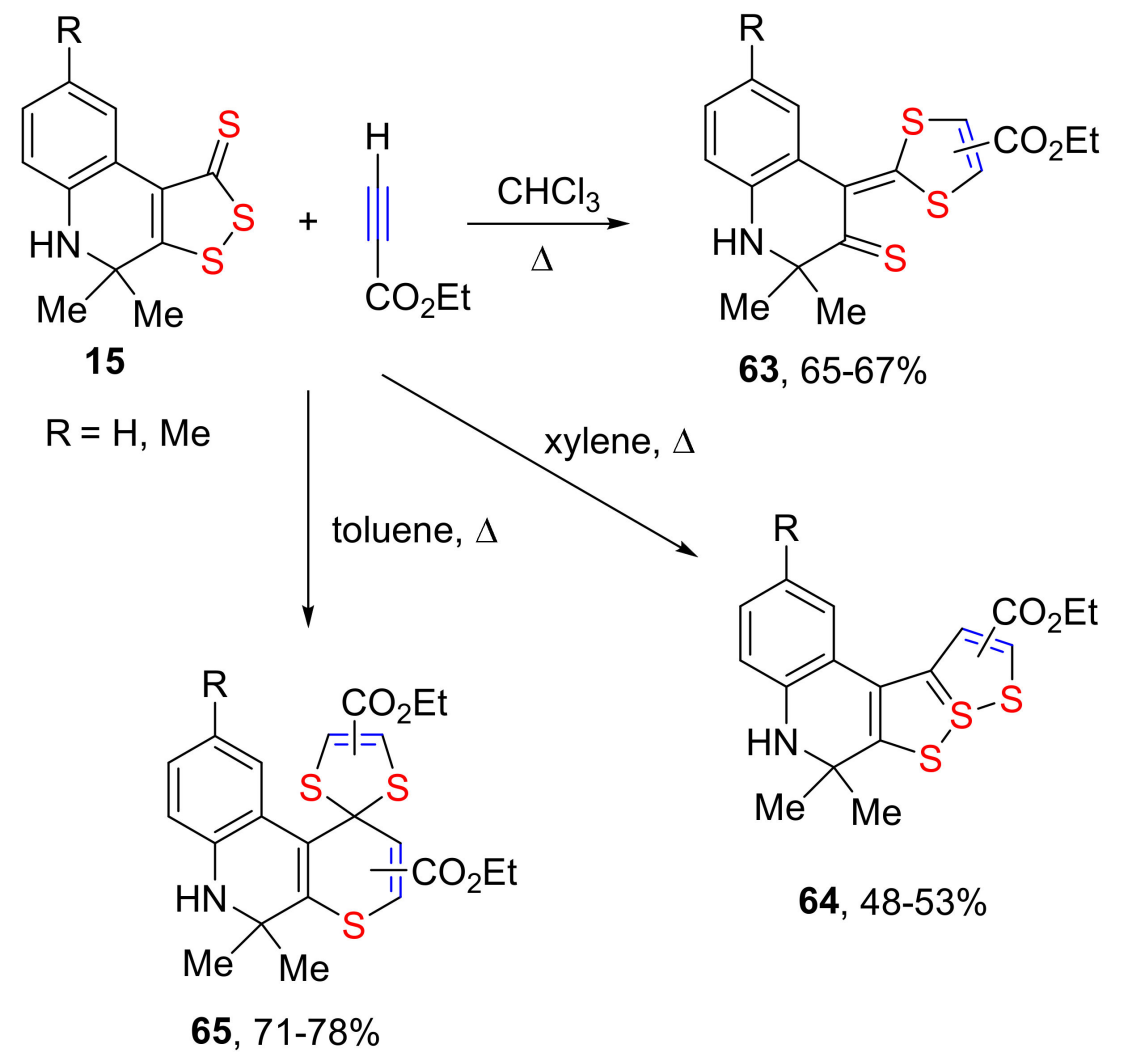
Reaction of 4,5-dihydro-4,4-dimethyl-[1,2]dithiolo[5,4-*c*]quinoline-1-thiones with ethyl propiolate.

Shikhaliev et al. studied the cycloaddition of ethyl propiolate to 4,5-dihydro-4,4-dimethyl-[1,2]dithiolo[5,4-*c*]quinoline-1-thiones **15** and showed that, depending on the conditions used (solvent and temperature), the reaction can occur quite selectively both with one or two alkyne molecules and with cleavage of the thioketone bond as [2+2] cycloaddition followed by recyclization (Scheme 42). For example, refluxing equimolar amounts of reagents in chloroform gave 1,3-dithioles **63**. Treatment of ethyl propiolate with thiones **15** at a higher temperature (in boiling xylene) occurs with cleavage of the thioketone bond followed by recyclization to trithiapentalenes **64**. The reaction in toluene with two moles of ethyl propiolate afforded 1:2 adducts **65** in high yields. In all cases, several sets of signals from carbethoxy groups and quasi-aromatic protons were observed in the ^1^H NMR spectra, which indicates that these reactions are not regioselective [87].

**Scheme 43:**
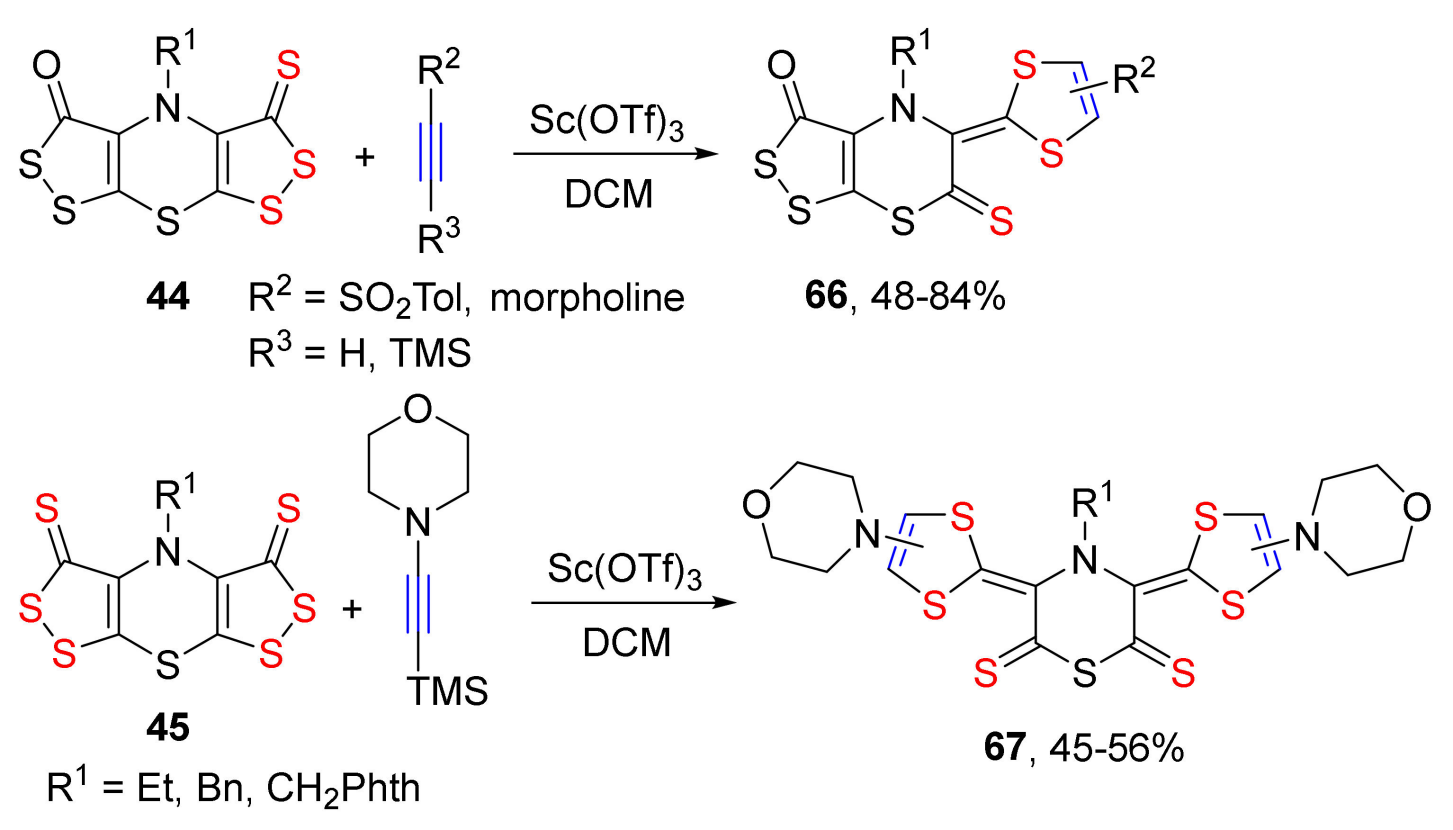
Reaction of bis(dithiolo)thiazines with unsymmetrical alkynes.

The reaction of tricyclic bis(dithiolo)thiazines **44** and **45** with an excess of a terminal alkyne activated by sulfonyl or trimethylsilyl groups in the presence of scandium triflate involved one or two alkyne molecules to give a pair of regioisomers **66** and **67** [58,88]. The lack of regioselectivity found in products **66** and **67** may be a result of exocyclic double bond isomerization after the cycloaddition (Scheme 43).

**Scheme 44:**
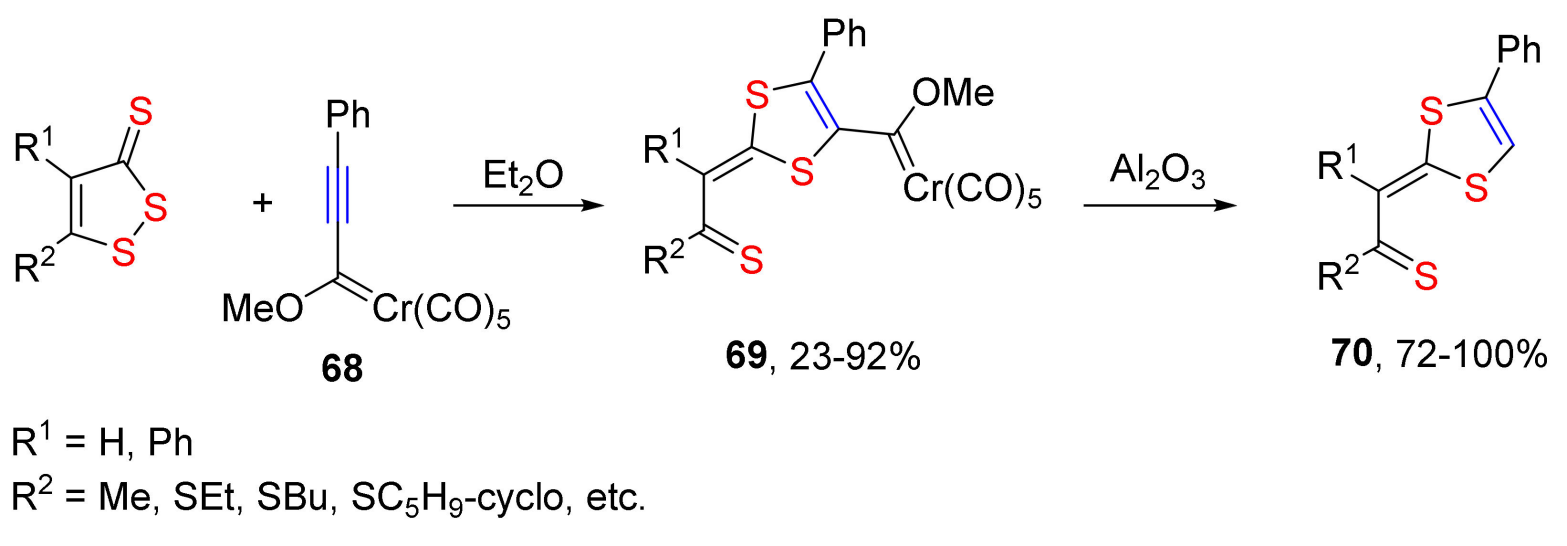
Reaction of 1,2-dithiole-3-thiones with Fischer’s phenylethyl carbene complex **68**.

A notable and rare example of regioselective addition of Fischer’s phenylethyl carbene complex **68** to 1,2-dithiole-3-thiones was reported by Rossi and Torroba [89]. The carbene moiety of Fischer’s alkyne acted as an electron-withdrawing group increasing the electrophilic character of alkyne **68** (Scheme 44). The reaction occurred at −40 °C in diethyl ether. The yield of compounds **69** varied quite widely from low (23%) to high values (92%). Treatment of products **69** with neutral alumina followed by chromatography of the products afforded *E*-dithiofulvalenthione **70** in high yields.

**Scheme 45:**
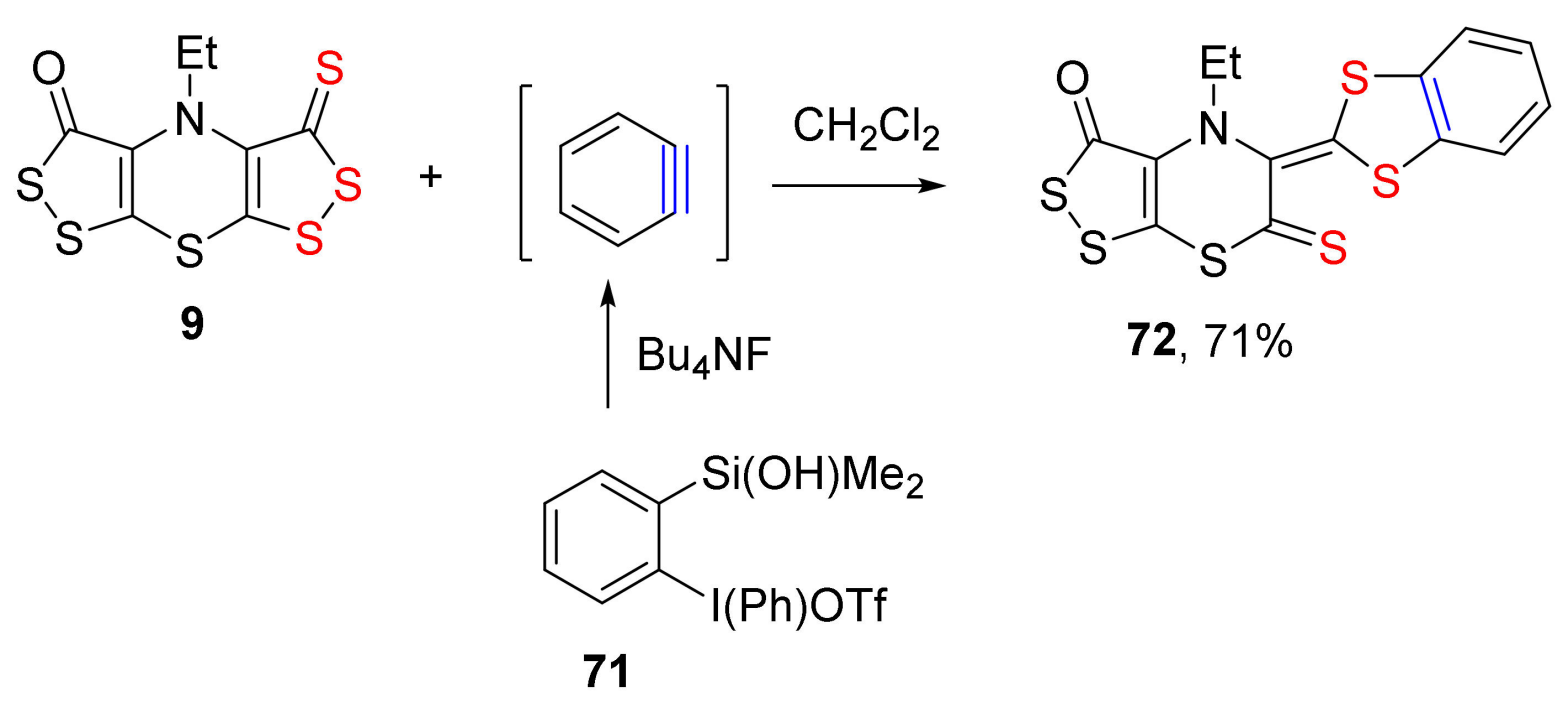
Reaction of dithiolethione **9** with benzyne.

The possibility of benzyne addition to dithiolethione **9** was studied [58]. It was found that the majority of the known methods for generating benzyne were unsuitable for cycloaddition in this case (Scheme 45). The only successful method for synthesizing the cycloaddition product **72** in a high yield from benzyne was implemented using one of the mildest methods for benzyne generation from iodonium salt **71**.

### 3.2. Reactions with Alkenes

**Scheme 46:**
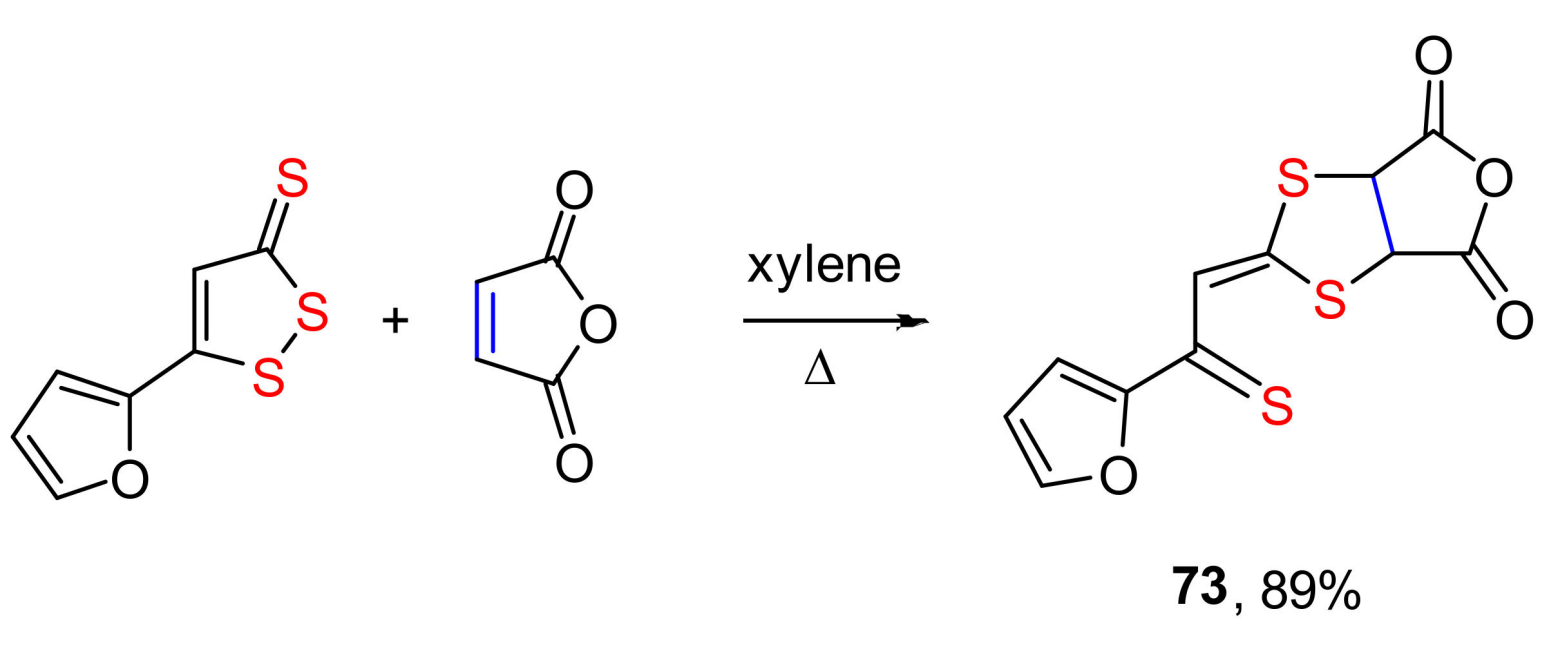
Reaction of 5-(2-furyl)-1,2-dithiole-3-thione with maleic anhydride.

Activated alkenes can also react with 1,2-dithiole-3-thiones. However, this reaction rarely becomes the subject of studies, apparently because it can give a mixture of stereoisomers that are difficult to separate and identify. It was shown that 5-(2-furyl)-1,2-dithiole-3-thione reacted with maleic anhydride in boiling xylene to give 1,3-dithiolane **73** in high yield (Scheme 46) [77].

**Scheme 47:**
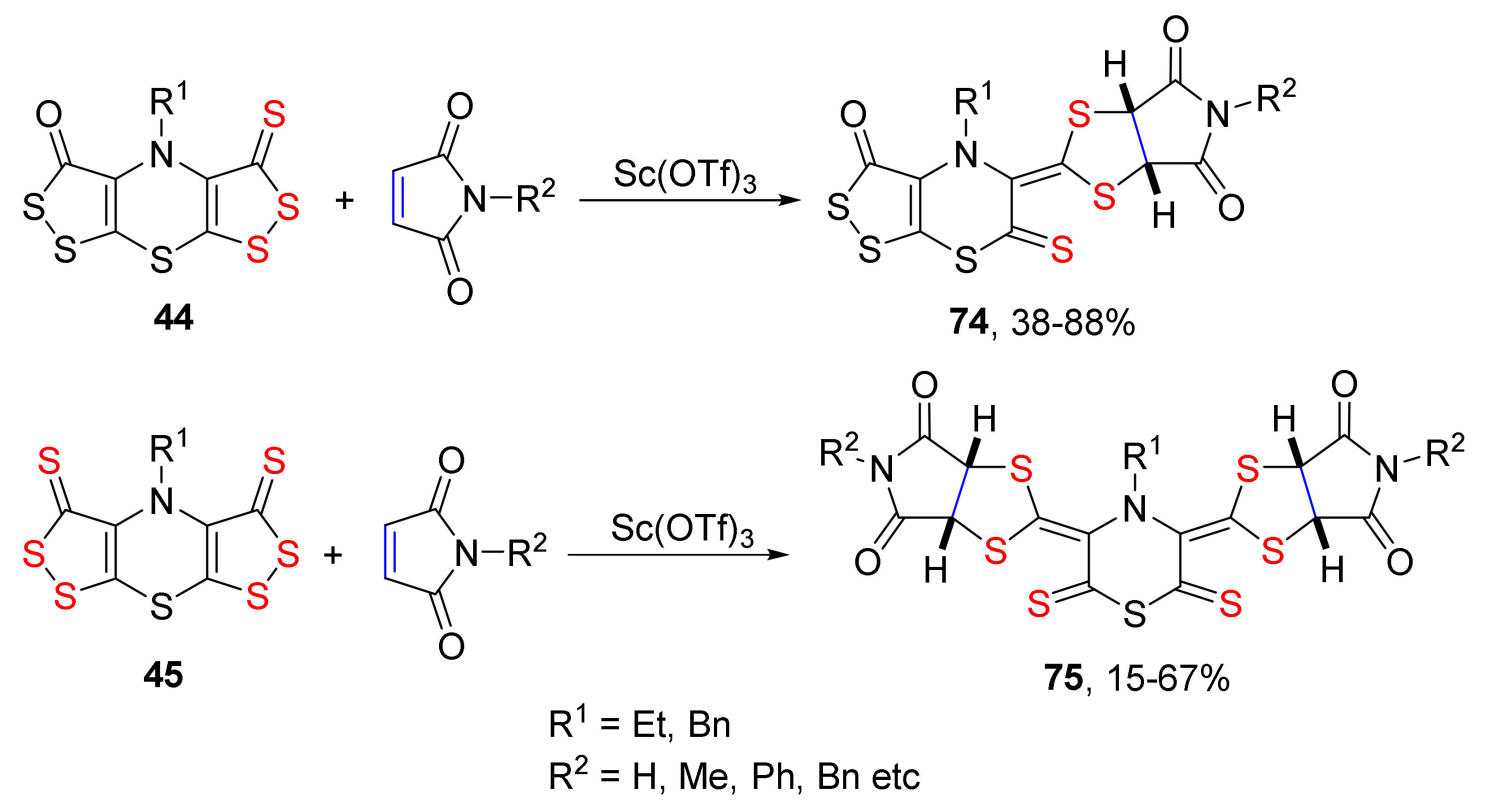
Reaction of bisdithiolothiazines **44** and **45** with maleimides.

Treatment of bisdithiolothiazines **44** and **45** with commercial maleimides in the presence of scandium triflate, a catalyst which was very efficient in the 1,3-cycloaddition of polyheterocyclic dithiolethiones and activated alkynes [58,88], gave the corresponding mono- **74** and bis-adducts **75** with two or four chiral centers. The ^1^H NMR spectra of the latter were quite difficult to interpret (Scheme 47) [90].

### 3.3. Reactions with Isonitriles

**Scheme 48:**
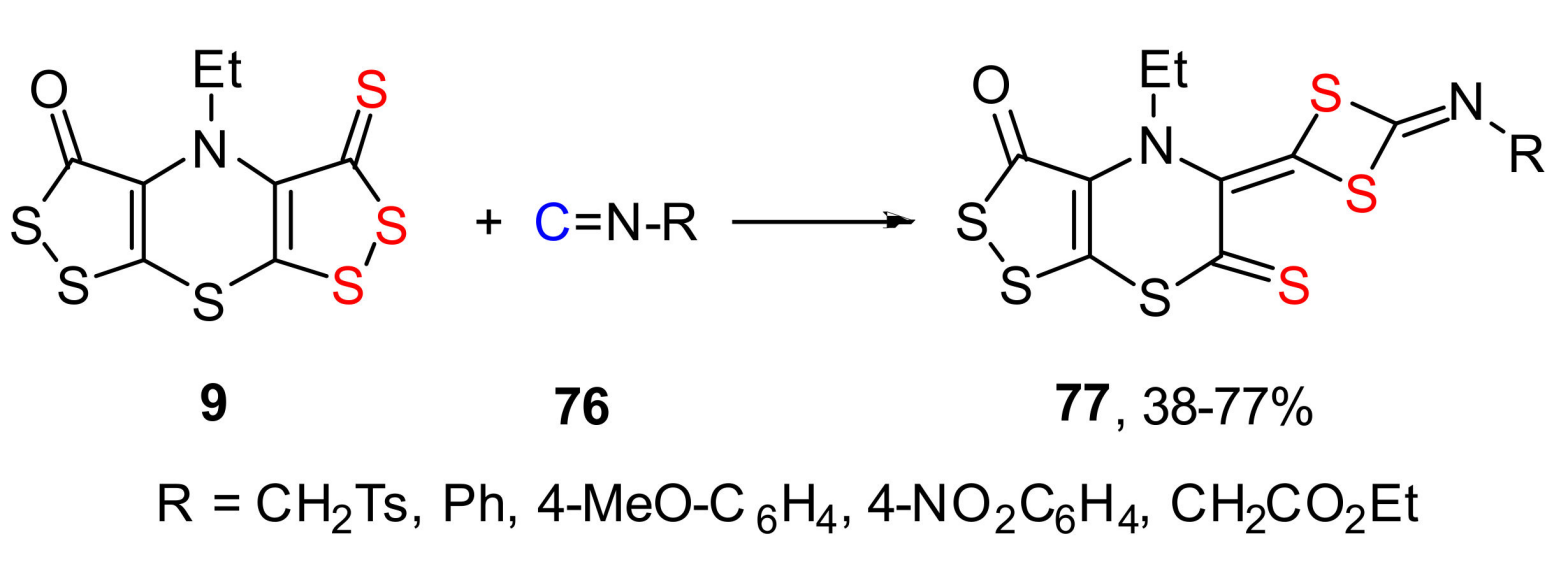
Reaction of ketothione **9** with TOSMIC.

Isonitriles can undergo cycloaddition to 1,3-dipoles and multiple bonds, including those containing sulfur [91]. About 20 years ago, the reaction of ketothione **9** with *p*-toluenesulfonylmethyl isocyanide (TOSMIC) was reported that resulted in imino-1,3-dithietane **77** [92]. Other isonitriles **76** also readily reacted with ketothione **9** to give the corresponding imino-1,3-dithiethanes **77** in moderate to high yields (Scheme 48) [93].

**Scheme 49:**
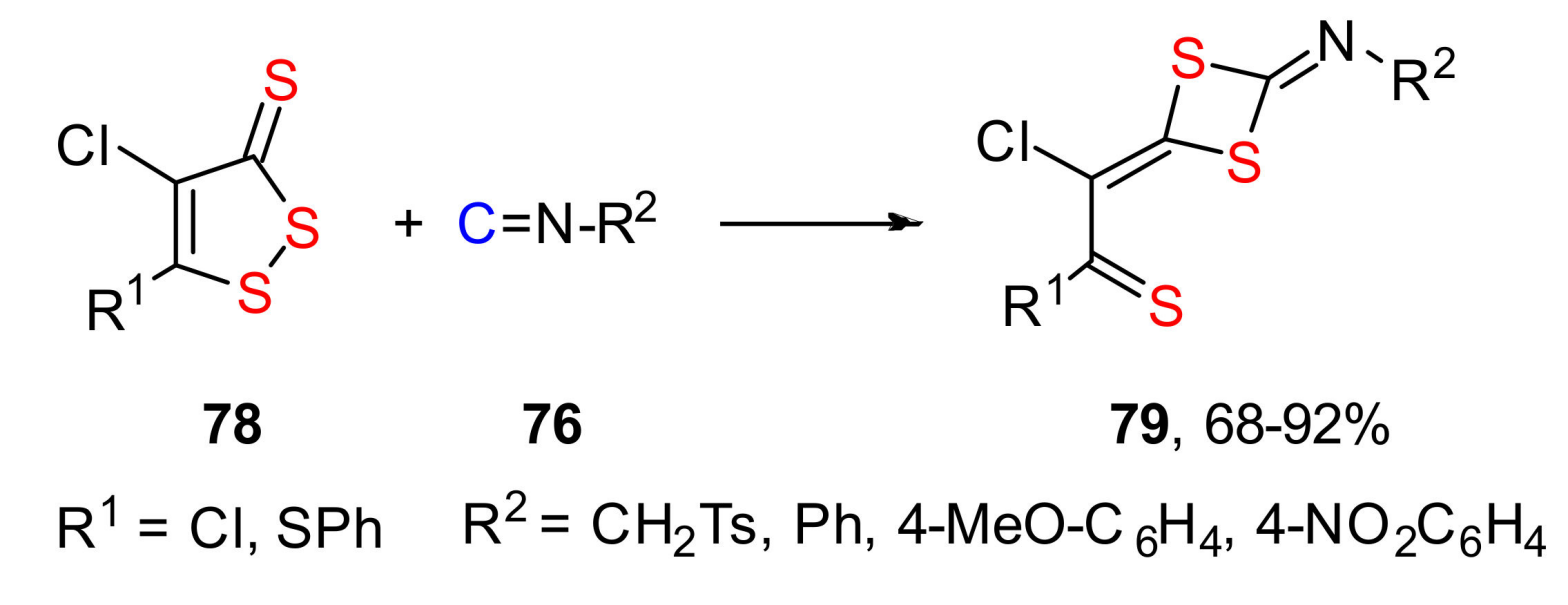
Synthesis of 1,3-dithiethanes **79**.

The possibility of the formation of 1,3-dithietane highly depended on the structure of 1,2-dithiole-3-thione [93]. Thus, monocyclic dithiolethiones **78** containing electron-withdrawing chlorine atoms and a phenylthio group in the molecule reacted with isonitriles **76** at room temperature in benzene to afford 1,3-dithiethanes **79** in high yields (Scheme 49).

**Scheme 50:**
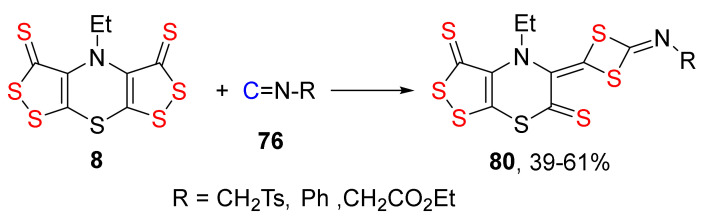
Reaction of bis[1,2]dithiolo[1,4]thiazine **8** with isonitriles.

Bis[1,2]dithiolo[1,4]thiazine **8** contains two 1,2-dithiole-3-thione rings that might be able to react with isonitriles **76** (Scheme 50). However, it was shown that only one isonitrile group of dithione **8** underwent cycloaddition of isonitriles **76** at room temperature to give 1,3-dithietanes **80**. If excess isonitrile was used and/or the reaction mixture was heated, no new products formed.

**Scheme 51:**
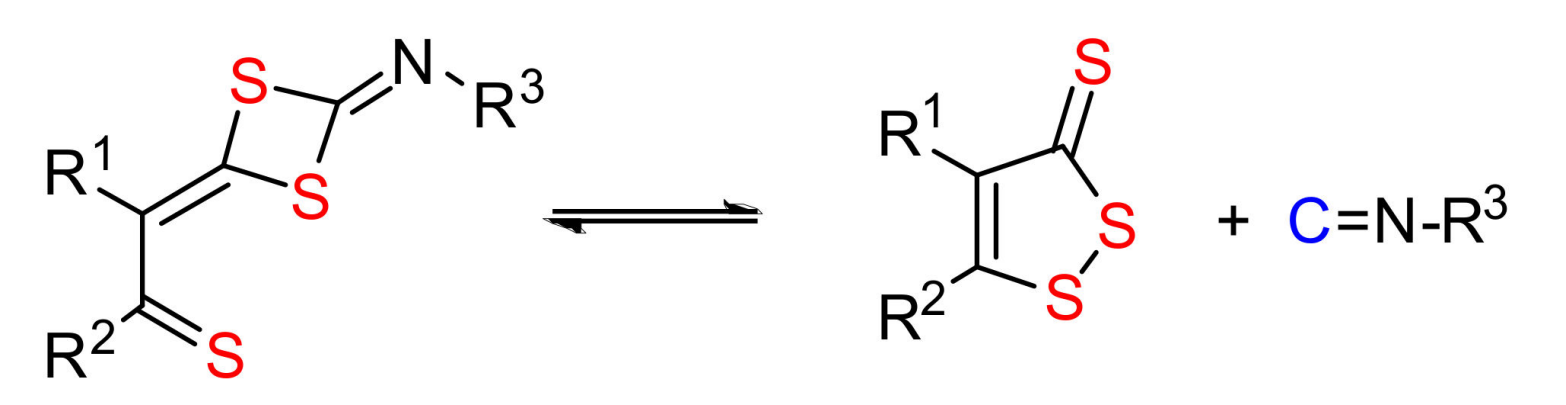
Equilibrium of 1,3-dithietanes with the corresponding 1,2-dithiole-3-thiones and isonitriles.

^1^H-NMR and IR spectroscopy data showed that 1,3-dithietanes in solution are in equilibrium with the starting compounds used for their synthesis, i.e., 1,2-dithiole-3-thione and isonitrile. When these solutions are cooled to −20 °C and kept for one week at this temperature, the equilibrium is completely shifted towards 1,3-dithietane, and when a solution of dithiethane is refluxed in chloroform, it disappears within 10 min, and only the dithiolethione and isonitrile remain (Scheme 51). It has been shown that the result of the equilibrium depends on the structure of both dithiolethione and isonitrile, while the amount of the 1,3-dithietane in the solution can vary from 20 to 98%.

### 3.4. Reactions with Nitrilimines

The reaction of nitrilimines with 1,2-dithiole-3-thiones occurs as 1,3-dipolar cycloaddition at the thiono group and is accompanied by spontaneous opening of the dithiole ring with extrusion of the sulfur atom and formation of a 1,3,4-thiadiazole ring.

**Scheme 52:**
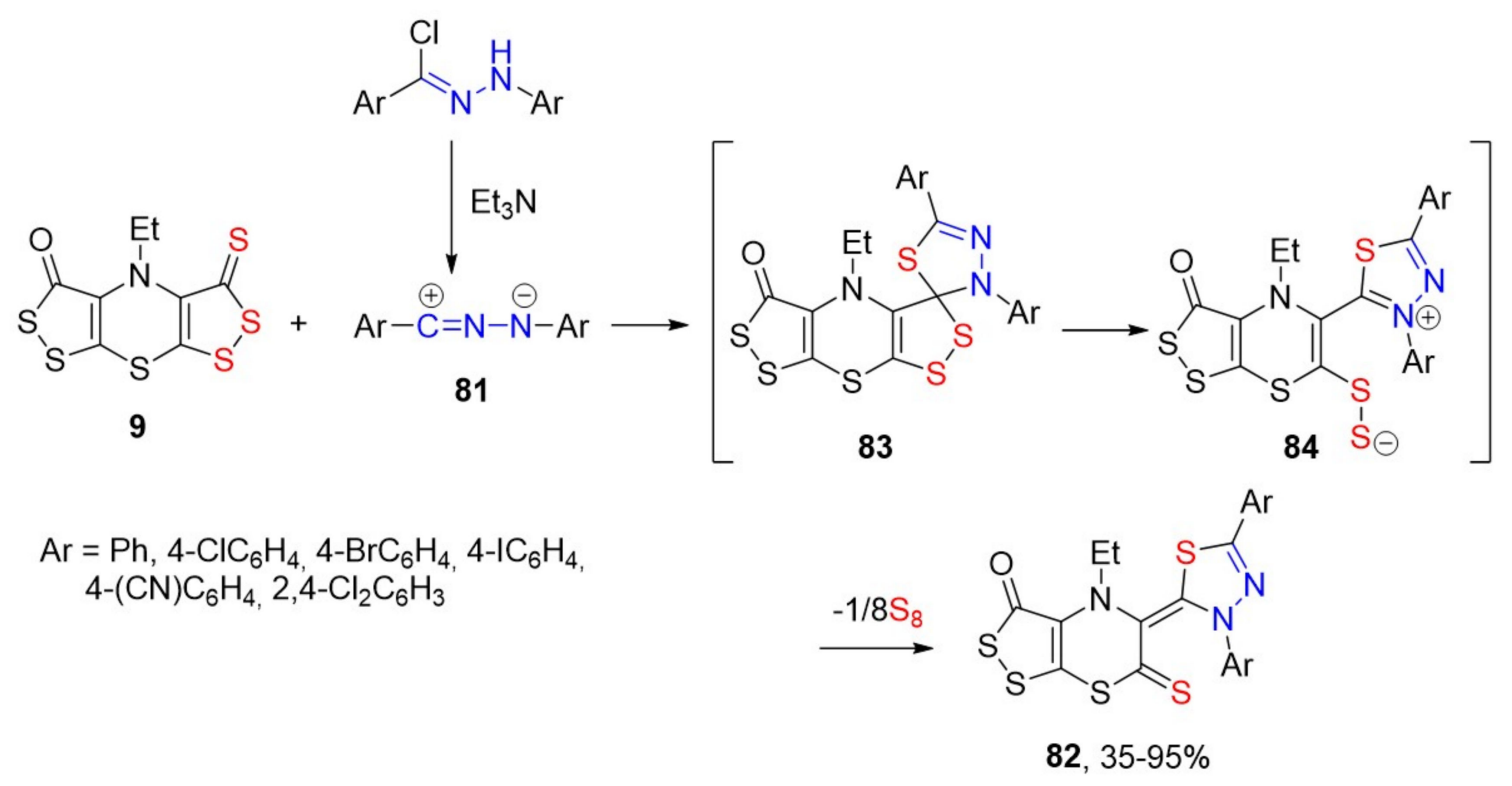
Reaction of bis[1,2]dithiolo[1,4]thiazine **9** with nitrilimines **81**.

The reaction of fused 1,2-dithiole-3-thione **9** with a number of symmetric diarylnitrilimines **81** afforded 1,3,4-thiadiazolines **82** in moderate to high yields [92]. Obviously, the reaction started with the formation of spiro-1,2-dithiolo-1,3,4-thiadiazoles **83**. The electron-withdrawing effect of the trigonal nitrogen atom of the thiadiazole ring can result in the opening of the 1,2-dithiole ring to give intermediate **84**, which after the extrusion of a sulfur atom gave the final product **82** (Scheme 52).

**Scheme 53:**
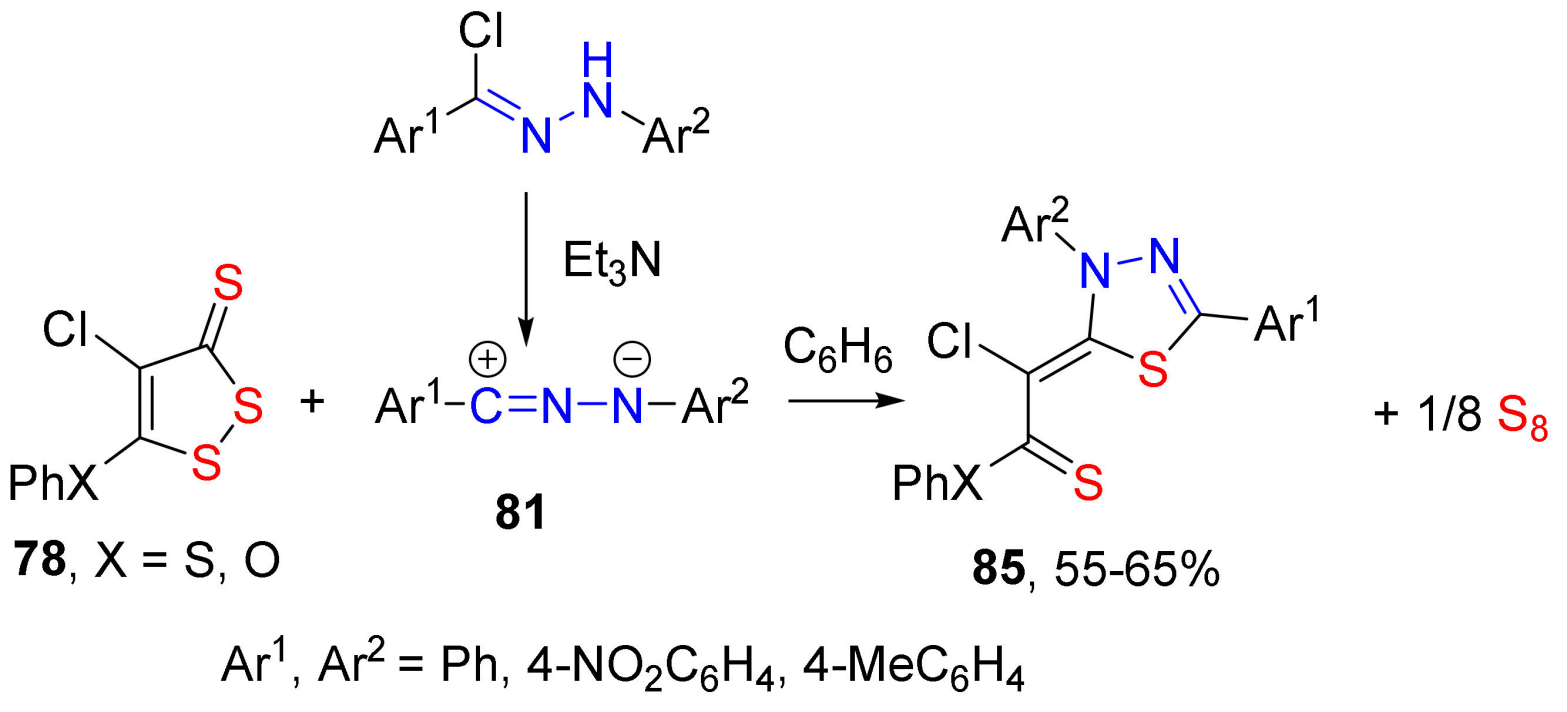
Reaction of 3*H*-1,2-dithiole-3-thiones **61** with diarylnitrilimines **64**.

5-Phenylthio- and 5-phenoxy-4-chloro-1,2-dithiole-3-thiones **85** reacted smoothly with nitrilimines **81** to give thiadiazoles **86** in good yields (Scheme 53). These conditions were used with a number of nitrilimines; thiadiazoles **85** were obtained in all cases in moderate yields [94]. It should be noted that elemental sulfur was isolated from these reactions in almost quantitative yields.

### 3.5. Recyclization Reactions

**Scheme 54:**
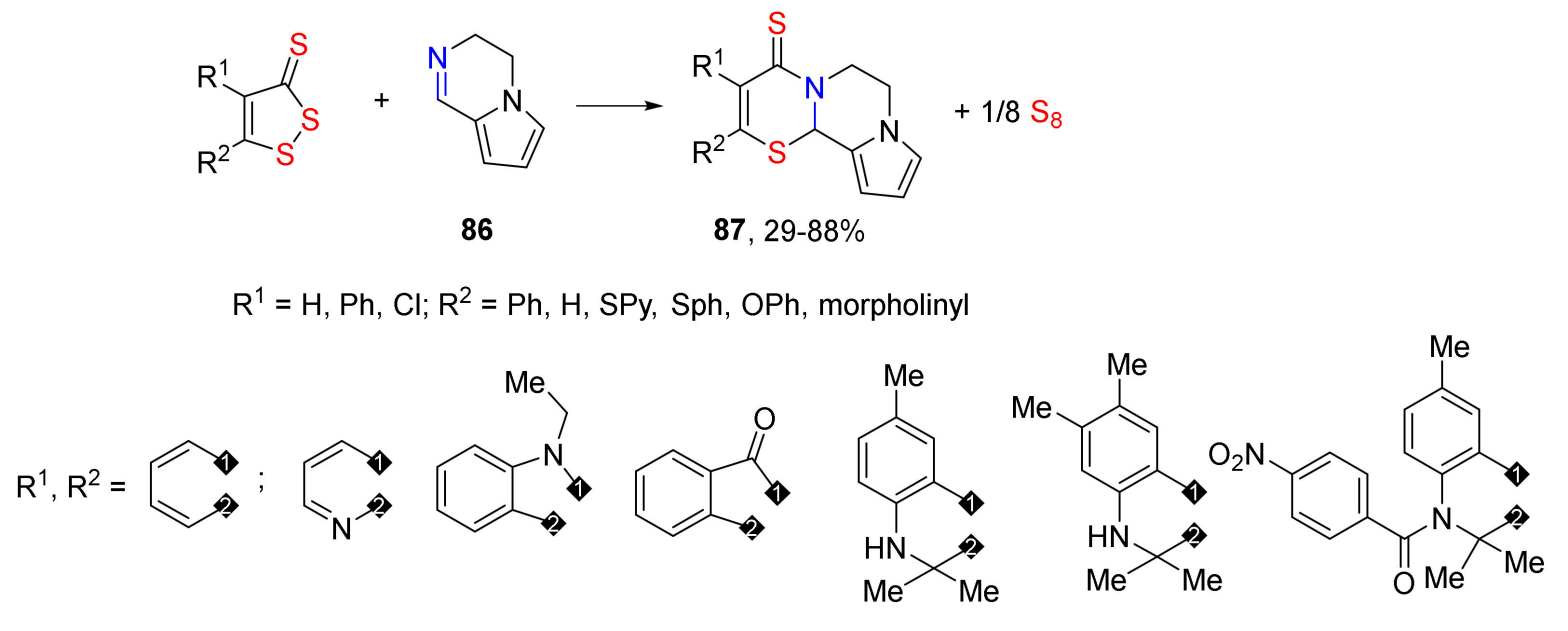
Reaction of 3*H*-1,2-dithiole-3-thiones with 3,4-dihydropyrrolo[1,2-*a*]pyrazine **69**.

Various monocyclic and fused 3*H*-1,2-dithiole-3-thiones can undergo a carbon-nitrogen or carbon-carbon bond insertion reaction, usually with extrusion of a sulfur atom. Neat treatment of an excess 3,4-dihydropyrrolo[1,2-*a*]pyrazine **86** with 3*H*-benzo[*c*][1,2]dithiole-3-thione at room temperature gave pyrazino[2,1-*b*][1,3]thiazine **87** in 64% yield [95,96]. Although this reaction requires a large excess of pyrazine **86**, unreacted dihydropyrrolopyrazine **86** was fully recovered from the reaction mixture. All attempts to improve the reaction procedure, such as heating equimolar quantities of both reagents under reflux conditions in various solvents (benzene, MeCN, pyridine or in DMSO, sulfolane or DMF at 95 °C) or treatment of a neat mixture of these reagents with Et_3_N under reflux for two days, failed. The scope of this one-pot reaction was studied: the yields of 1,3-thiazine-4-thiones **87** strongly depended on the structure of fused and monocyclic 1,2-dithiole-3-thiones (Scheme 54). The most reactive 1,2-dithiole-3-thiones fused with electron-withdrawing cycles (pyridine and dihydroindenone) reacted relatively quickly, while monocyclic ones required prolonged stirring and failed to completely consume the dithiolethiones even in 60 days. Finally, the S2 atom in both monocyclic and fused 1,2-dithioles is selectively replaced by the aminomethylene group to afford fused rigid six-membered 1,3-thiazines **87**.

**Scheme 55:**
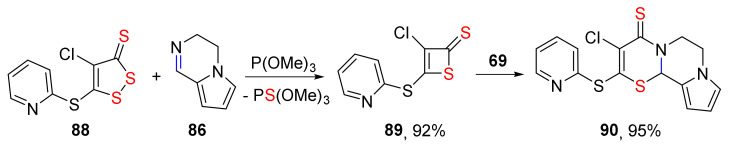
Isolation of intermediate thiete-2-thione **89**.

The reaction of monocyclic 1,2-dithiole-3-thione **88** with 3,4-dihydropyrrolo[1,2-*a*]pyrazine **69** in the presence of a sulfur extrusion agent (trimethylphosphite) afforded an intermediate product, four-membered thiete-2-thione **89**, in an excellent yield (92%) [97]. Subsequent treatment of thiete **89** with dihydropyrrolopyrazine **86** (1 equiv) gave fused 1,3-thiazine-4-thione **90** in 95% yield (Scheme 55). Two possible mechanisms for this reaction were suggested.

**Scheme 56:**
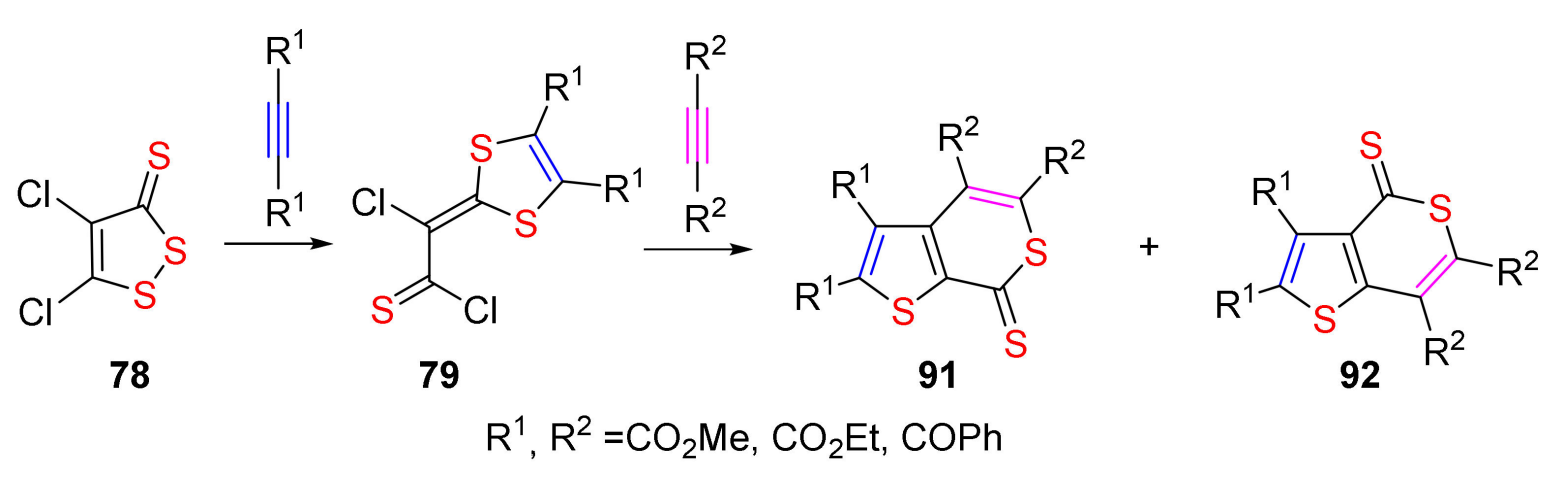
Synthesis of thienothiopyranethiones **91** and **92**.

Surprisingly, the thiopyranthione ring is formed as a result of the reactions of 1,2-dithiole-3-thiones with completely different reagents, namely, DMAD and sodium sulfide. Treatment of 4,5-dichloro-3*H*-1,2-dithiole-3-thione **78** (R = Cl) with excess DMAD in xylene, first at room temperature and then under reflux conditions, gave thienothiopyranethione isomers **91** and **92** in moderate yields [80]. Thus, the reaction occurs in a completely different way than in the case of 1,2-dithiole-3-thiones that do not contain two chlorine atoms as substituents (see Section 2.1): when two DMAD molecules are added, two chlorine atoms are removed (Scheme 56). As expected, the first step involved the addition of the first DMAD molecule to give thioacyl chloride **79**. The latter reacts with the second DMAD molecule with a rearrangement to afford thienothiopyranethione isomers **91** and **92**.

To study the mechanism of formation of products **91** and **92**, the behavior of three intermediate 1,3-dithiols **79** with three alkynes, i.e., DMAD, acetylene dicarboxylic acid diethyl ester (DEAD), and dibenzoylacetylene (DBA), was studied. In each case, a pair of isomeric thienothiopyranethions **91** and **92** was isolated, and it was unexpectedly found by means of XRD that in all cases the alkyne moiety of **79** appeared in the thiophene ring, while the second alkyne molecule was found in thiopyranes **91** and **92**.

The possible pathways for the formation of thiopyranes **91** and **92** were suggested. The authors believe that the main reason for these rearrangements lies in the presence of an intramolecular S...S bond in molecule **62** (proved by X-ray diffraction analysis), which can reduce the ability of these compounds to undergo Diels-Alder cycloaddition and, at high temperatures, leads to a chain of reactions involving the opening and closure of various heterocyclic rings. The formation of the final heteroaromatic products **91** and **92** can occur upon elimination of a chlorine molecule from the dichloro adducts. The presence of a chlorine molecule in the reaction mixtures was proved by isolation of the same xylene chlorination products, like in the case where xylene was refluxed with chlorine.

**Scheme 57:**
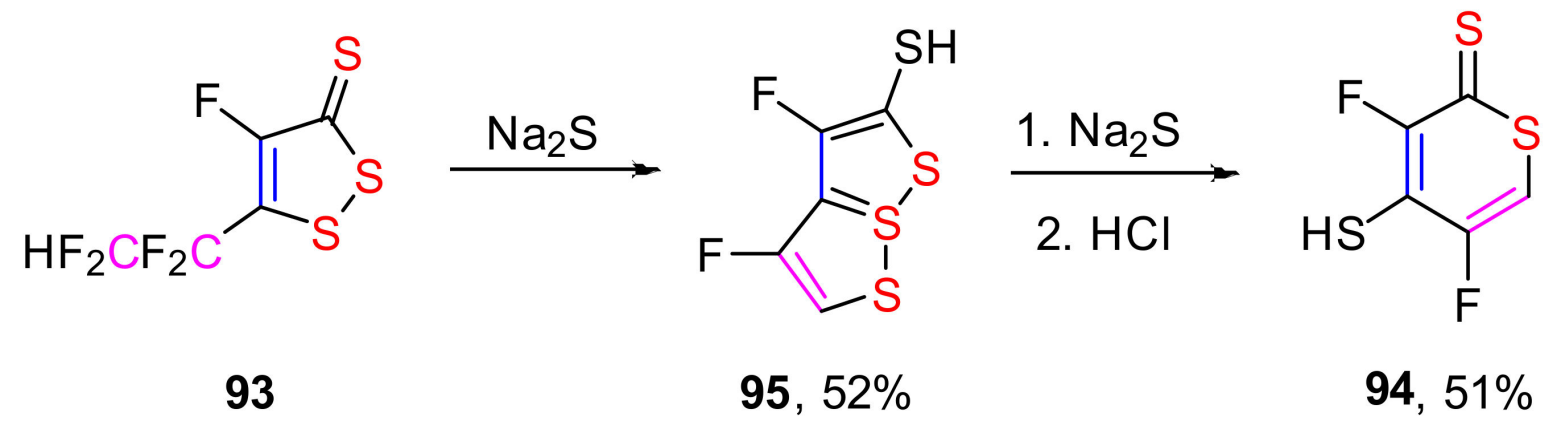
Synthesis of thiopyranthione **94**.

Yet another example of the formation of a thiopyrane ring from 1,2-dithiole-3-thione was observed in the reduction of 4-fluoro-5-(1,1,2,2-tetrafluoroethyl)-1,2-dithiole-3-thione **93** with sodium sulfide [98]. It was found that refluxing dithiolethione **93** with excess sodium sulfide gave thiopyranthione **94** (Scheme 57). Trithiapentalene **95** was isolated by using an equimolar ratio of the reagents under the same conditions. The formation of the trithiapentalene system was explained as a sequential process of reduction and substitution of fluorine atoms under the action of a sulfide anion followed by its addition and elimination of the vinyl sulfur atom, while the formation of the thiopyrane system was followed by a rearrangement of the trithiapentalene system.

**Scheme 58:**
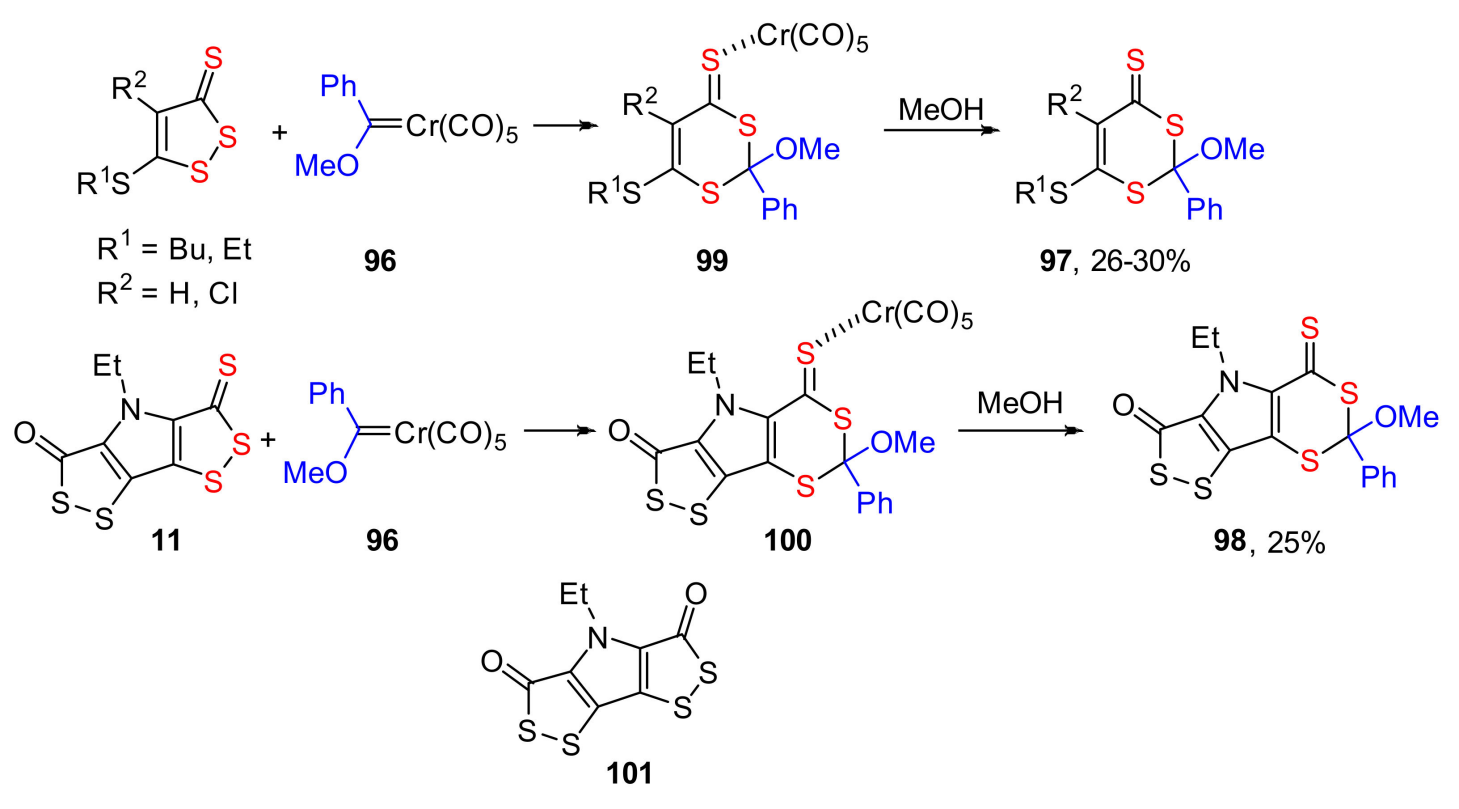
Reaction of 3*H*-1,2-dithiole-3-thiones with Fischer’s carbene complex **96**.

A number of 1,2-dithiole-3-thiones, both monocyclic and fused **11**, were studied in the reaction with Fischer’s carbene complex **96** [99]. Insertion of a carbene moiety into the S...S bond resulted in 1,3-dithiine-4-thiones **97** and **98**, which were isolated after treatment of intermediate complexes **99** and **100** with methanol (Scheme 58). It is noteworthy that ketone **101** does not react with the Fischer carbene complex **96**, thus the presence of a thiocarbonyl group is the main condition for incorporation into the 1,2-dithiole ring. This is confirmed by the fact that the reaction of tricyclic compound **11** containing 1,2-dithiole-3-thione and 1,2-dithiol-3-one rings in its molecule with Fischer’s carbene complex **96** gave only a product of insertion in the 1,2-dithiole-3-thione ring **98**. The authors suggest that this selectivity indicates that the reaction begins with the attack of the nucleophilic thiocarbonyl group on the electrophilic carbenium atom of the Fischer carbene complex. Interestingly, in the first report on the reaction of 1,2-dithiole-3-thiones with Fischer carbene complexes, the authors argued that the incorporation of the carbene ligand occurs via the C3-C4 bond of the dithiole ring [100]. In a subsequent paper [99], an X-ray diffraction analysis of the compounds obtained was carried out and the initial data were corrected.

**Scheme 59:**
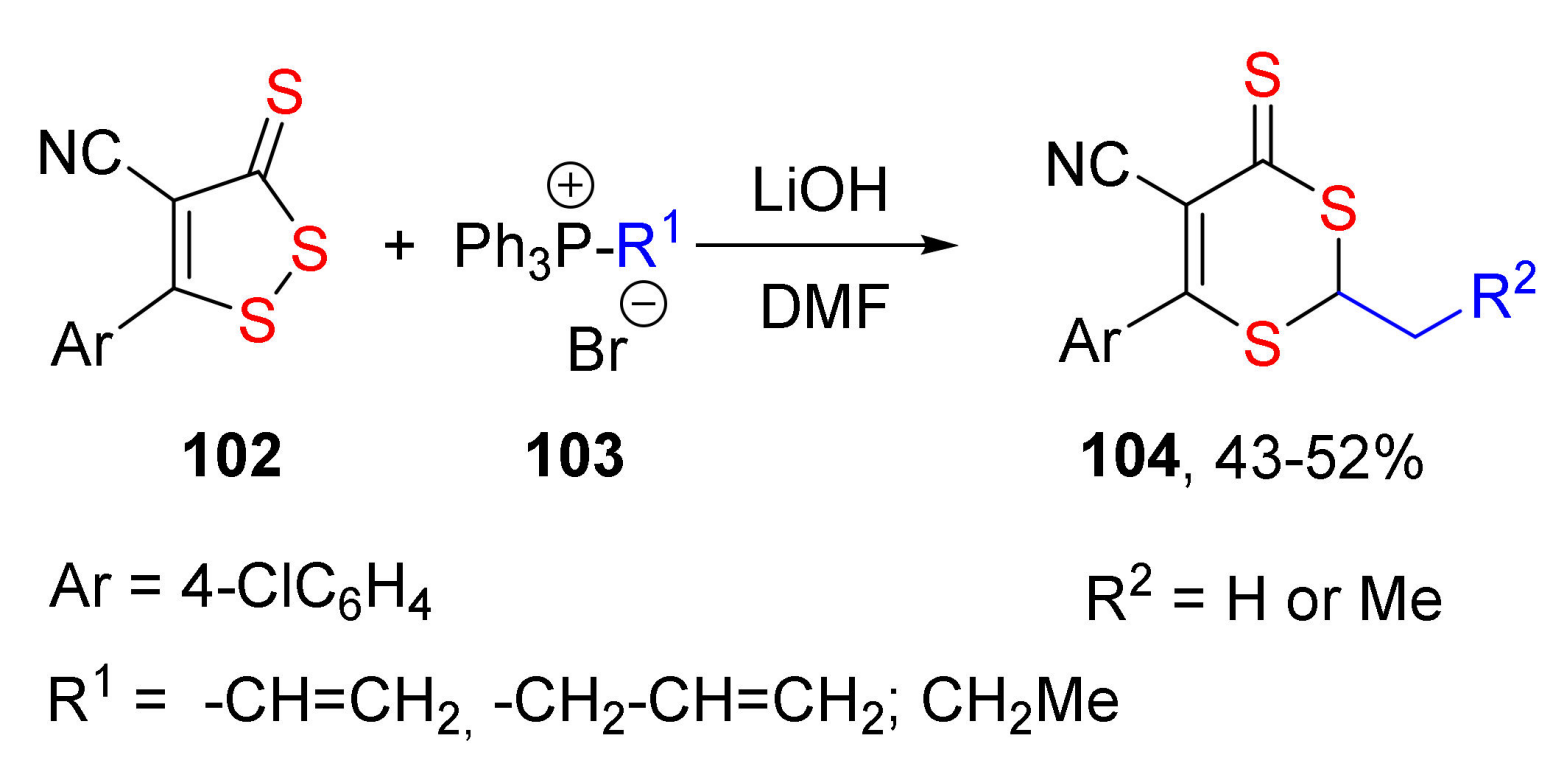
Synthesis of 1,3-dithiine 4-thione **104**.

The possibility of insertion of a carbon-containing particle was also demonstrated in the reaction of 1,2-dithiole-3-thione **102** with phosphonium ylides [101]. After treatment of thione **102** with phosphonium salts **103** in the presence of lithium hydroxide in DMF, 1,3-dithiine 4-thione **104** was isolated as the main product (Scheme 59).

**Scheme 60:**
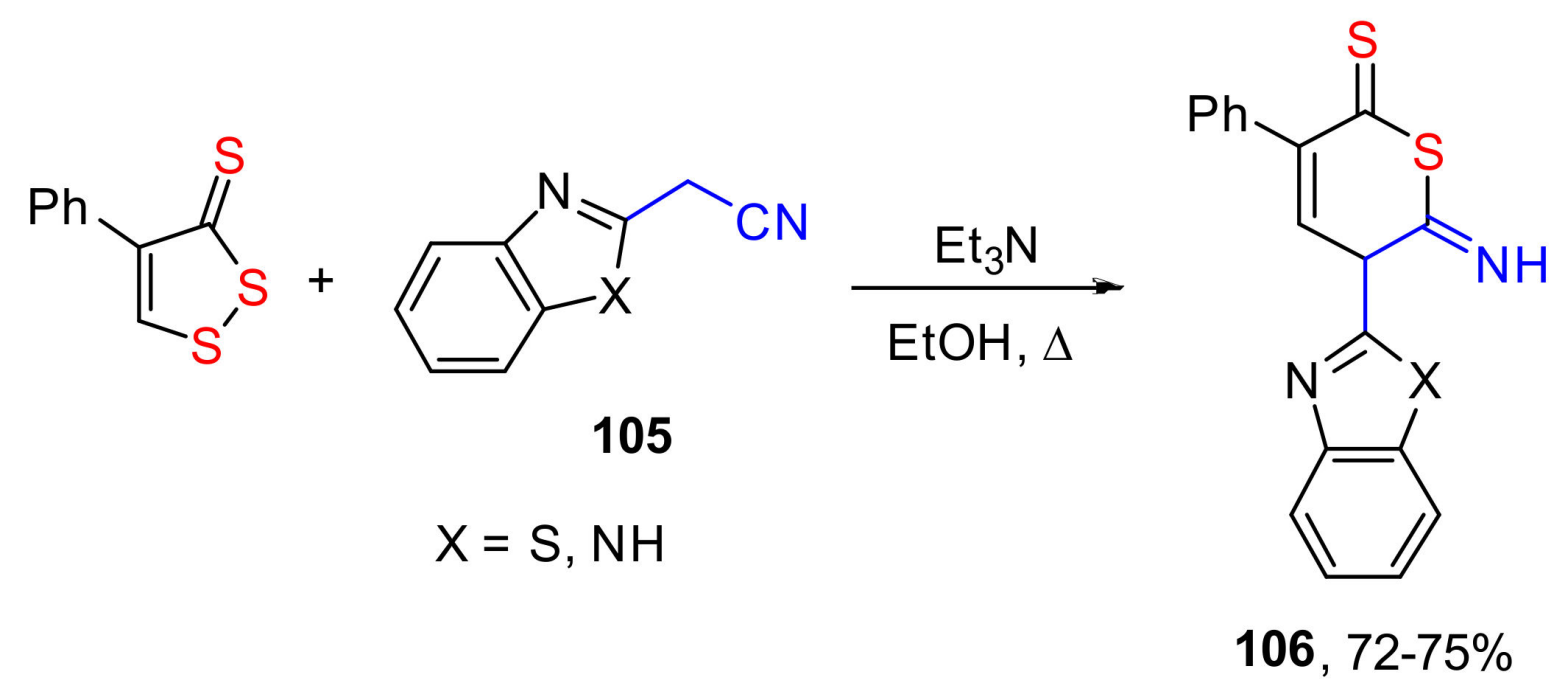
Reaction of 4-phenyl-3*H*-1,2-dithiole-3-thione with 2-cyanomethyl-benzothiazole and -benzimidazole **105**.

Egyptian authors have shown that in the reaction of 4-phenyl-1,2-dithiole-3-thione with nitriles containing a reactive methylene group and with α,β-unsaturated nitriles, the sulfur atom is replaced by a C-C bond, while the carbon atom of the nitrile group and the adjacent carbon atom are included in the new cycle [102]. Thus, the reaction of 4-phenyl-1,2-dithiole-3-thione with 2-cyanomethyl-benzothiazole and -benzimidazole **105** in the presence of triethylamine gave products that the authors concluded to have the structure of imino-2*H*-thiopyran-2-thiones **106** (Scheme 60).

**Scheme 61:**
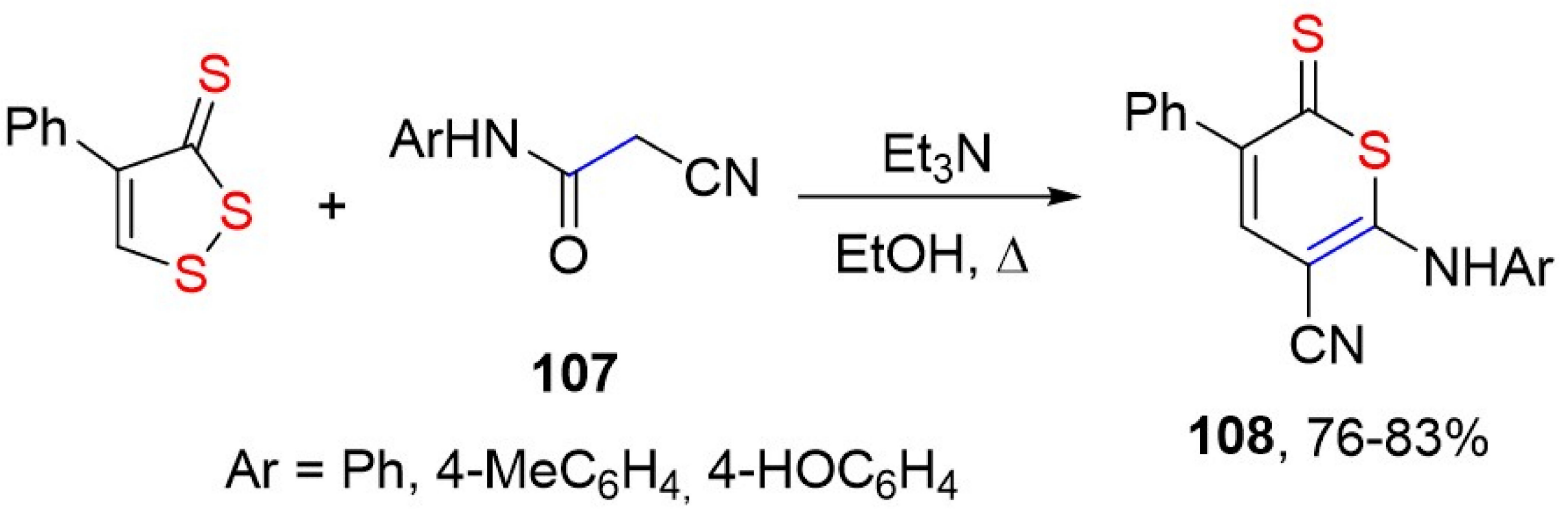
Synthesis of 2*H*-thiopyran-2-thiones **108**.

At the same time, in the case of the reaction of acetonitriles **107**, which are structurally similar to compounds **88** under similar conditions, the amide group is involved in the reaction rather than the nitrile group, as might be expected from the previous scheme [102]. As a result, 2*H*-thiopyran-2-thiones **108** were isolated in high yields (Scheme 61).

**Scheme 62:**
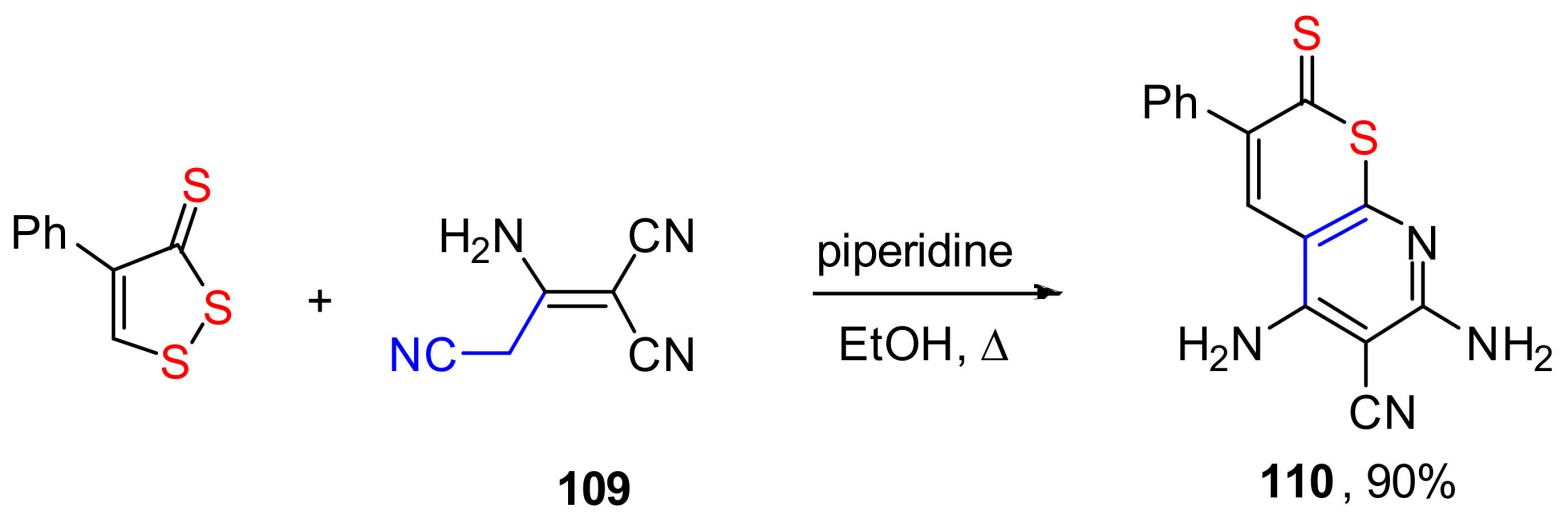
Synthesis of 2*H*-thiopyrano[2,3-*b*]pyridine-2-thione **110**.

Interestingly, the acetonitrile group attached to the double bond in compound **109** reacted in a similar manner to give substituted bicyclic 2*H*-thiopyrano[2,3-*b*]pyridine-2-thione **110** (Scheme 62) [103].

**Scheme 63:**
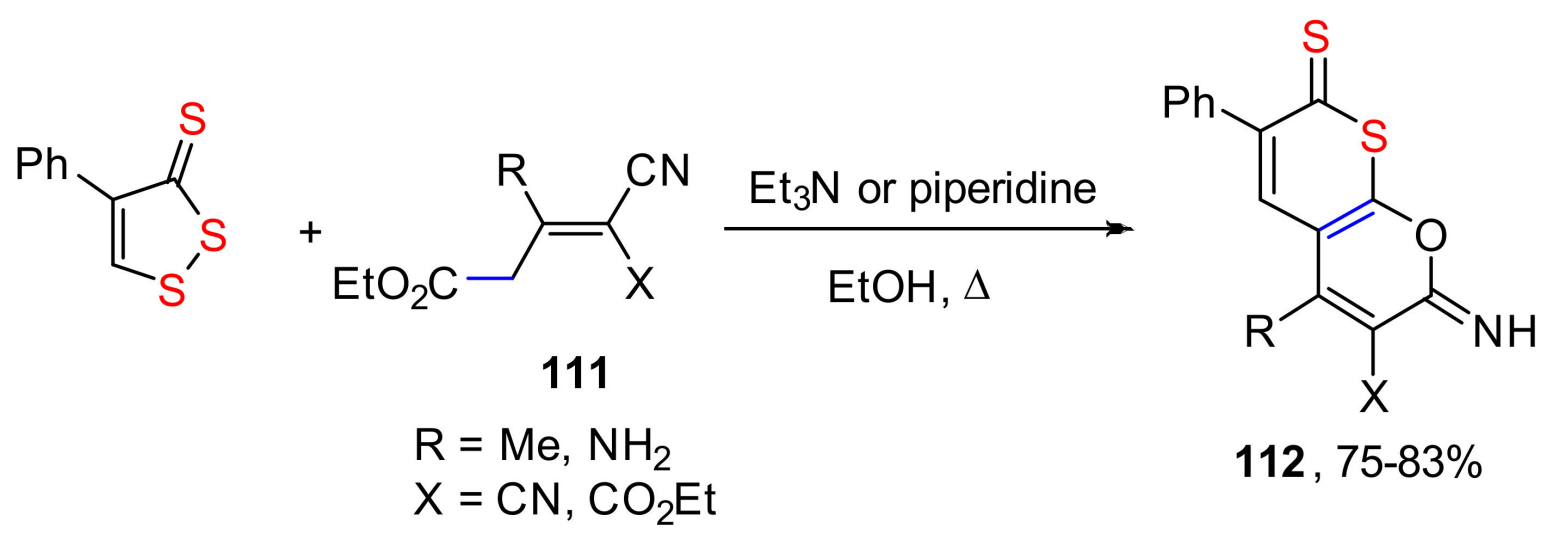
Synthesis of 2-imino-2*H*,7*H*-thiopyrano[2,3-*b*]pyran-7-thiones **112**.

If the acetonitrile group was replaced with an acetic acid ester group as in compounds **111**, then the ester group was involved in the reaction and derivatives of 2-imino-2*H*,7*H*-thiopyrano[2,3-*b*]pyran-7-thione **112** formed (Scheme 63) [102,103].

**Scheme 64:**
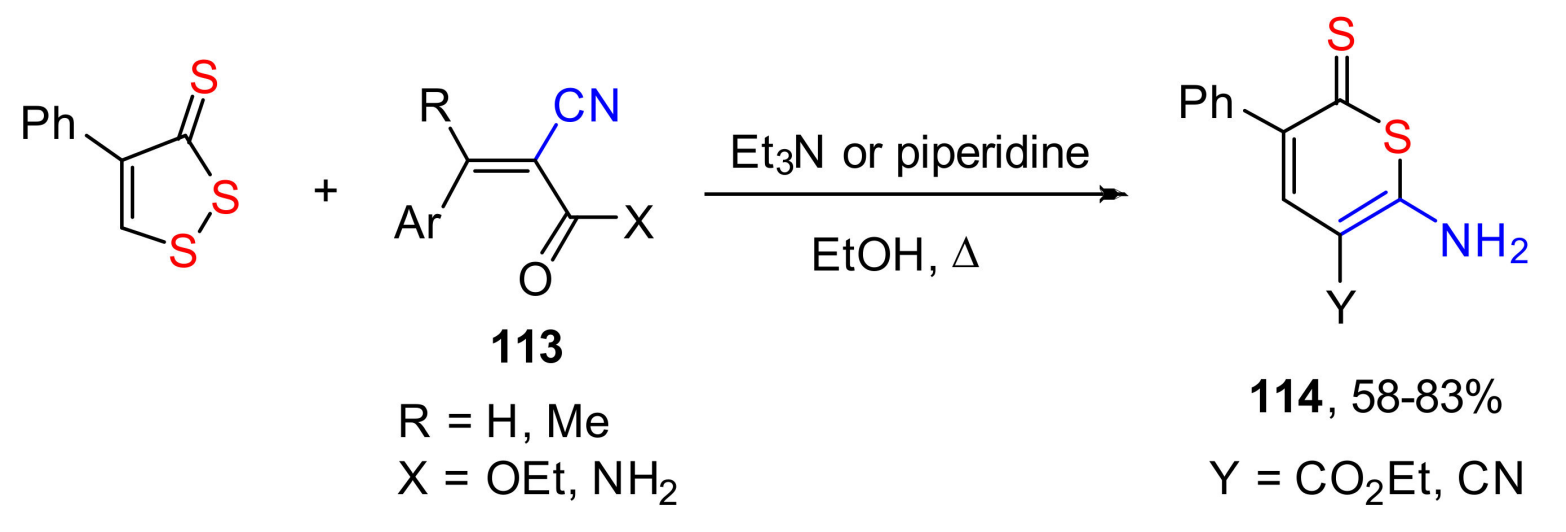
Synthesis of 2*H*-thiopyran-2-thiones **114**.

The reaction with conjugated nitriles **113** also involved the nitrile group and resulted in 2*H*-thiopyran-2-thiones **114** [102,104]. The reaction conditions are the same as in all previous transformations, i.e., refluxing in ethyl alcohol in the presence of a base (piperidine or triethylamine) (Scheme 64). If an acetamide group is present in a conjugated nitrile, then it is dehydrated into a nitrile group [102].

In all the cases described in this section, it is assumed that the cyclic sulfur atom at position 1 undergoes replacement. Unfortunately, the structure of the compounds synthesized is proven only by elemental analysis, IR and NMR spectra, which does not allow their structures to be identified unambiguously. The other drawback of these studies is that the fate of the sulfur atom was not clarified in any of the cases.

### 3.6. Opening of the 1,2-dithiole Ring

**Scheme 65:**
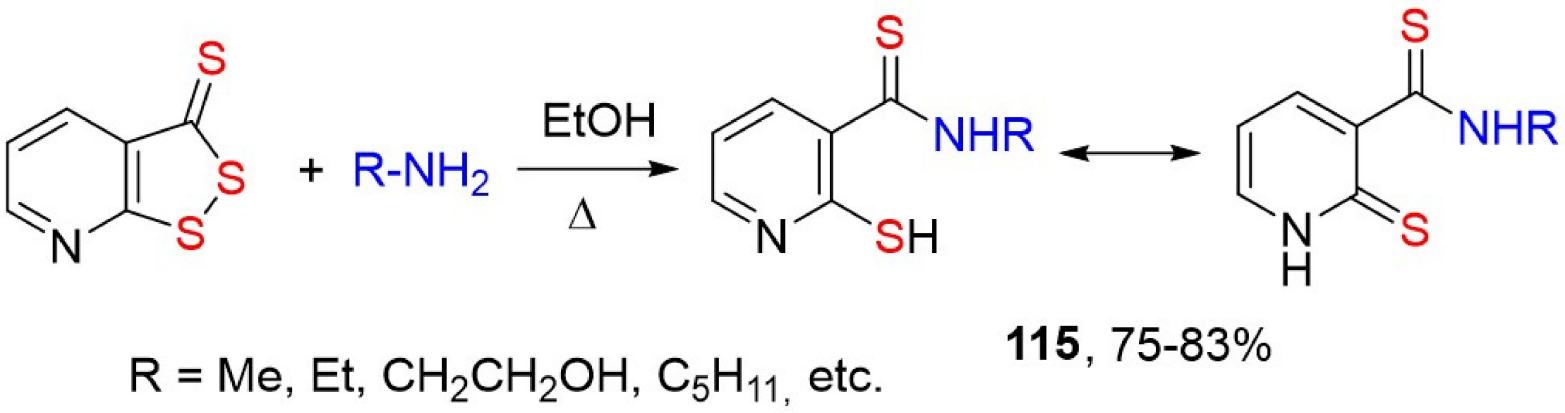
Reaction of 3*H*-[1,2]dithiolo[3,4-*b*]pyridine-3-thione with amines.

Opening of the 1,2-dithiole ring can be achieved by the reaction of 1,2-dithiole-3-thiones with amines [105,106]. It was found that the reaction of 3*H*-[1,2]dithiolo[3,4-*b*]pyridine-3-thione with aliphatic amines gave compounds containing thiol and carbothioamide groups at the *ortho*-positions, *N*-alkyl-1,2-dihydro-2-thioxo-3-pyridocarbothioamides **115**, in high yields (Scheme 65). Nothing is reported about the fate of the removed sulfur atom.

**Scheme 66:**
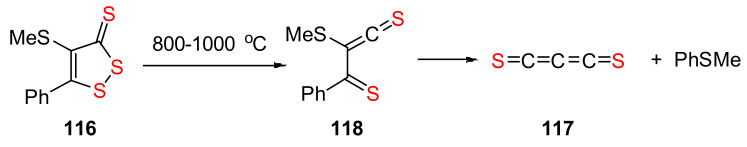
Vacuum pyrolysis of dithiolethione **116**.

4-(Methylthio)-5-phenyl-3H-1,2-dithiole-3-thione **116** was subjected to vacuum pyrolysis at 800–1000 °C [107]. The main product characterized in an argon matrix at 10 K was allene-1,3-dithione **116**. The mechanism of its formation included the generation of thioacylthioketene **118** from dithiolethione **117** followed by the phenyl group shift to the sulfur atom of the thioalkyl group and elimination of *S*-methylthiophenol (Scheme 66).

**Scheme 67:**
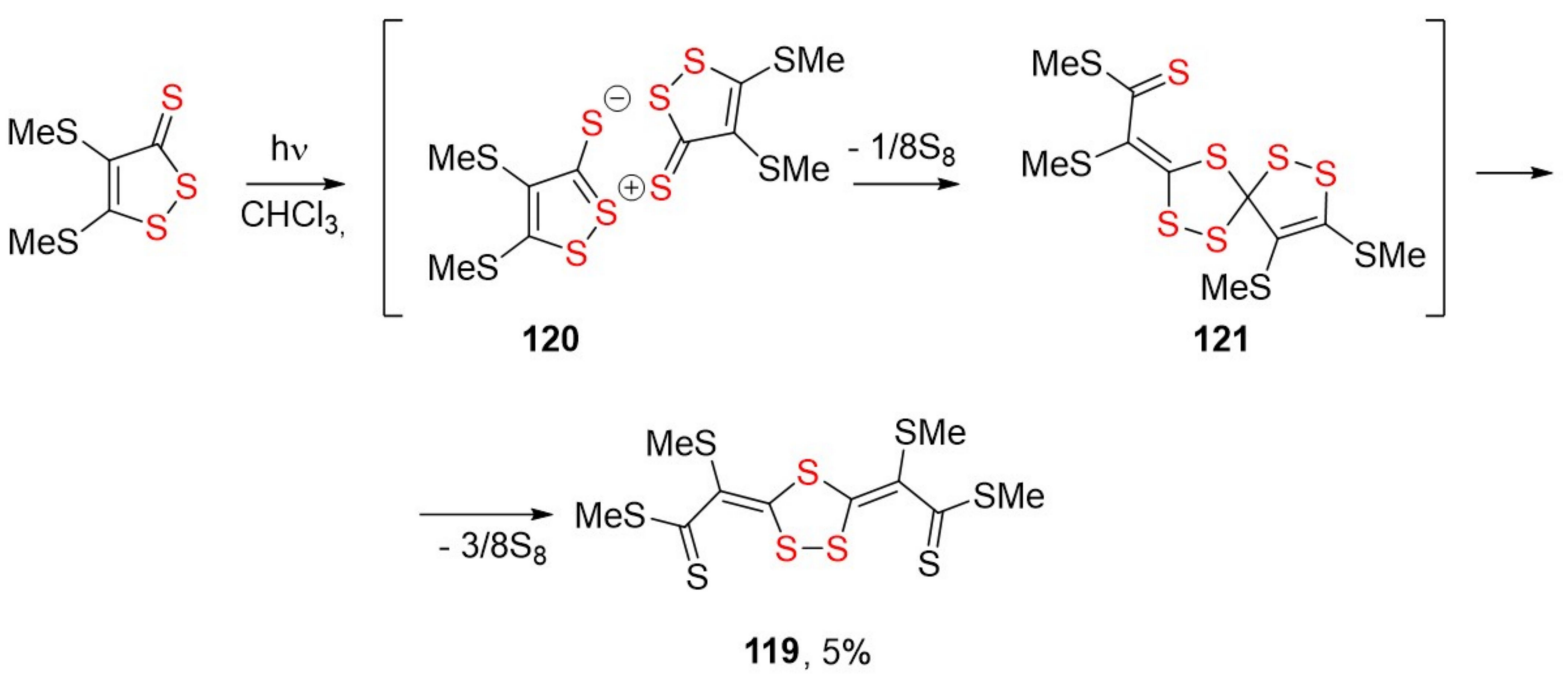
Irradiation of 4,5-bis(methylthio)-3*H*-1,2-dithiole-3-thione.

4,5-Bis(methylthio)-3*H*-1,2-dithiole-3-thione was irradiated with a UV lamp for 24 h in chloroform [108]. Though the reaction was performed for a long time, it occurred by 20% only (80% of the starting compound was recovered) and the only product, trithiolane **119**, was isolated in 5% yield (Scheme 67). The authors suggested a mechanism for this unexpected transformation, including the dimerization of dithiolethione in the canonical zwitterionic form **120** to the intermediate spiro compound **121**, which is then rearranged with extrusion of the sulfur atom to give the final compound **119**.

**Scheme 68:**
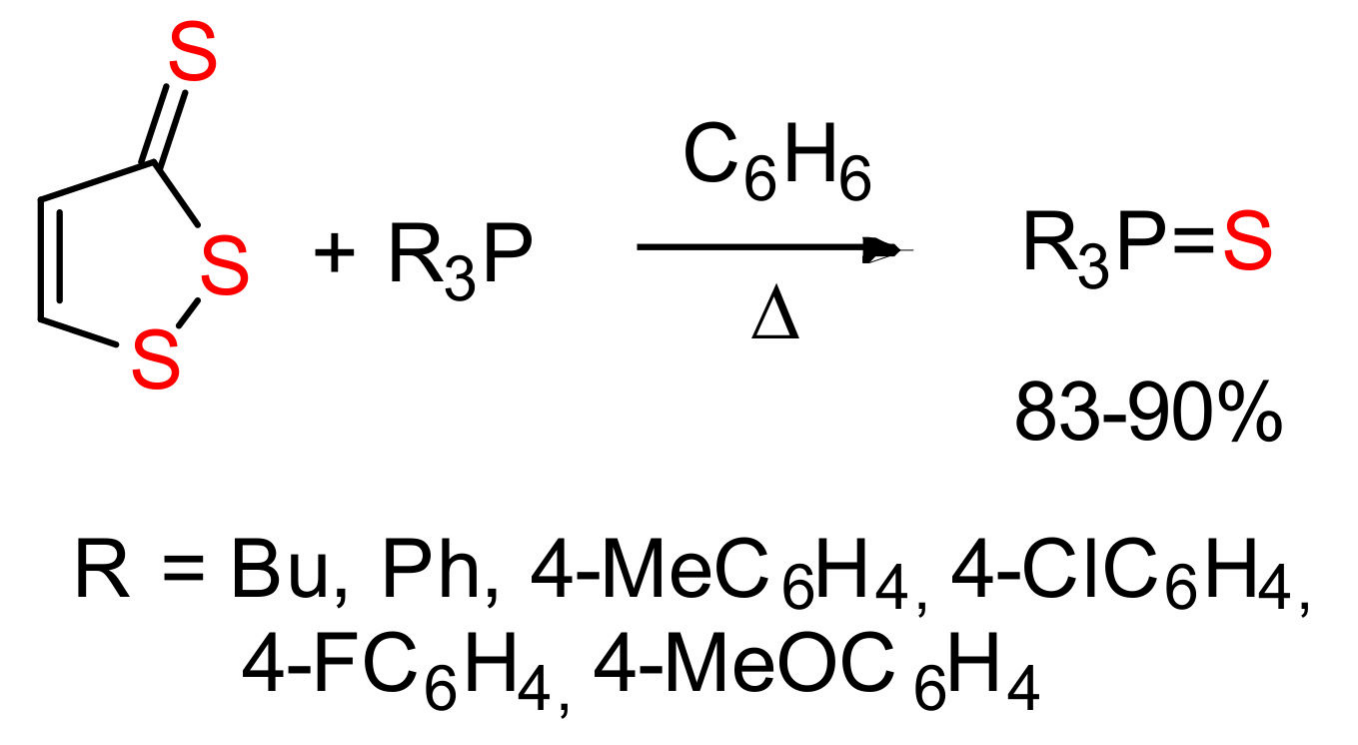
Reaction of 3*H*-1,2-dithiole-3-thione with Ph_3_P.

Unsubstituted 3*H*-1,2-dithiole-3-thione was shown to be an excellent sulfurating agent that converts trialkyl- and triarylphosphines to the corresponding phosphine sulfides in high yields [109] (Scheme 68).

### 3.7. Oxidation of 1,2-dithiole-3-thiones

**Scheme 69:**
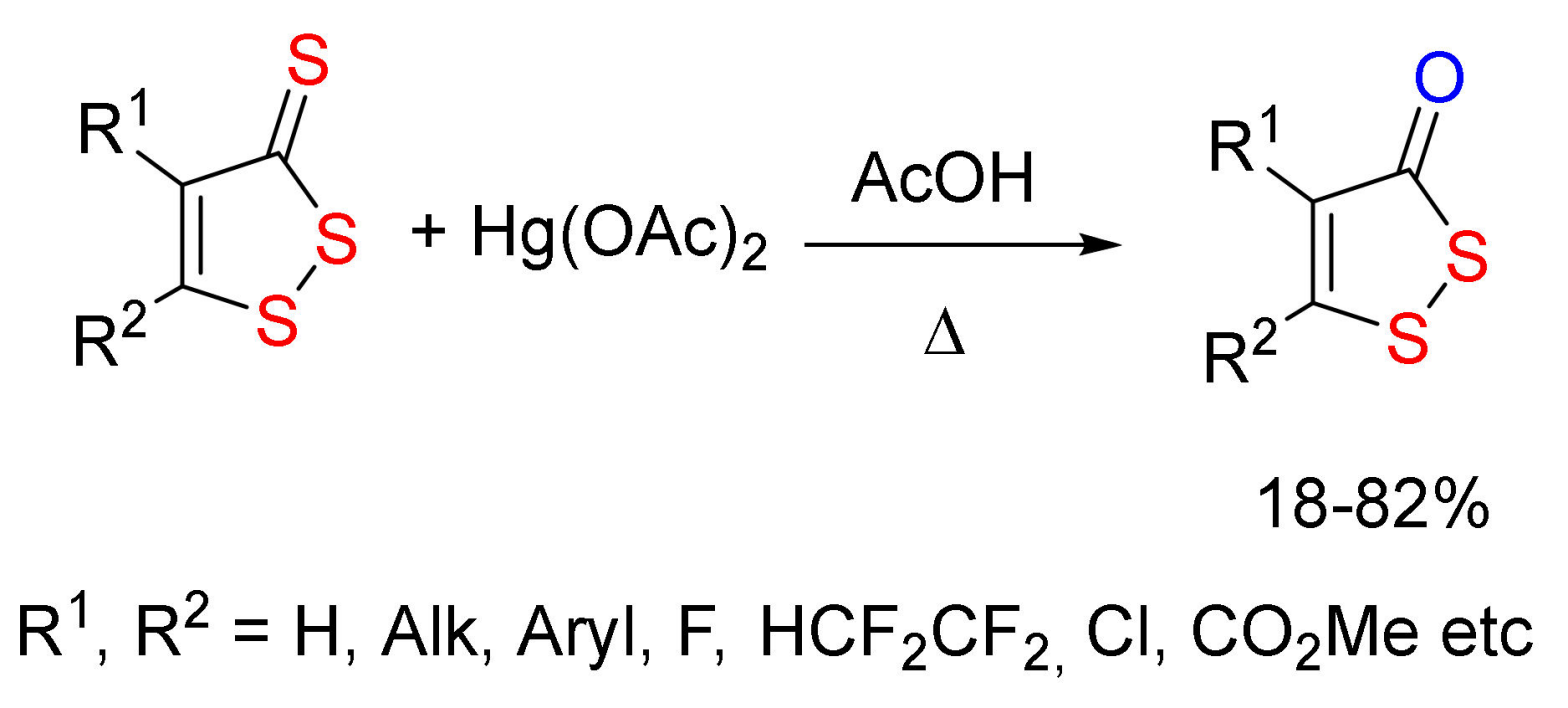
Oxidation of monocyclic 3*H*-1,2-dithiole-3-thiones with Hg(OAc)_2_.

Mercury acetate, a readily available and inexpensive reagent, is the most commonly used oxidant for 1,2-dithiole-3-thiones; chloroform, acetic acid or their mixtures are used as solvents. It follows from an analysis of literature data that the solubility of both 1,2-dithiole-3-thione and mercury acetate can be the main criterion for the choice of the solvents. Heating of monocyclic 1,2-dithiole-3-thiones in acetic acid was used most often [110,111,112,113], however, the yields can vary widely from 18 to 82%, and no dependence of the yields on the nature of substituents at positions 4 and 5 of the heterocycle was found (Scheme 69).

**Scheme 70:**
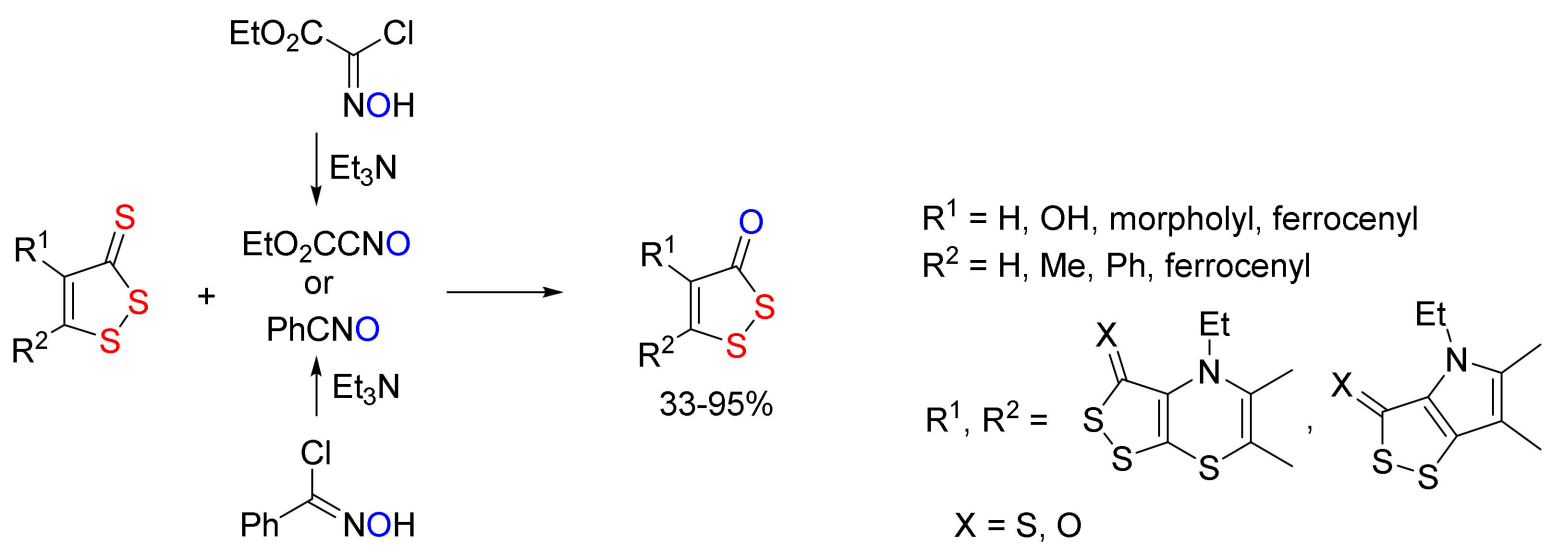
Oxidation of 3*H*-1,2-dithiole-3-thiones with nitrile oxides.

The reaction of 1,2-dithiole-3-thiones with nitrile oxides is yet another useful procedure. Nitrile oxides are unstable compounds that can readily dimerize to 1,2,5-oxadiazole 1-oxides (furoxans); therefore, they are prepared in situ by the reaction of hydroxamic acid chlorides with triethylamine (Scheme 70). Commercial hydroxamic acid chloride, viz., ethyl chloroximidoacetate [55], and phenylhydroxamic acid chloride that is easy to synthesize [114,115] were employed.

A comparison of two reagents, mercury acetate and nitrile oxide, was carried out in Ref. [55]; it was found that in all the cases studied, the yields of dithiolethiones were significantly higher if the second method was used.

Potassium permanganate was employed to convert 4,5-diaryl-1,2-dithiole-3-thiones [5,29] and 5-thienyl-1,2-dithiole-3-thione [37] to the corresponding dithiolones in good yields. Other oxidizing agents were used less frequently. Bismuth nitrate (Bi(NO_3_)_3_) was tested as an oxidant for 5-butylthio-1,2-dithiole-3-thione [110], however, the yield of 5-butylthio-1,2-dithiole-3-one was low (30%). Therefore, the authors of that study preferred other methods to convert the thione group in 1,2-dithiole-3-thione into a ketone group.

**Scheme 71:**
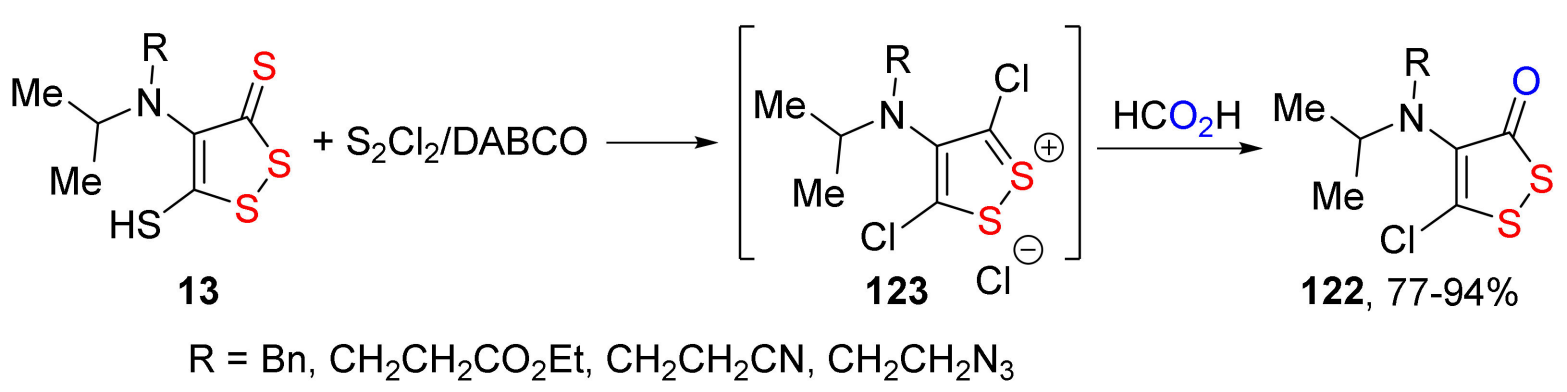
Reaction of mercaptodithiolethiones with S_2_Cl_2_ and HCO_2_H.

Mercaptodithiolethiones **13** were brought into the reaction with a mixture of S_2_Cl_2_ and DABCO under the conditions for the formation of tricyclic bis-dithiolothiazines **8** and **9** from substituted diisopropylamines [55]. However, contrary to expectations, 5-chlordithiole-3-ones **122** formed in high yields in all the cases (Scheme 71) [62]. It was assumed that the most likely precursor of **122** was dichlorodithiolium salts **123**, which reacted with HCO_2_H to give chloroketone **122**, as described previously [61]. Apparently, the formation of this salt occurred as a result of a two-fold attack by an electrophilic S_2_Cl_2_ on the thione group of **13**.

### 3.8. Synthesis of 1,2-dithiolium Salts

**Scheme 72:**
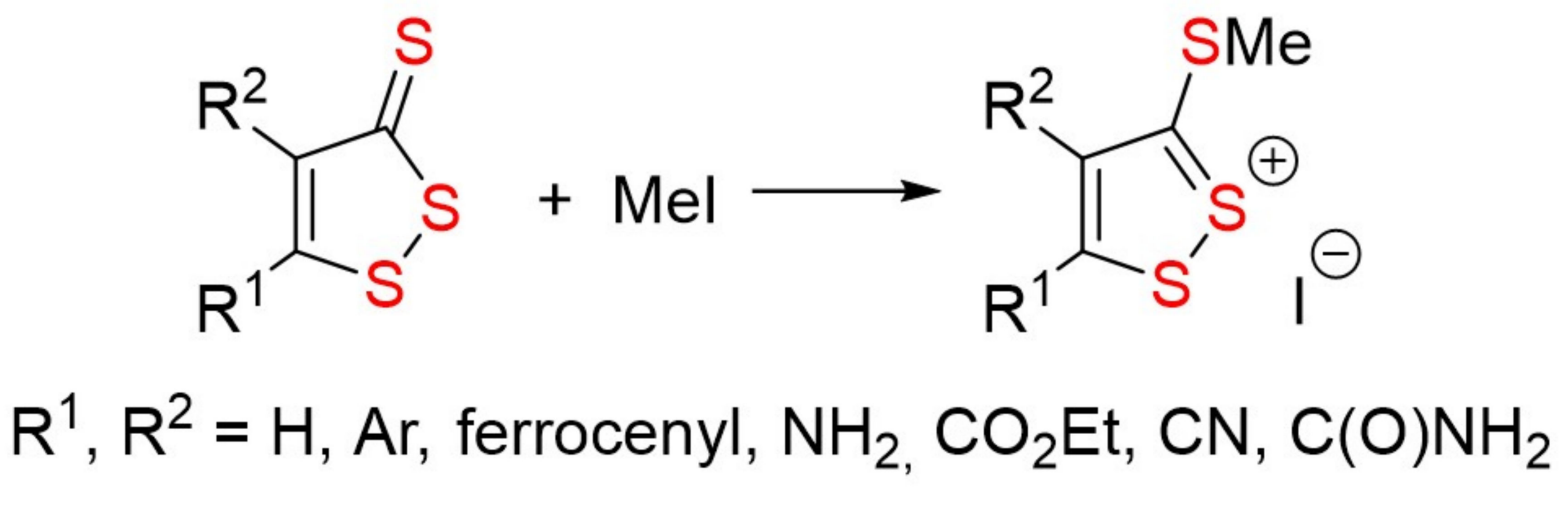
Alkylation of 1,2-dithiole-3-thiones.

There are two main methods for the formation of 1,2-dithiolium salts from 1,2-dithiole-3-thiones: i, alkylation with alkyl halides; ii, chlorination of 1,2-dithiole-3-thiones. Monocyclic 1,2-dithiole-3-thiones reacted with alkyl and benzyl iodides at room temperature (Scheme 72). The treatment with methyl iodide was carried out in acetone [114,116], in benzene [117] or in DMF for 1,2-dithiole-3-thiones containing ferrocenyl [114], aryl substituents [116,117], and functional (nitrile, ester and carboxamide) groups [118]. The yields of 1,2-dithiolium salts were usually high (70–90%), though they were low in some cases (down to 17%).

**Scheme 73:**
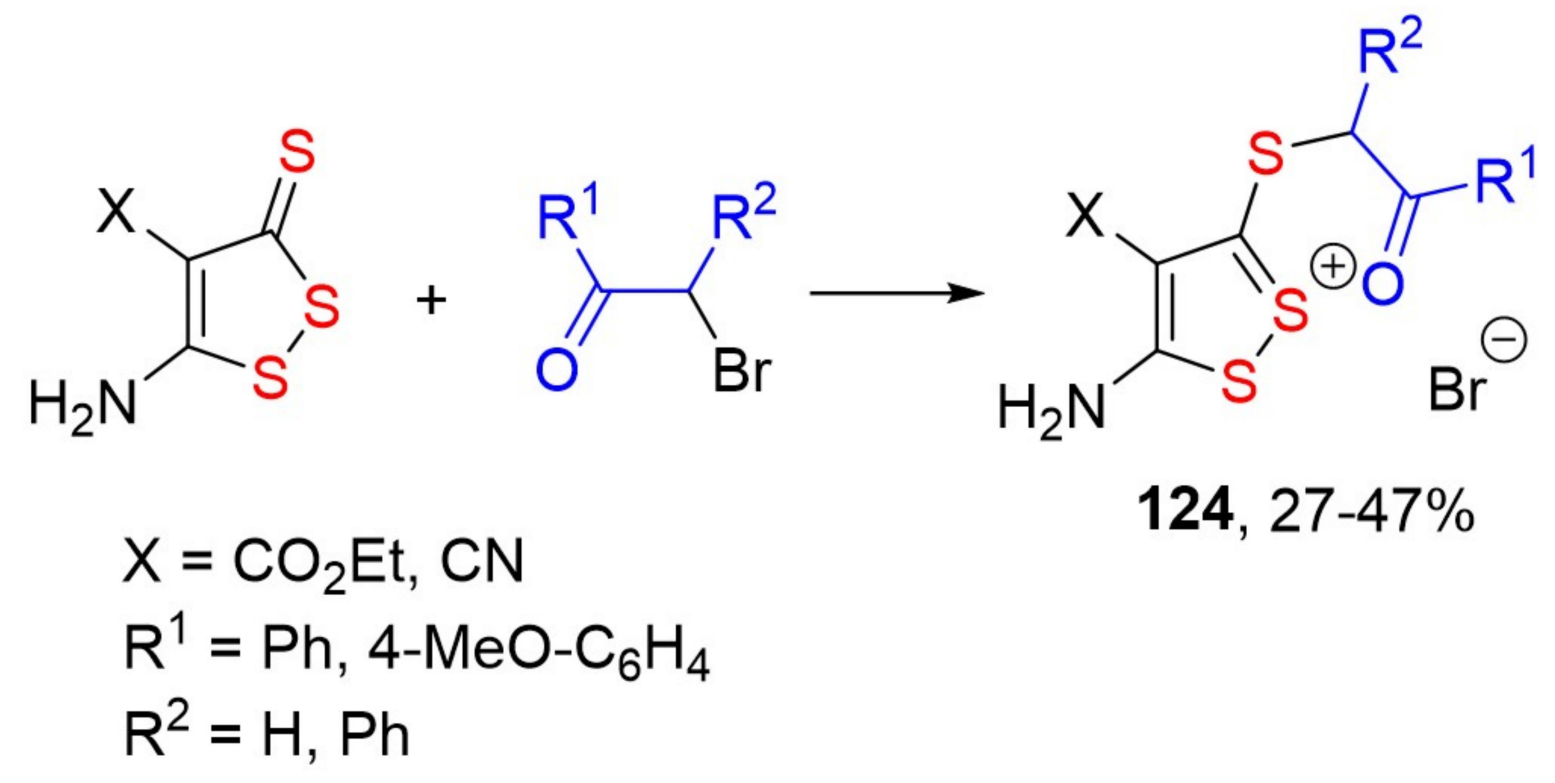
Synthesis of 1,2-dithiolium salts **124**.

Bromo derivatives activated by an α-carbonyl group were employed for the synthesis of 1,2-dithiolium salts less frequently [119]. The yields of salts **124** were as low as 27–47% (Scheme 73).

**Scheme 74:**
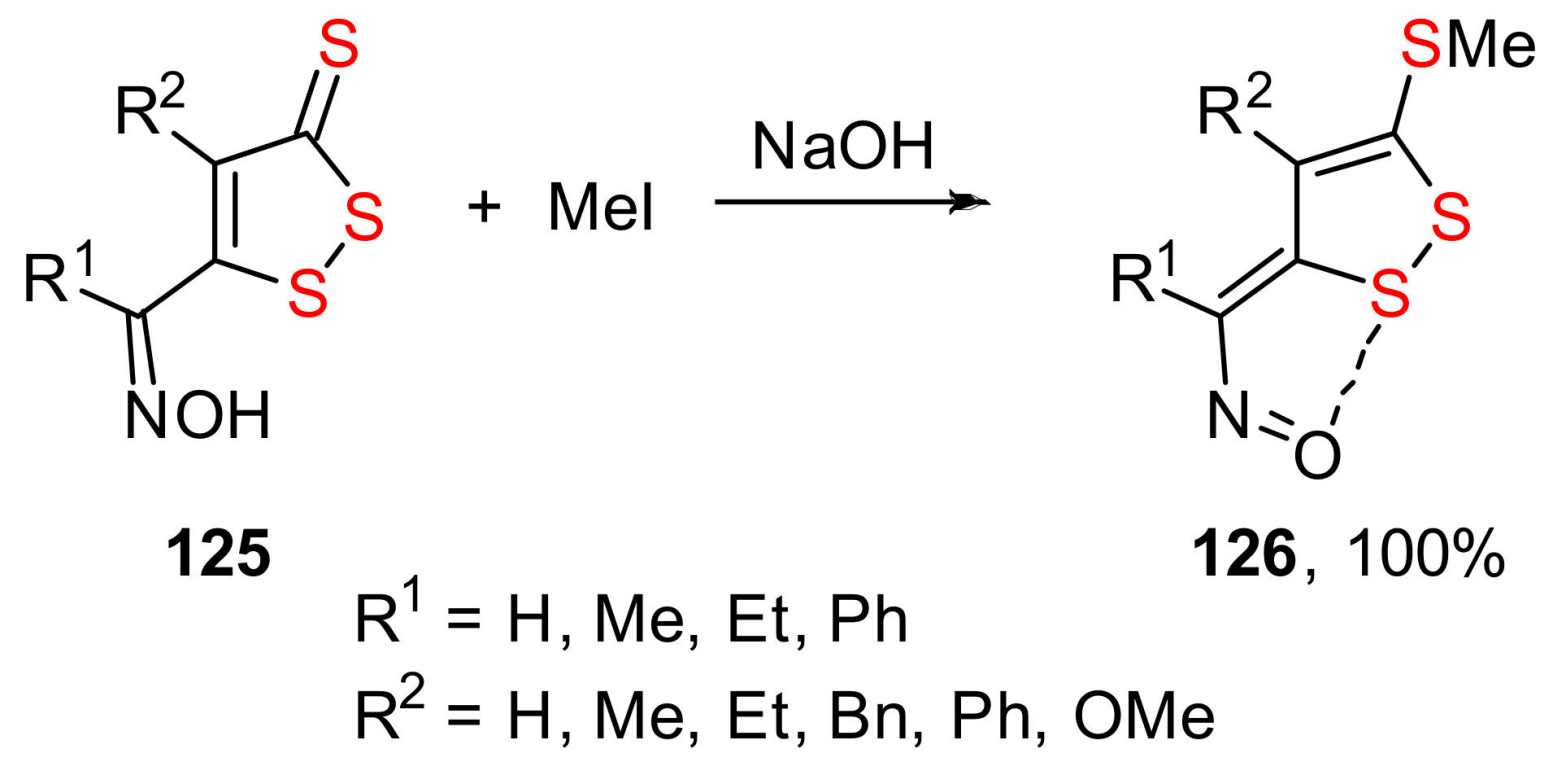
Reaction of 5-(hydroxylaminoalkyl)-1,2-dithiole-3-thiones **125** with methyl iodide.

The reaction of 5-(hydroxylaminoalkyl)-1,2-dithiole-3-thiones **125** with methyl iodide in water in the presence of sodium hydroxide involved, along with the alkylation of the exocyclic sulfur atom, also a rearrangement of bonds in the 1,2-dithiole ring and oxime group. Nitroso derivatives **126** were formed in quantitative yields (Scheme 74). Compounds **126** can be considered as heteropentalenes due to the strong interaction of the oxygen atom of the nitroso group and the sulfur atom of the cycle [120].

**Scheme 75:**
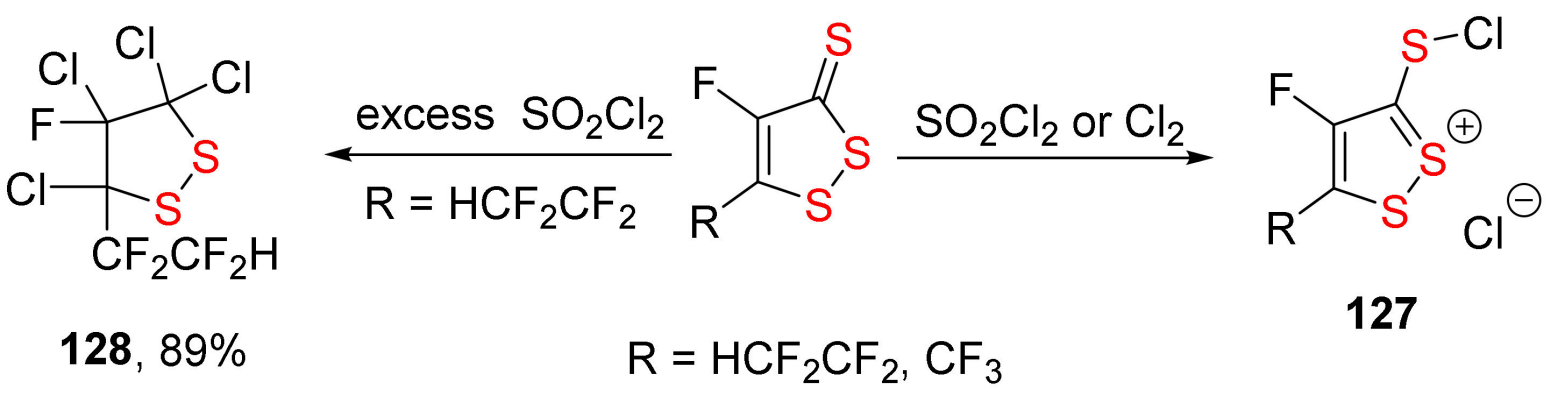
Chlorination of 1,2-dithiole-3-thiones.

Reactions of fluorinated 1,2-dithiole-3-thiones with chlorinating agents (chlorine or sulfuryl chloride) resulted in the chlorination of exocyclic sulfur atom **127** [111,121]. These compounds were typically used in situ in further reactions. However, in one case, product **127** was isolated and characterized (Scheme 75). If an excess of the chlorinating agent (SO_2_Cl_2_) was used in the reaction with fluorinated 1,2-dithiole-3-thione, the thione group was replaced by two chlorine atoms and the double bond of the 1,2-dithiole ring was also halogenated [111].

**Scheme 76:**
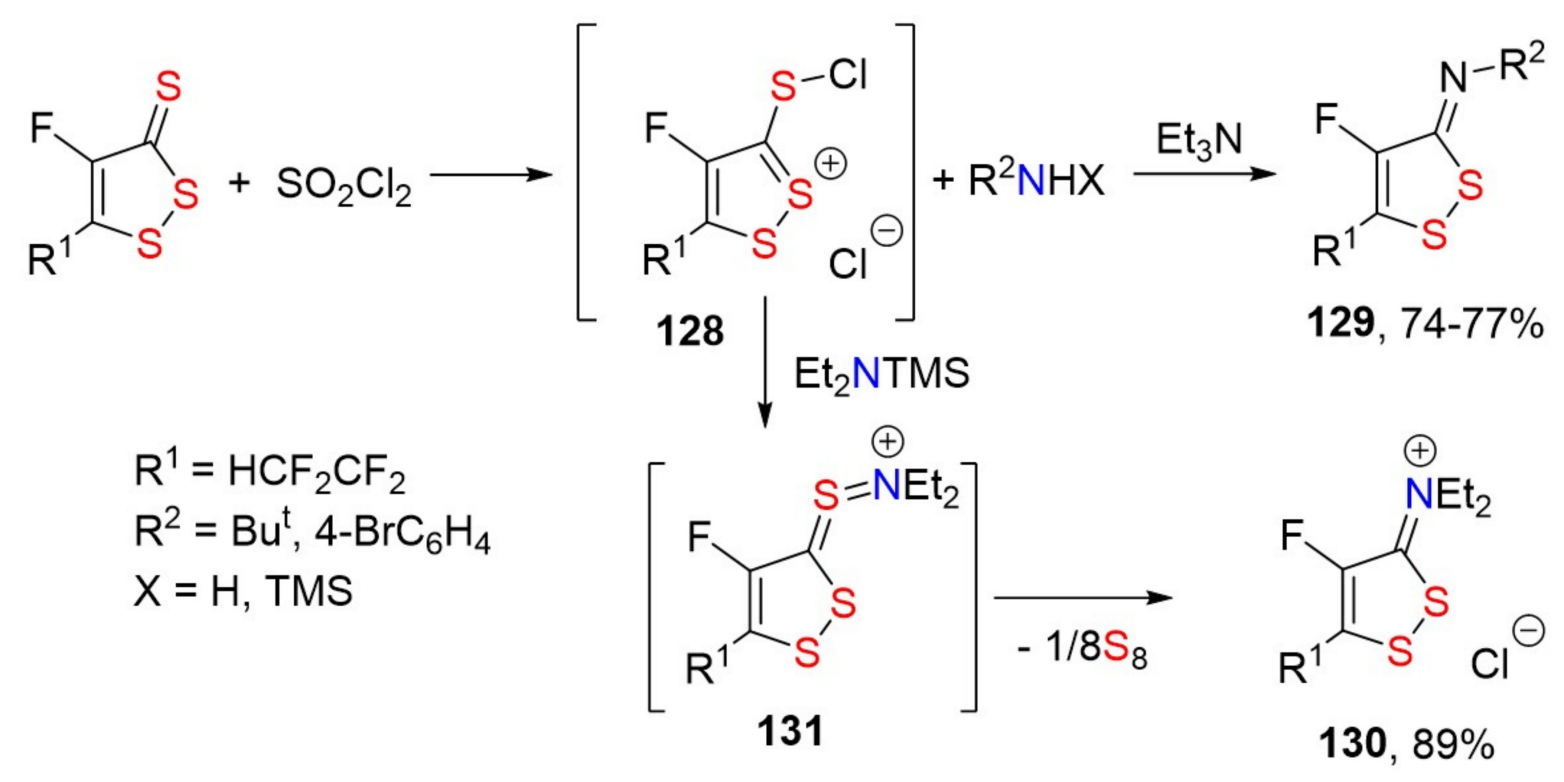
Reaction of dithiolium salts with amines.

The 1,2-dithiolium salts reacted in situ with primary amines or their trimethylsilyl derivatives to give 1,2-dithiole-3-imines **129** in good yields [121]. The reaction of trimethylsilyldiethylamine with salt **128** gave the dithioloiminium salt **130** in an even higher yield (Scheme 76). The authors suggested that its formation occurred through the intermediate salt **131** from which a sulfur atom was extruded.

### 3.9. Reaction of 1,2-dithiole-3-thiones with N-Nucleophiles

**Scheme 77:**
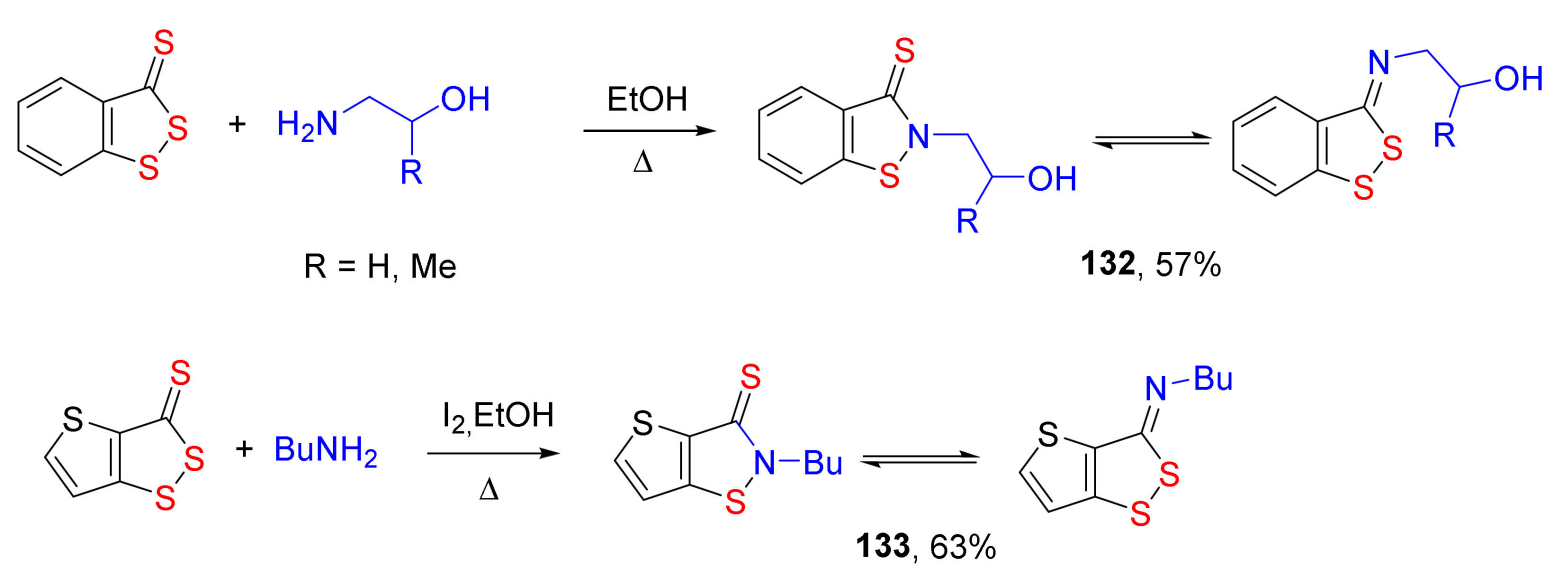
Reaction of fused 1,2-dithioles with amines.

The reaction of 1,2-dithiole-3-thiones with amines has been studied for a long time. It was reported that the imine function could replace both endocyclic (in the first and second positions) and exocyclic sulfur atoms. However, this reaction has not yet been clarified. In fact, 1,2-dithiole-3-thiones fused with aromatic and heteroaromatic rings were studied in reactions with aliphatic amines in ethanol (Scheme 77) [122,123]. In both cases, the authors suggested that the reactions resulted in inseparable mixtures of regioisomers **132** and **133**, in which the exocyclic sulfur atom at position 2 and the endocyclic sulfur atom, respectively, were replaced. The yields were moderate in both cases.

**Scheme 78:**
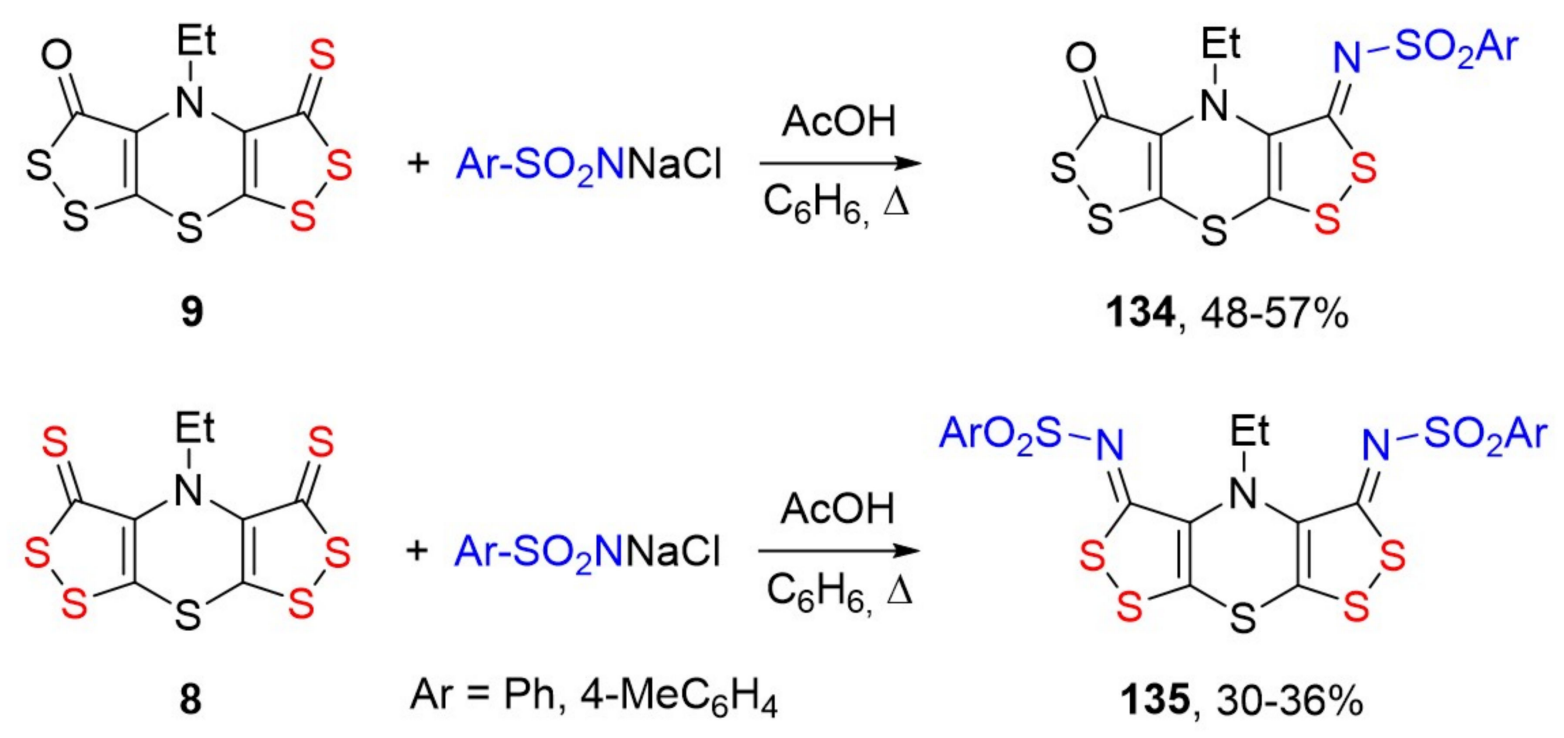
Reaction of bis(dithiolo)thiazines **8** and **9** with chloroamines B and T.

The formation of 1,2-dithiole-3-imines from 1,2-dithiole-3-thiones can occur in the reactions of the latter with chloramines B and T. It was found [60] that the reaction of tricyclic dithiolethiones **9** and **8** with these reagents in benzene in the presence of acetic acid gave mono- **134** and bis-imines **135** in moderate yields (Scheme 78). Replacement of acetic acid with a Lewis acid (scandium triflate) significantly increased the reaction rate and the yields of the final products. For example, the reaction of **9** with chloramines B and T in the presence of scandium triflate in DCM at room temperature for 5–15 min afforded imines **135** in 73 and 89% yields, respectively.

**Scheme 79:**
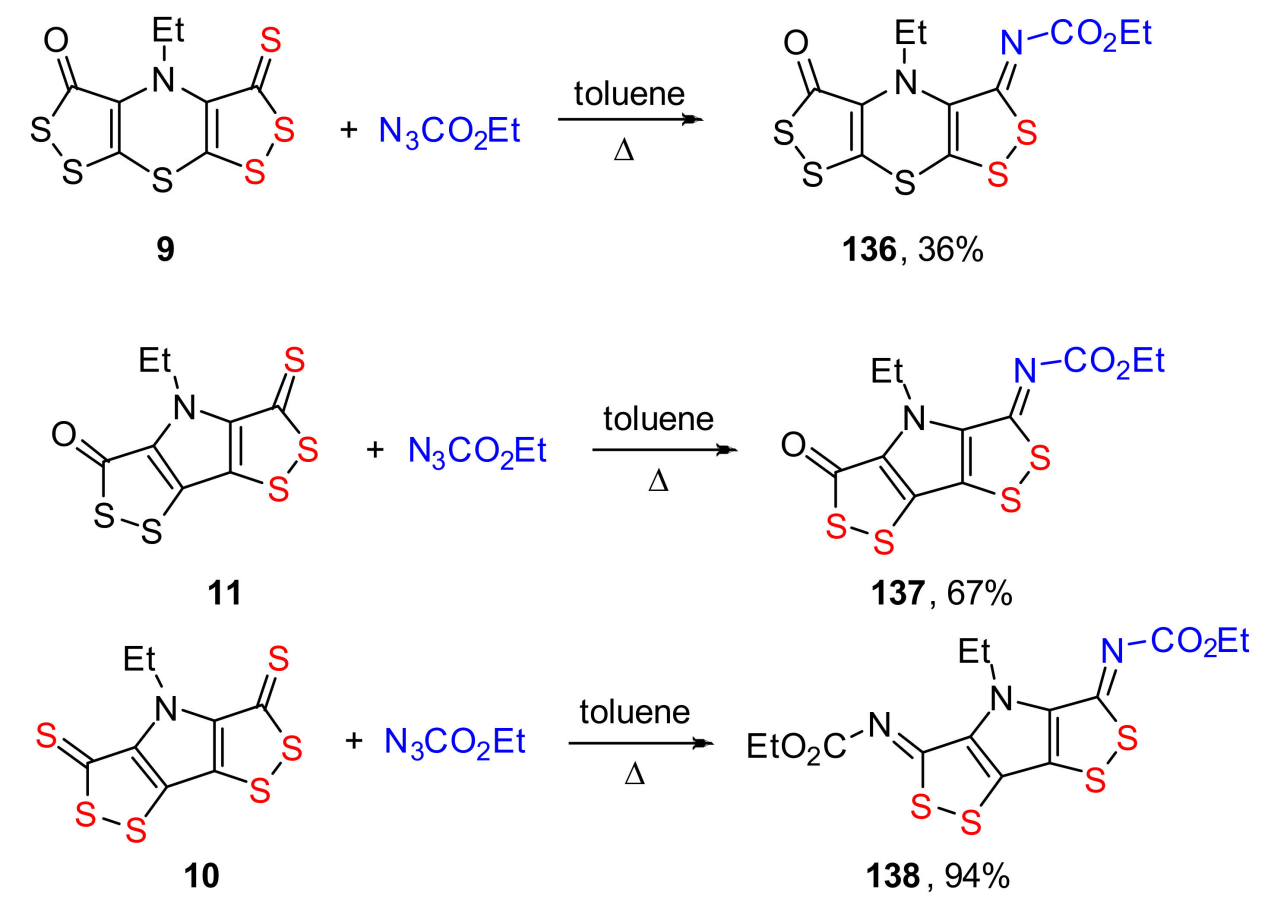
Reaction of bis(dithiolo)thiazines and bis(dithiolo)pyrroles with ethoxycarbonyl azide.

Organic azides are yet another reagents that can convert 1,2-dithiole-3-thiones to 1,2-dithiole-3-imines. It was found that ethoxycarbonyl azide gave stable imines in the reaction with fused 1,2-dithiole-3-thiones **9**–**11** (Scheme 79). The reaction was carried out in refluxing toluene, and the yields of imines **136**–**138** varied greatly from low to almost quantitative values [92]. The mechanism of formation of these imines can be represented as a 1,3-dipolar cycloaddition of an azide to a thione group, followed by the extrusion of a nitrogen molecule to give a thiaziridine derivative, which in turn extruded a sulfur atom to give the final imines **136**–**138**.

**Scheme 80:**
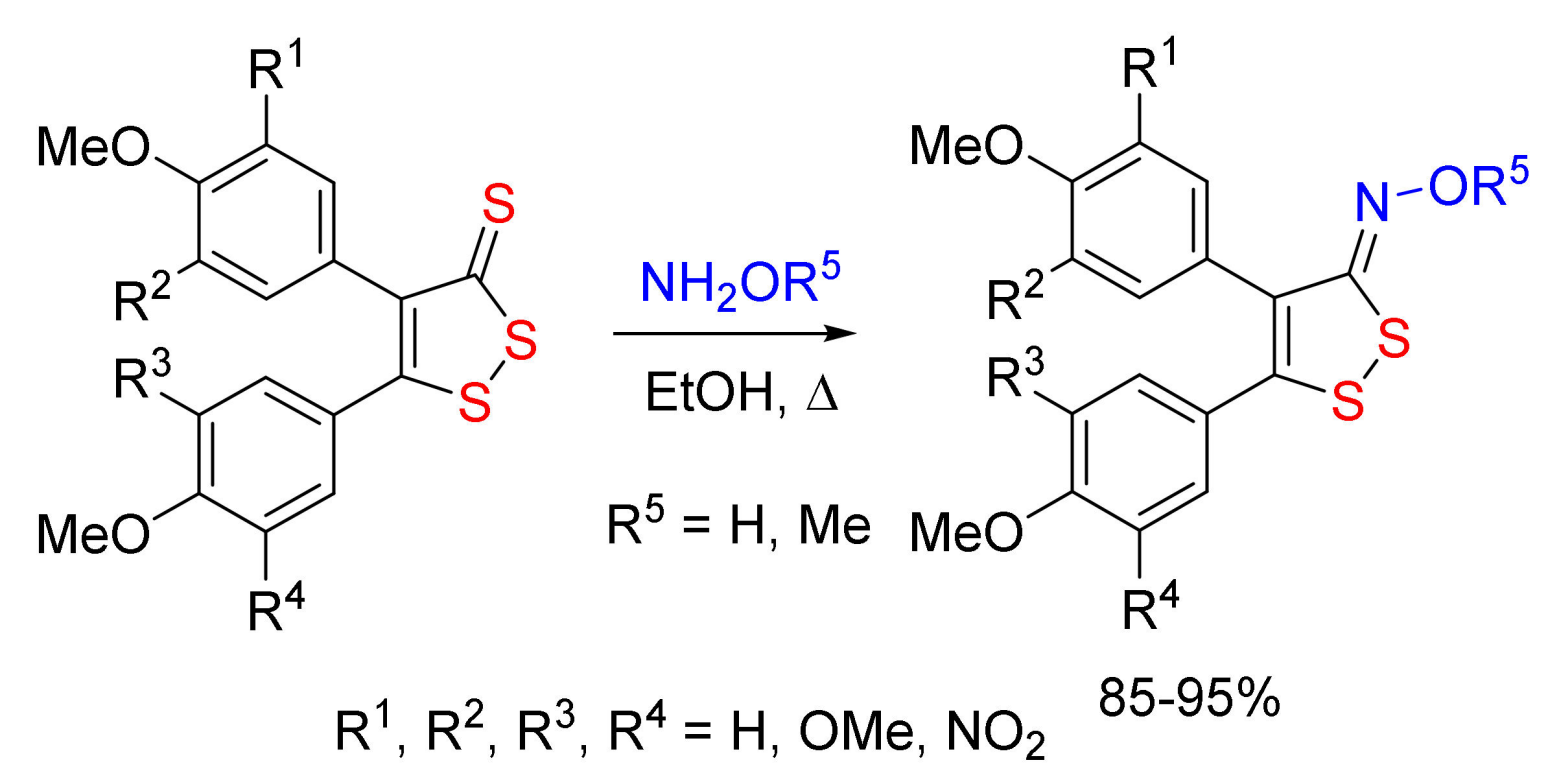
Reaction of 4,5-diaryl-3*H*-1,2-dithiole-3-thiones with hydroxylamines.

The oximine group can be easily incorporated into the 1,2-dithiole ring by the reaction of 4,5-diaryl-3*H*-1,2-dithiole-3-thiones with hydroxylamine or its *O*-methyl derivative in refluxing ethanol (Scheme 80) [29].

**Scheme 81:**
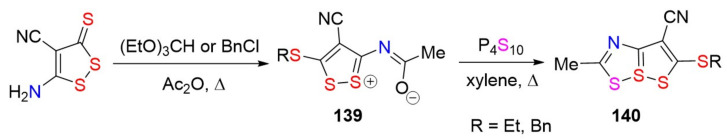
Alkylation of 4-cyano-5-amino-1,2-dithiol-3-thione.

Alkylation of 4-cyano-5-amino-1,2-dithiol-3-thione with ethyl orthoformate or benzyl chloride in acetic anhydride resulted in products in which, along with the formation of a salt structure, the primary amino group was acylated and intermediate 1,2-dithiolium betaines **139** formed [119]. Their conversion to tritiaazapentalenes **140** occurred on treatment with P_4_S_10_ in xylene (Scheme 81).

### 3.10. Synthesis of 3-alkylidene-3H-1,2-dithioles

**Scheme 82:**
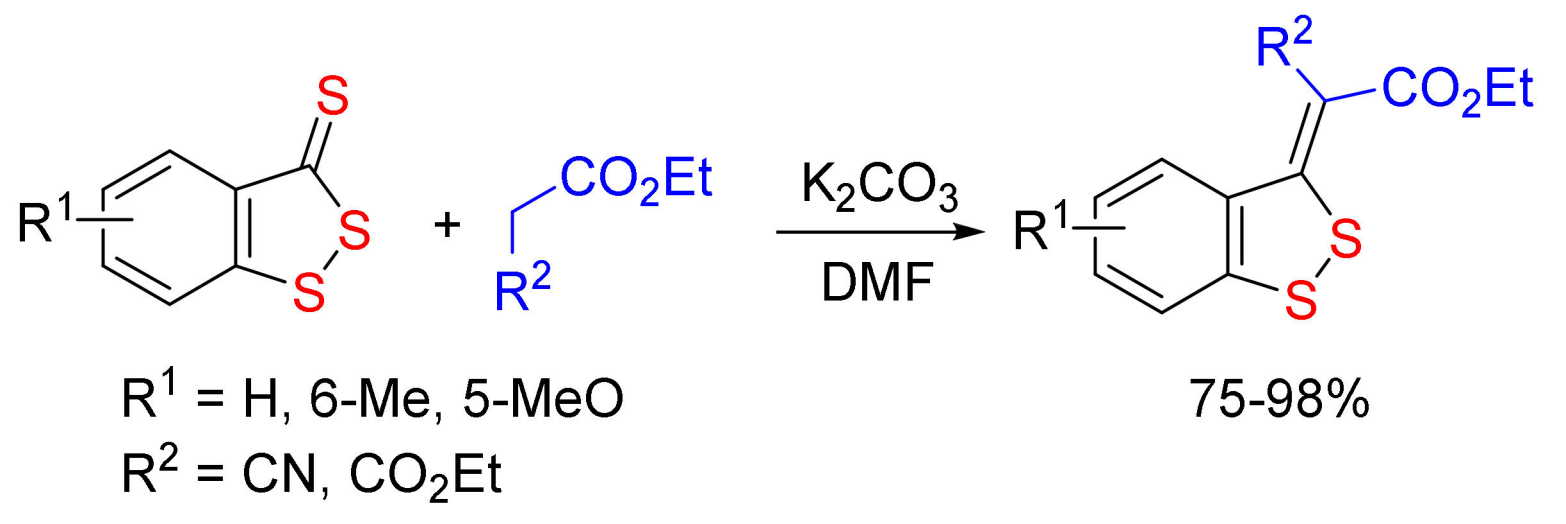
Knoevenagel reactions of substituted 3*H*-benzo[1,2]dithiole-3-thiones.

Knoevenagel reactions of substituted 3*H*-benzo[1,2]dithiole-3-thiones with reactive methylene compounds such as ethyl 2-cyanoacetate and diethyl malonate in DMF in the presence of K_2_CO_3_ under mild conditions gave alkylidene-3*H*-1,2-dithioles in good-to-excellent yields (Scheme 82) [73].

**Scheme 83:**
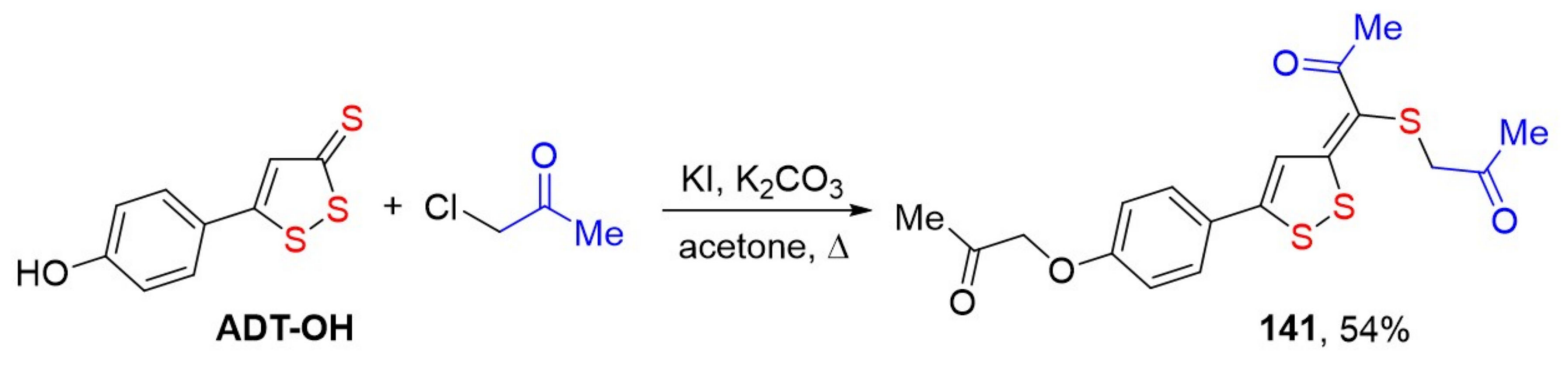
Alkylation of ADT-OH.

Alkylation of **ADT-OH** with excess 1-chloropropan-2-one in the presence of KI and K_2_CO_3_ gave, instead of the expected nucleophile reaction, a product with two acetyl groups whose structure was unambiguously proved to be (*E*)-5-[4-(2-oxopropyloxy)phenyl]-3-[2-oxo-1-(2-oxopropylthio)propylidene]-3*H*-1,2-dithiole **141** by means of X-ray diffraction (Scheme 83). The formation of this product was explained by a two-step process involving the formation of a 1,2-dithiolium salt [124].

**Scheme 84:**
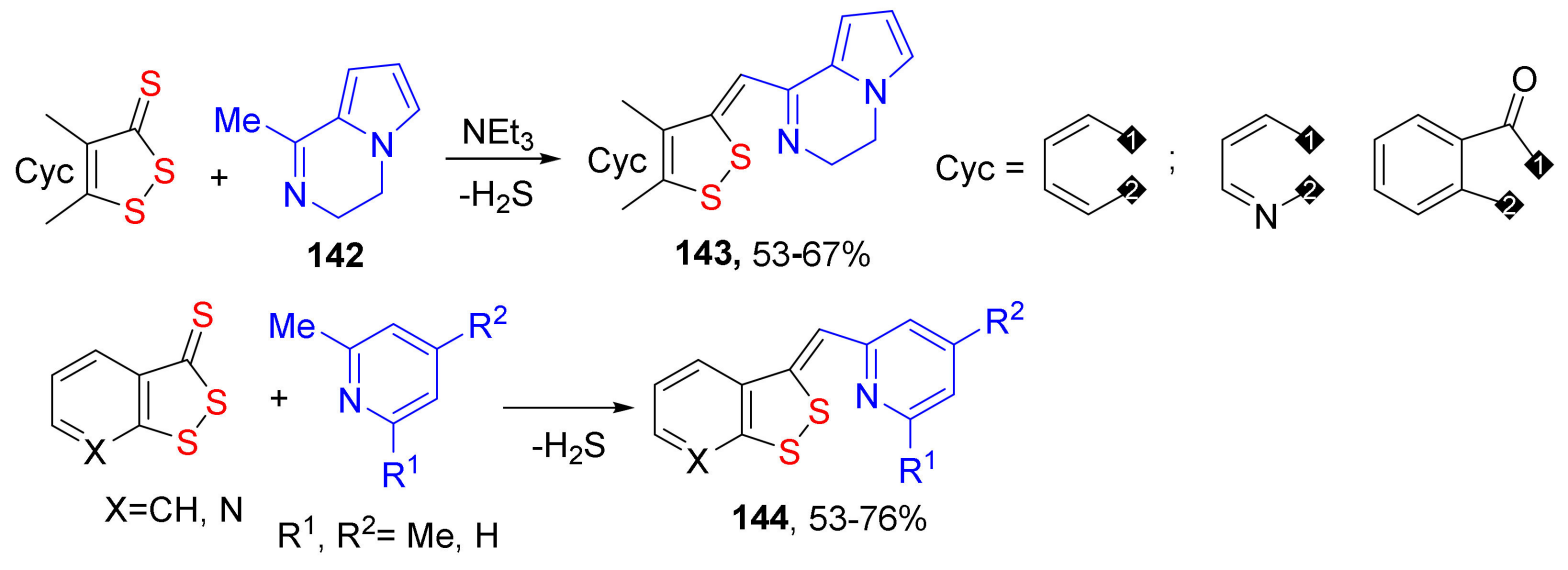
Reaction of 1-methyl-3,4-dihydropyrrolo[1,2-*a*]pyrazine and 2-methylpyridines with 1,2-dithiole-3-thiones.

Treatment of 1,2-dithiole-3-thiones with 1-methyl-3,4-dihydropyrrolo[1,2-*a*]pyrazine **142** in the presence of triethylamine afforded compounds containing a 1,2-dithiolo-3-ylidene moiety **143**. The reaction was accompanied by the evolution of hydrogen sulfide that was detected by the blackening of a lead indicator paper [125]. Fused 1,2-dithiole-3-thiones reacted similarly with 2-methylpyridines used as the solvent to form the corresponding ylidene derivatives **144** in moderate yields (Scheme 84).

### 3.11. Miscellaneous Reactions

**Scheme 85:**
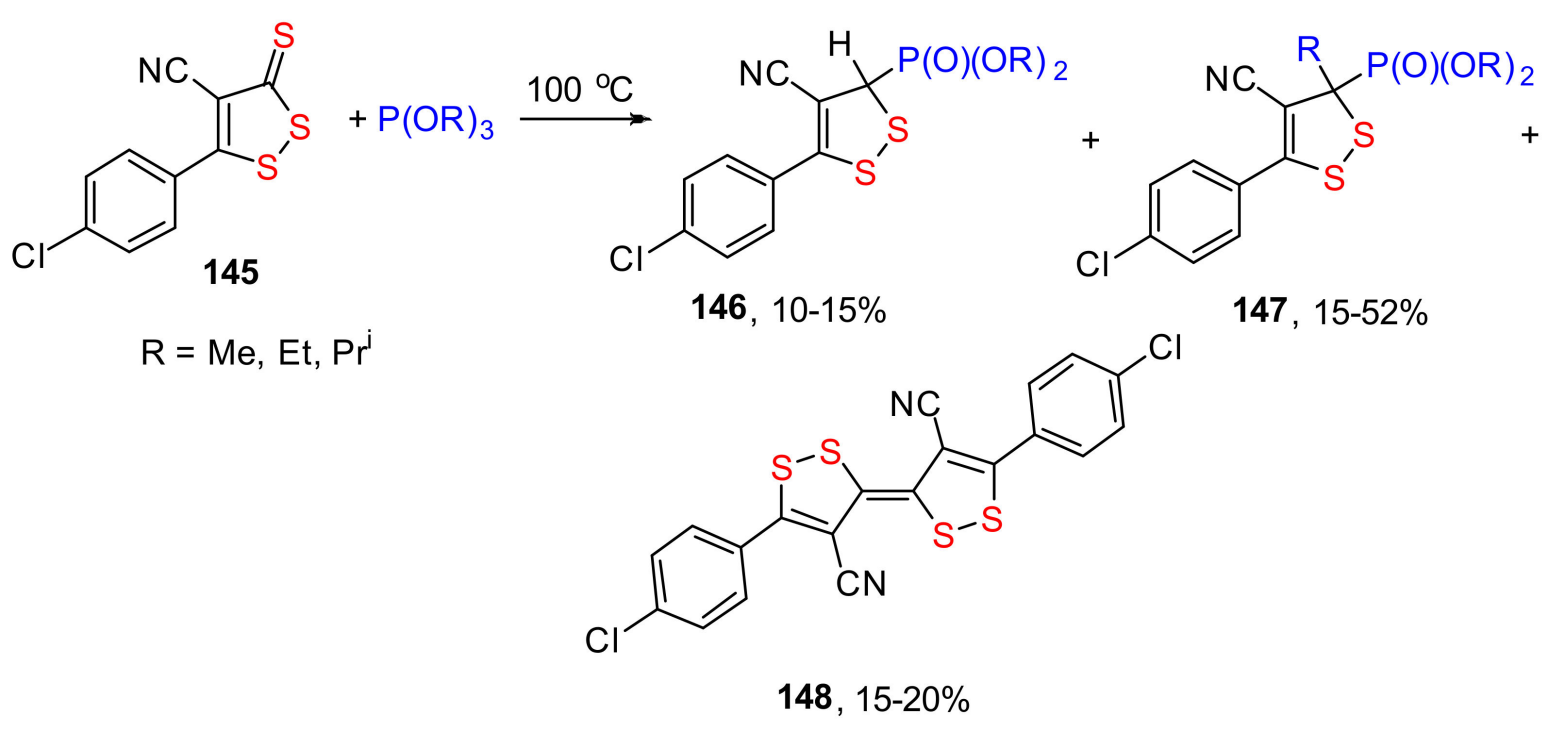
Reaction of 4-cyano-5-aryl-3*H*-1,2-dithiole-3-thione **145** with trialkyl phosphites.

Treatment of 4-cyano-5-aryl-3*H*-1,2-dithiole-3-thione **145** with trialkyl phosphites [126] gave a number of products: two of them (**146** and **147**) are products of thione group replacement for a phosphorus-containing moiety, and the third one, **148**, is the product of 1,2-dithiole dimerization (Scheme 85). The authors provided complex mechanisms of their formation but gave no serious evidence of the structure of these products.

**Scheme 86:**
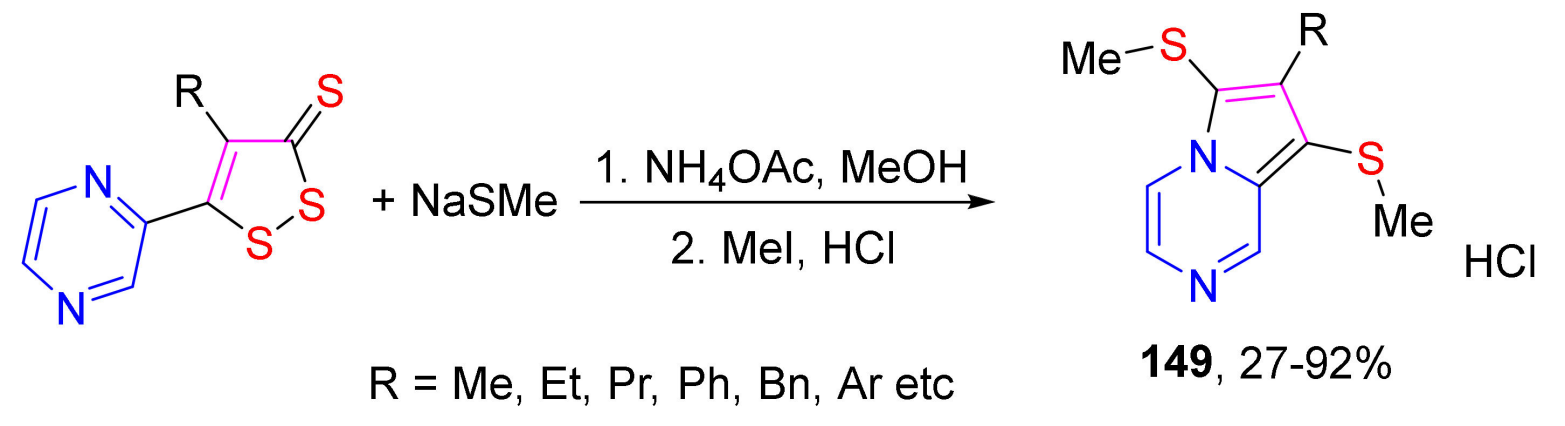
Reaction of Oltipraz and its derivatives with sodium methoxide.

Treatment of Oltipraz or its derivatives with sodium methoxide in MeOH afforded pyrrolo[1,2-*a*]pyrazines **149** [30,127]. Methyl iodide was added at the last reaction stage to stabilize the thiolo groups, which formed at the first step, to methylthio derivatives (Scheme 86). Thiomethoxide was assumed to attack C-4 instead of S-2 and the subsequent ring closure resulted in the final bicycle **149**. These results are of interest in biological context as they may contribute to the understanding of the pharmacological activity of Oltipraz.

## 4. Conclusions

The review covers the latest data on the synthesis and reactivity of 3*H*-1,2-dithiole-3-thiones which have been intensely studied for about 150 years. The reason of the importance of these heterocycles in these days is that the 1,2-dithiole-3-thione moiety is present in a number of commercial drugs such as Oltipraz, Anethole dithiolethione, S-Danshensu, and NOSH-1. The most recent interest in molecules containing the 1,2-dithiole-3-thione moiety is associated with the discovery of their ability to endogenously generate hydrogen sulfide. The 1,2-dithiole-3-thione ring can be successfully built by sulfuration of 3-oxoesters, α-enolic dithioesters or α-enolic dithioic acids, allylic methyl derivatives, tertiary isopropylamines, and alkynes. Recent advances in the synthesis of 1,2-dithiole-3-thiones include three efficient approaches via tandem sulfuration/annulation of propargylamines, defluorinative thioannulation of trifluoropropynes with sulfur, and [3+2] cycloaddition of elemental sulfur to cyclopropenthione derivatives. Some important ring transformations of 1,2-dithiole-3*H*-thiones include the 1,3-dipolar cycloaddition to alkynes, isonitriles and nitrilimines, and recyclization to other heterocyles, which resulted in a series of heterocyclic systems, namely, 1,3-dithioles, spiro-1,3-dithiolothiopyrans, 1,3-dithiethanes, 1,3,4-thiadiazolines, and pyrrolo[1,2-*a*]pyrazines. In recent years, a number of new transformations of 1,2-dithiole-3*H*-thiones have been discovered, including the replacement of one or two endocyclic sulfur atoms with carbon- or carbon-nitrogen containing moieties to give important sulfur-containing heterocyclic systems, such as pyrazino[2,1-*b*][1,3]thiazines, thienothiopyranethions, tritiapentalenes, 1,3-dithiine-4-thiones, and many others. I hope that this review will give strong impetus to the development of this scientifically interesting and practically important field of heterocyclic chemistry.
